# NR2F6, a new immune checkpoint that acts as a potential biomarker of immunosuppression and contributes to poor clinical outcome in human glioma

**DOI:** 10.3389/fimmu.2023.1139268

**Published:** 2023-07-28

**Authors:** Hayat Miftah, Oumayma Naji, Saadia Ait Ssi, Amina Ghouzlani, Abdelhakim Lakhdar, Abdallah Badou

**Affiliations:** ^1^ Immuno-Genetics and Human Pathology Laboratory, Faculty of Medicine and Pharmacy, Hassan II University, Casablanca, Morocco; ^2^ Department of Neurosurgery, University Hospital Center (UHC) Ibn Rochd, Casablanca, Morocco; ^3^ Laboratory of Research on Neurologic, Neurosensorial Diseases and Handicap, Faculty of Medicine and Pharmacy, Hassan II University, Casablanca, Morocco; ^4^ Mohammed VI Center for Research and Innovation, Rabat, Morocco; ^5^ Mohammed VI University of Sciences and Health, Casablanca, Morocco

**Keywords:** NR2F6, glioma, immunotherapy, glioma progression, immune and stromal infiltration, immunosuppression, clinical outcome

## Abstract

**Intoroduction:**

Nuclear receptor subfamily 2 group F member 6 (NR2F6) is a promising checkpoint target for cancer immunotherapy. However, there has been no investigation of NR2F6 in glioma. Our study systematically explored the clinical characteristics and biological functions of NR2F6 in gliomas.

**Methods:**

We extracted RNA sequencing (RNA-seq) data of 663 glioma samples from The Cancer Genome Atlas (TCGA) as the training cohort and 325 samples from the Chinese Glioma Genome Atlas (CGGA) as the validation cohort. We also confirmed the NR2F6 gene expression feature in our own cohort of 60 glioma patients. R language and GraphPad Prism softwares were mainly used for statistical analysis and graphical work.

**Results:**

We found that NR2F6 was significantly related to high tumor aggressiveness and poor outcomes for glioma patients. Functional enrichment analysis demonstrated that NR2F6 was associated with many biological processes that are related to glioma progression, such as angiogenesis, and with multiple immune-related functions. Moreover, NR2F6 was found to be significantly correlated with stromal and immune infiltration in gliomas. Subsequent analysis based on Gliomas single-cell sequencing datasets showed that NR2F6 was expressed in immune cells, tumor cells, and stromal cells. Mechanistically, results suggested that NR2F6 might act as a potential immunosuppression-mediated molecule in the glioma microenvironment through multiple ways, such as the recruitment of immunosuppressive cells, secretion of immunosuppressive cytokines, M2 polarization of macrophages, in addition to combining with other immune checkpoint inhibitors.

**Conclusion:**

Our findings indicated that intracellular targeting of NR2F6 in both immune cells and tumor cells, as well as stromal cells, may represent a promising immunotherapeutic strategy for glioma. Stromal cells, may represent a promising immunotherapeutic strategy for glioma.

## Introduction

Glioma is the most aggressive and lethal type of the central nervous system that originates from neuroglial stem or progenitor cells, with glioblastoma (GBM) being the worst malignant subtype (1). According to the world health organization (WHO), glioma is categorized into oligodendroglioma, astrocytoma, glioblastoma, and mixed gliomas (2); and classified into low grade glioma (I and II) and high grade glioma (III and IV) (3). Despite multimodal treatment approaches consisting of maximal surgical resection, followed by external radiotherapy with concomitant and adjuvant Temozolomide, there has been relatively little improvement, with a median overall survival (OS) of less than 15 months, 7 to 8 months of median progression-free survival (PFS), and a 5-year rate of only 6.8% (4–8). This is profoundly associated with heterogeneous tumors, with different regions of the tumor exhibiting distinct cellular and molecular features which facilitate immune evasion (9, 10). Moreover, high level of immunosuppressive cell infiltration (e.g., regulatory T cell (Treg), tumor-associated microglia, tumor-associated macrophages, and myeloid-derived suppressor cells) and a high prevalence of exhausted T cells represent a significant barrier to immunotherapies in GBM (11–16). Although, different immunotherapeutic modalities have been combined with conventional therapies to enhance the clinical outcomes of GBM patients, including oncolytic virus, checkpoint inhibitors, vaccines, T-cell therapy, adoptive T-cell transfer, and chimeric antigen receptor (CAR) (17), currently, a small percentage of patients experience more durable responses and are still alive two years following diagnosis, and in fewer cases, they survive even longer (7, 18, 19). Moreover, immunotherapy is assumed to elicit an anti-tumor response, PD-1 and CTLA-4 inhibition have shown significant effectiveness in treating several solid cancers, such as melanoma and lung cancer (20–22). However, GBM patients are refractory to current immunotherapies assessing nivolumab, an anti-programmed cell death protein 1 (anti–PD-1) therapy, alone or in combination with radiotherapy and temozolomide (6, 23). According to recent reports, immunotherapy failure may be related to upregulation of various immune checkpoints in glioma patients after blocking the PD1/PDL-1 pathway (24), indicating the importance of deciphering novel biomarkers for an additive or synergistic impact on glioblastoma patients to guide and improve immune-mediated therapy concepts (25, 26).

Recently, NR2F6 has attracted particular interest as a potential novel immune checkpoint receptor target (27). NR2F6 acts as a transcriptional repressor in different cell subsets, such as Th0, Th17, CD4 T cells, and CD8 T cells, by antagonizing the DNA accessibility of NFAT and AP-1 transcription factors through direct binding to multiple regions within key cytokine promoter loci such as IL-2, IFNγ, and TNFα (28). Different research has provided an overview regarding the proper contribution of NR2F6 in the immune response. Overall, NR2F6 plays a crucial role in cellular homeostasis and various diseases, including cancer (29–32). NR2F6 has been reported to be ubiquitously weakly expressed in resting T cells and highly expressed in effector T cells, where it triggers an anti-inflammatory response (27). Moreover, NR2F6 is overexpressed in a variety of malignancies, including lymphoma, head and neck squamous cell carcinoma, acute myeloid leukemia (AML), colon cancer, and breast cancer (33–37). Consequently, the researchers found that NR2F6 expression appears to be associated with quicker tumor progression and worse overall patient survival (27, 38). Furthermore, evaluating tumor-infiltrating lymphocytes from non-small cell lung cancer (NSCLC) patients’ biopsies provides substantial preclinical evidence that NR2F6 overexpression at the tumor niche produces effector T cells unable to mount a robust immune response against malignancy (39). Interestingly, genetic knockout of NR2F6 significantly improves responses to PD-1/PDL-1 cancer immune checkpoint inhibition (39).

As a promising intra-cellular immune checkpoint inhibitory, NR2F6 might be a good target for immunotherapy besides those present on the cell surface. Nevertheless, a rigorous assessment of NR2F6 involvement in glioma patients has yet to be handled. Therefore, we set out to explore NR2F6 mRNA profiling in glioma through 1048 samples. The RNA-sequencing dataset from the Cancer Genome Atlas (TCGA) was used as a training cohort, and our results were validated in an independent cohort using the Chinese Glioma Genome Atlas (CGGA) dataset and the Moroccan glioma patient cohort. The present study is the first to clinically, molecularly, and immunologically characterize NR2F6 expression in gliomas.

## Materials and methods

### Clinical samples

A total of 60 tumor samples from glioma patients were collected from the Ibn Rochd University Hospital, neurosurgery department from May 2016 to April 2022 (30 specimens of high-grade glioma: 22 glioblastomas, 3 astrocytomas grade III, and 5 ependymomas grade III and 30 specimens of low-grade glioma: 19 astrocytomas grade I, 2 astrocytomas grade II, 3 oligoastrocytomas grade II, 5 ependymomas grade II, and 1 xantoastrocytoma grade II) (**Table Supplementary 2**). All patients underwent surgery, and fresh tumor tissue was obtained during surgery. Moreover, none of them received any chemotherapy or radiotherapy before tumor resection. Signed informed consent forms were obtained from all subjects. The Ethical Board of the Ibn Rochd University Hospital of Casablanca approved this study.

### Public data acquisition and preprocessing

From the TCGA dataset, RNA sequencing expression data and the clinicopathological characteristics from 663 glioma samples (glioblastoma (GBM) 150 cases, low-grade glioma (LGG) 513 cases) (**Table Supplementary 1**), were analyzed in our study (http://cancergenome.nih.gov). In order to corroborate the findings that we have revealed in the TCGA dataset, 325 glioma samples from the CGGA dataset were used as a validation cohort (**Table Supplementary 1**). CGGA transcriptome sequencing data were generated using the Illumina Hiseq platform, which is publicly available (http://www.cgga.org.cn/). The Limma package (40) of R software was utilized for the normalization of RNA expression profiles and the batch effect between TCGA-LGG samples and TCGA-GBM samples was corrected using the SVA package (41).

We established the following criteria for patients screening: WHO Grade II-III-IV, IDH mutation status, sex, age, histopathological type, survival status and overall survival data. As long as the six types of data mentioned above were available, we would include these subjects in this study, and there are no additional exclusion criteria.

### RNA extraction and cDNA synthesis

Total RNA from 60 fresh biopsies was isolated using Trizol reagent (Invitrogen, France) (42, 43). We analyzed RNA concentration and purity with the use of a NanoVueTM Plus Spectrophotometer (GE Healthcare, UK). The samples were then diluted with ultrapure water to ensure that each tube had the same concentration of RNA. According to the manufacturer’s instructions, cDNA was synthesized from 1 μg of RNA included in a 20 μl reaction mixture containing RNase-Free Water Random Hexamer Primer (Bioline, France) and incubated at 70°C for 5 min. Afterward, 1 µL RNase-free water, 4 µL Tetro reverse transcriptase buffer, 0.5 µL RNase inhibitor (Invitrogen, France), 4 µL dNTP (10 mM), and 0.5 µL Tetro reverse transcriptase enzyme (Bioline, France) were added, followed by incubation at 25°C for 10 min, then at 45°C for 30 min, and finally at 85°C for 5 min.

### Real-time quantitative PCR

The expression levels of NR2F6 and β*-*Actin were assayed using fluorescence-based quantitative real-time PCR (RT-qPCR) (SYBR Green PCR Master Mix; (Thermo Fischer)). A reagent mixture of 18 µL (7 µL ultra-pure water, 0,5 µL of each primer sequence (forward and reverse), and 10 µL SYBR Green) besides 2 µL of cDNA were added to each well of the PCR plate. Instead of cDNA, 2 µL of ultra-pure water were used in the negative control well. The genes were amplified under the following conditions: hold stage at 95°C for 10 min, followed by 40 cycles of denaturation at 95°C for 15 s, and annealing and extension at 60°C for 1 min. The relative expression level was calculated using the 2^(−ΔCT) method described by Livak and Schmittgen (44) and the house-keeping gene *β*-Actin was used as an internal reference. At the end of the assay, a melting curve and electrophoresis were constructed to verify the specificity of the reaction.

The primers used for qPCR were as follows: β-actin, forward: 5′- GAGATGGCCACGGCTGCTT-3′ and reverse: 5′- GCCACAGGACTCCATGCCCA-3′, product length was 446 bp, instead of β-actin, forward:5′-TGGAATCCTGTGGCATCCATGAAAC-3′ and reverse: 5′-TAAAACGCAGCTCAGTAACAGTCCG-3′, product length was 144 bp.

### CIBERSORT

CIBERSORT is a deconvolution algorithm that characterizes the cell composition of complex tissue from their gene expression profiles (9, 45). This method enables the quantification of a specific cell type abundance and has been verified by fluorescence-activated cell sorting (FACS) (46). We used CIBERSORT (https://cibersortx.stanford.edu/) to assess the relative fractions of 22 tumor-infiltrating immune cell types in high and low NR2F6 expression groups, with the algorithm run using the LM22 signature matrix at 1000 permutations. These TIICs included 7 T-cell types (Tregs, naïve CD4+ T cells, CD8+ T cells, resting memory CD4+ T cells, T follicular helper (Tfh) cells, γδ T cells, activated memory CD4+ T cells), activated NK cells, resting natural killer (NK) cells, macrophages (M1 macrophages, M2 macrophages, M0 macrophages), monocytes, resting mast cells, resting dendritic cells (DC), activated DC, activated mast cells, memory B cells, naïve B cells, eosinophils, plasma cells, and neutrophils. The sum of all evaluated immune cell-type fractions equals 1 for each sample.

### xCell

xCell, reported by Aran (47), a method based on ssGSEA (single sample gene set enrichment analysis) that estimates the abundance scores of 64 cell types, was used to evaluate the proportion of the 12 types of stromal cell: Adipocytes, Endothelial cells, Chondrocytes, Fibroblasts, MSC, Osteoblast, Pericytes, Preadipocytes, Skeletal muscle, Smooth muscule, ly Endothelial cells, mv Endothelial cells and 34 types of immune cell: B-cells, CD4+ T-cells, CD4+ Tcm, CD4+ memory T-cells, CD4+ naive T-cells, CD8+ T-cells, CD8+ Tcm, CD8+ Tem, CD8+ naive T-cells, Class-switched memory B-cells, Memory B-cells, NK cells, NKT, Plasma cells, Tgd cells, Tregs, Th1 cells, Th2 cells, naive B-cells, pro B-cells, Basophils, Dendritic cells, Eosinophils, Macrophages, Macrophages M1, Macrophages M2, Mast cells, Monocytes, Neutrophils, Activated dendritic cells, Conventional dendritic cells, Immature dendritic cells, Plasmacytoid dendritic cells.

### Single-cell level analysis

We obtained GBM single-cell sequencing data (GSE102130, GSE103224, GSE135437, GSE138794, GSE141383, GSE141982, GSE148842, GSE162631, GSE163108, GSE70630, GSE139448, GSE131928) Based on the Tumor Immune Single-Cell Hub (TISCH) online database (http://tisch.comp-genomics.org/) (48, 49), which was used to classify stromal cells, immune cells, and malignant cells by hierarchical clustering. Then, NR2F6 expression in these cells was evaluated, and the results were illustrated by heatmaps.

### Identification of differentially expressed genes

The differentially expressed genes between low-NR2F6 and high-NR2F6 were performed using limma package with the voom function (40). We removed genes with low expression levels to correct the batch effect. We used the calcNormFactors function to calculate the normalization factor for each patient and the voom function to perform CPM normalization, adjusted by the TMM method. Quantile normalization was used to normalize RNA-seq data. The results are presented as a table of genes ordered by significance (**Table Supplementary 3**, **4**), and a |log fold change (FC)| > 0.4 and adj. *P* value < 0.05 were further conducted as the cutoff criteria for the DEGs screening. The GO enrichment analysis of DEGs was shown by heatmap using the visual hierarchical cluster analysis by the web-based Morpheus software (https://software.broadinstitute.org/morpheus/). VennDiagram package in R software was used to identify overlapping DEGs between TCGA and CGGA.

### Gene functional and pathway enrichment analysis

**The biological functions and signaling pathways related to NR2F6 were explored by** Gene ontology **(GO) and** Kyoto Encyclopedia of Genes and Genomes **(KEGG) analyses using the** Database for Annotation, Visualization, and Integrated Discovery (DAVID) (https://david.ncifcrf.gov) (50–52). Terms with p-value <0.05 was considered significantly enriched.

### Statistical analysis

The statistical software R (version 4.0.3) and GraphPad Prism 8 software (version 8.0.2) were used for the statistical analysis and generation of figures. The median value of NR2F6 expression was considered as the cutoff value to separate patients into the high and low groups. Survival analysis was conducted using Kaplan-Meier analysis and the log-rank test. The Wilcoxon Signed Rank test, Mann-Whitney and unpaired t-test were used for statistical analysis between two groups, while the Kruskal-Wallis test was applied to statistical analysis between more than two groups. Non-parametric Spearman test was conducted to evaluate the correlation of two variables. All statistical tests were independently performed by two different scientists, and a p-value less than 0.05 was considered statistically significant.

## Results

### High NR2F6 expression was related to higher tumor malignancy in glioma

**Figure 1:**
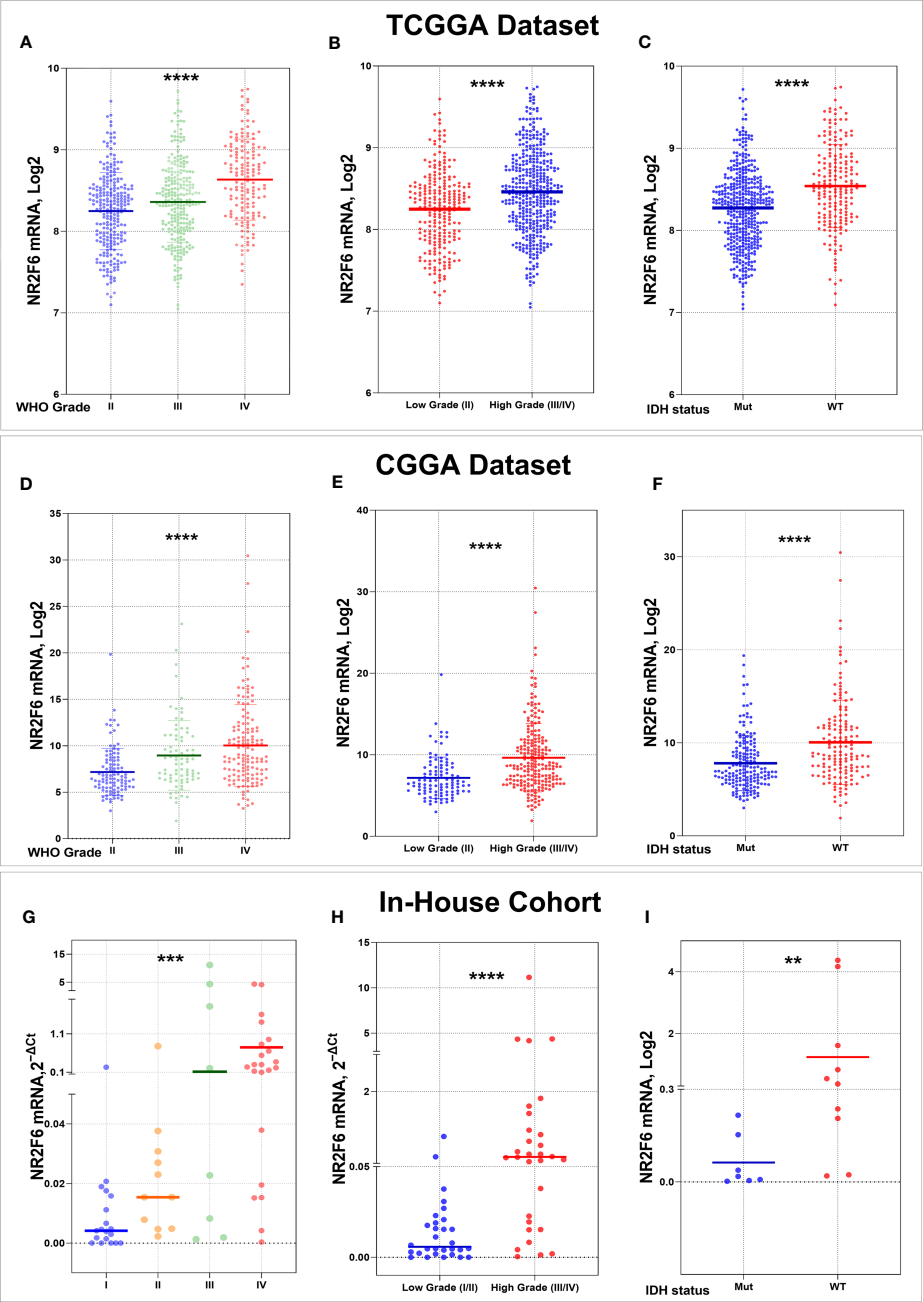
Association of NR2F6 expression with clinical glioma parameters in TCGA, CGGA databases, and in in-house cohort. NR2F6 expression level significantly increases with increasing tumor grade in gliomas **(A, D, G)**. The NR2F6 gene is strongly expressed in high grade compared with low grade glioma tissues **(B, E, H)**. NR2F6 expression is significantly enriched in IDH wild-type glioma (WT) compared with IDH mutant (Mut) **(C, F, I)**. In all statistical analyses, p values less than 0.05 were considered statistically significant, **p<0.01; ***p<0.001; ****p<0.0001.

To explore the expression pattern of NR2F6 in glioma, we first assessed NR2F6 expression in 663 RNA sequencing samples from the TCGA database, according to glioma grades. The NR2F6 expression level increased with increasing tumor grade (**Figure 1A**). The higher expression of NR2F6 was significantly observed in high-grade versus low-grade glioma tissues (**Figure 1B**). We also validated our findings in CGGA database (**Figures 1D, E**), as well as in the in-house cohort (**Figures 1G, H**). IDH mutation status is a well-established clinically relevant molecular biomarker of glioma (53). Therefore, we analyzed the NR2F6 expression pattern based on IDH mutation status. NR2F6 expression was significantly up-regulated in IDH-wildtype gliomas than IDH-mutated gliomas in TCGA as well as CGGA and in-house cohort (**Figures 1C, F, I**). Taken together, these results indicated that NR2F6 expression was more prevalent in aggressive glioma. The correlation between NR2F6 expression and clinicopathological characteristics of patients with gliomas in the TCGA, CGGA, and in-house cohorts is presented in **Supplementary Tables 1**, **2**, respectively.

### NR2F6 expression was relevant to worse survival in glioma

**Figure 2:**
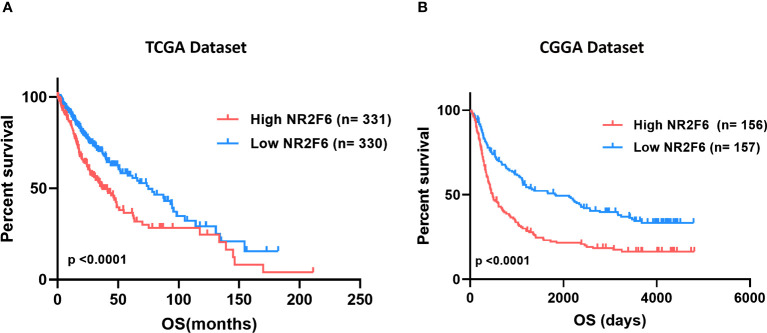
Survival analysis of glioma patients based on NR2F6 expression. Kaplan–Meier analysis indicated that high expression of NR2F6 was related to significantly worse prognosis in glioma patients, in TCGA **(A)** and CGGA **(B)** cohorts. Patients were divided into two groups based on the median value of NR2F6 expression. The red curve represents patients with high expression of NR2F6, and the blue curve represents patients with low expression of NR2F6.

As we discussed above, higher expression of NR2F6 was observed in higher grades of glioma, highlighting the possible relationship between NR2F6 expression and a poorer prognosis. Thus, we divided glioma patients into low and high-expression groups to evaluate NR2F6’s prognostic value. The Kaplan–Meier curves of the overall survival (OS) of patients with gliomas are illustrated in **Figure 2**. As shown, patients with glioma with lower NR2F6 expression exhibited significantly longer OS compared with patients with higher NR2F6 expression in both TCGA and CGGA cohorts (**Figures 2A, B**). These results indicated that high expression of NR2F6 conferred worse outcomes in glioma patients.

### Differential gene enrichment analysis between NR2F6 groups

**Figure 3:**
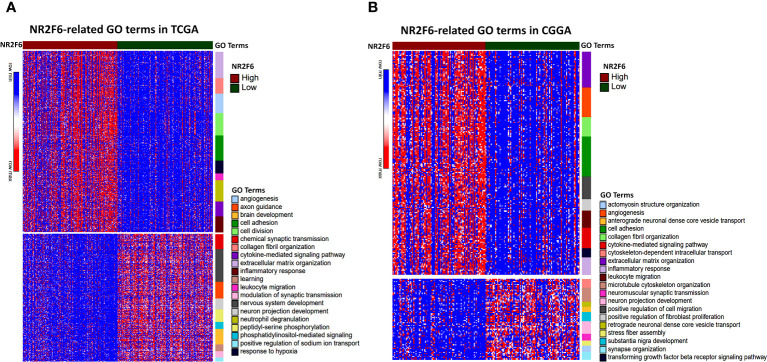
Biological function analysis for NR2F6 in glioma. NR2F6-related gene ontology (GO) terms in TCGA **(A)** and CGGA datasets **(B)**. Red to blue represents high to low DEG expression. The samples were ranked according to NR2F6 expression, from high (red color) to low (green color). The color bars at the right side of the heatmap represent the enriched gene ontology terms of upregulated and downregulated DEGs. DEGs, differentially expressed genes.

Since NR2F6 expression in glioma was strongly associated with malignancy, we inferred that NR2F6 may have important biologic functions in glioma. The GO functional analysis with DAVID was used to determine the biological role of NR2F6 in gliomas. First, we performed a differential gene analysis between low- and high-NR2F6 expression samples. According to adjusted *P* < 0.05 and |log_2_FoldChange| ≥ 0.4, 2159 genes were identified as DEGs in TCGA, of which 1127 were downregulated and 1032 were upregulated (**Supplementary Table 3**), and CGGA contained 772 DEGs, including 397 downregulated genes and 375 upregulated genes (**Supplementary Table 4**). GO Enrichment analysis of differential genes showed upregulation of many biological processes related to glioma progression such as extracellular matrix organization, collagen fibril organization, and angiogenesis. Of note, upregulated genes were also involved in several immune processes, such as leukocyte migration, cytokine-mediated signaling pathway, and inflammatory response. GO terms related to biological processes that are normal and indispensable, such as neuron projection development and nervous system development were downregulated. All the results mentioned above were shared by the two datasets (**Figures 3A, B**). In line with previous studies (27), these results indicate that NR2F6 might have dual pro-tumor activity in tumor cells and immune cells in the glioma microenvironment.

### NR2F6-related immune signatures in glioma

**Figure 4:**
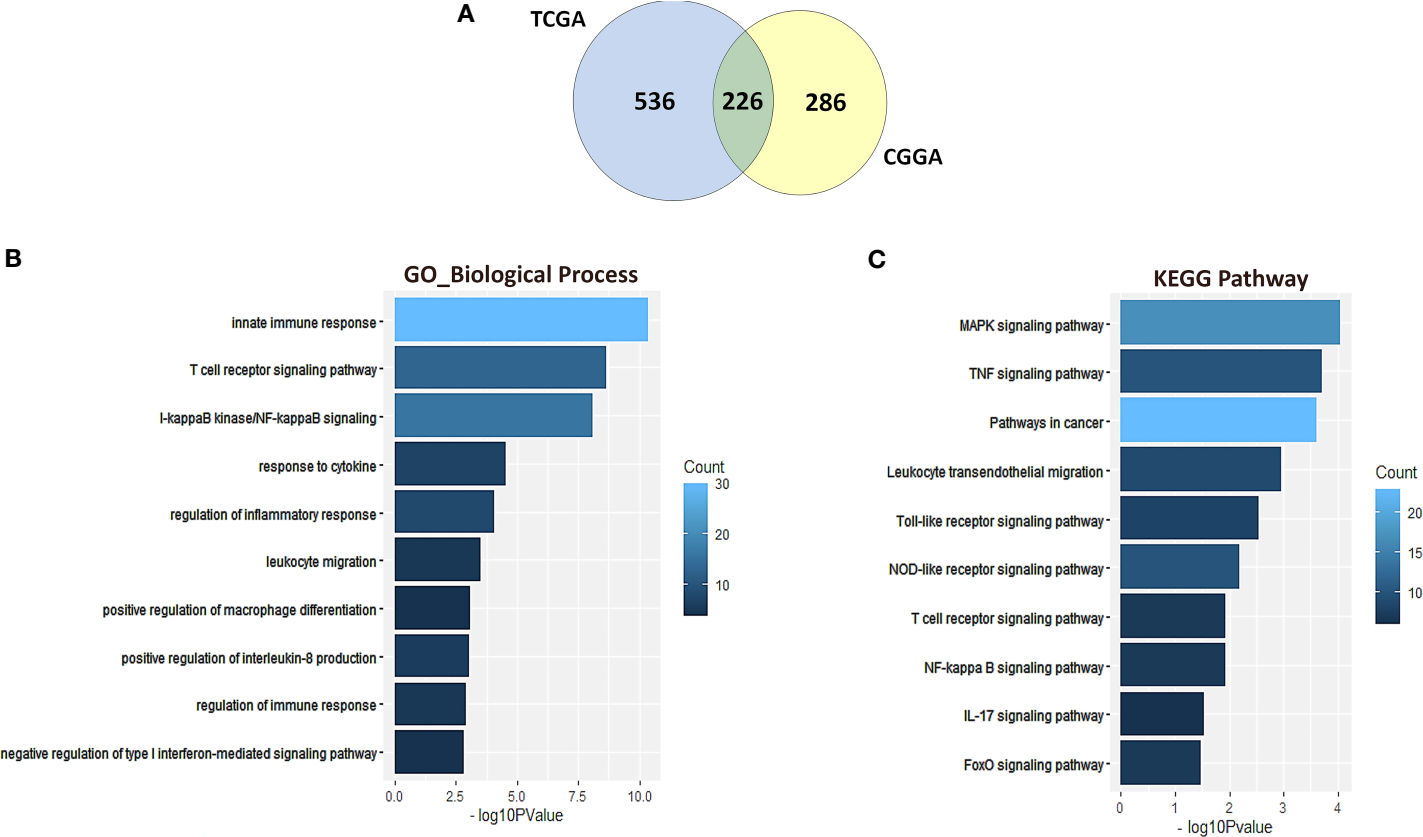
NR2F6-related immune signatures in glioma. A total of 226 common upregulated DEGs were identified from TCGA and CGGA datasets **(A)**. Gene ontology **(B)** and KEGG pathway analysis **(C)** of the 226 DEGs. The bar charts represented the P value and the color represented the count. The vertical axis represents the item name. KEGG, Kyoto Encyclopedia of Genes and Genomes.

To further identify the NR2F6-associated immune signature in glioma, we downloaded gene sets of the immune system from AmiGO 2 web portal (http://amigo.geneontology.org/amigo). We identified 226 overlapping upregulated DEGs in TCGA and CGGA datasets, which were ranked according to adjusted *P <*0.01 (**Figure 4A**). GO and KEGG enrichment analyses were used to clarify the biofunctions of these genes. The results showed that overlapping genes were highly enriched in innate immune response, T cell receptor signaling pathway, I-kappaB Kinase/NF-kappaB signaling, and response to cytokines in GO terms (**Figure 4B**). The KEGG pathway analysis suggested that NR2F6 may be involved in TNF signaling pathway, leukocyte transendothelial migration, Toll-like receptor signaling pathway, NOD-like receptor signaling pathway, and other immune-related pathways (**Figure 4C**). These findings suggest that NR2F6 may be associated with both innate and adaptive immune responses.

### NR2F6 was associated with infiltrating immune and stromal cells in glioma microenvironment

**Figure 5:**
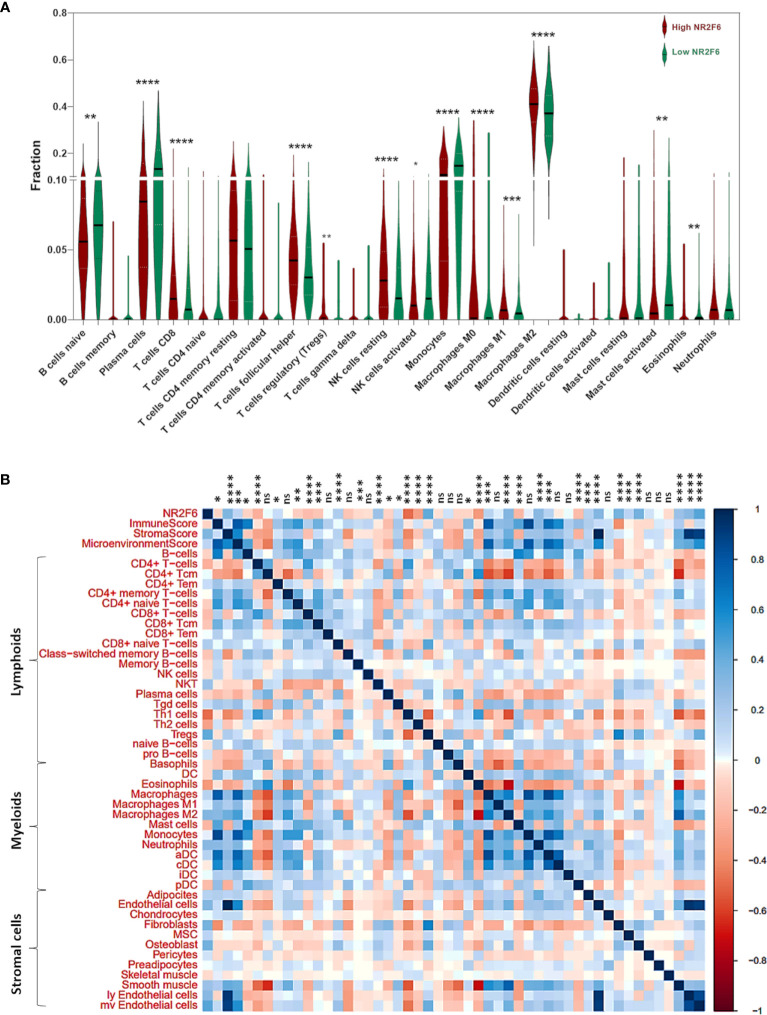
Analysis of tumor immune and stromal cell infiltration relative to the NR2F6 level in the TCGA dataset. **(A)** Proportions of the 22 types of tumor-infiltrating immune cells (TIICs) in different NR2F6 groups. **(B)** Correlation between NR2F6 expression and xCell scores in gliomas. Each colored square within the figure illustrates the correlation between NR2F6 and immune, stromal, and microenvironment scores and 46 cell types. blue, positive correlation; red, negative correlation. *p<0.05; **p<0.01; ***p<0.001; ****p<0.0001; ns, not significant.

**Figure 6:**
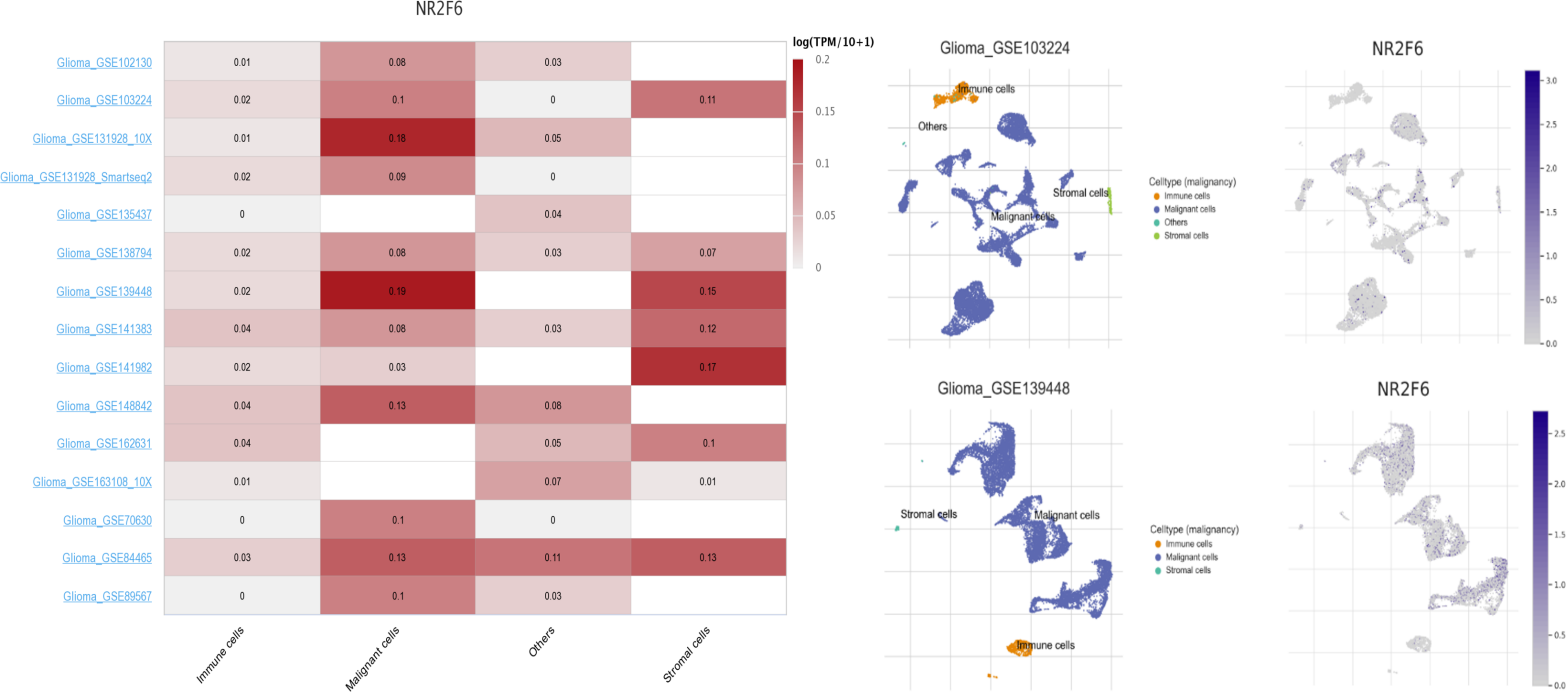
Expression levels of NR2F6 analysis by using TISCH in malignant cells, immune cells, and stromal cells. The lift figure shows the heatmap of NR2F6 expression using glioma single-cell sequencing datasets. The middle figure shows uniform manifold approximation and projection (UMAP) lots showing the glioma cell landscape. The right figure shows the UMAP plots illustrating the expression of NR2F6 clusters based on the GSE103224 and GSE139448 datasets.

To get a better understanding of the relationship between NR2F6 and the infiltrated cells, we analyzed the proportion of 22 immune cells between high and low NR2F6 expression groups in both TCGA and CGGA datasets using CIBERSORT software (45). We compared the analytical results of TCGA (**Figure 5A**) and CGGA (**Supplementary Figure S1A**), where we found a similar statistically significant difference in the distribution of T cells follicular helper, monocytes, NK cells resting, NK cells activated, M0 macrophages, M2 macrophages, and Mast cells activated. The increase in NR2F6 expression was associated with an increase in the proportion of T cells follicular helper, NK cells resting, M0 macrophages and M2 macrophages, and a decrease in the proportion of monocytes, NK cells activated and Mast cells activated. This suggests that NR2F6 has a remarkable influence on the infiltration level of immune cells. To further determine these findings, we next employed xCell (47) to analyze the correlation between NR2F6 expression and 46 immune and stromal cell populations. As shown in **Figure 5B** and **Supplementary Figure S1B**, in both TCGA and CGGA datasets, NR2F6 expression was significantly associated with immune score, stroma score, and microenvironment score. NR2F6 was remarkably positively correlated with the majority of stromal cells, as well as Treg, macrophages, M2 macrophage phenotype, and neutrophils, whereas B cells, eosinophils, plasma cells, Th1, Th2, CD8^+^ T cells, and CD8^+^ Tcm were negatively correlated with NR2F6 expression. These results strongly suggested that NR2F6 has an important influence on the infiltration of immune and stromal cells in the glioma microenvironment. The TME plays a pivotal role in tumor occurrence and development, which may accelerate tumor deterioration and affect the prognosis. We further used the TISCH database to analyze NR2F6 expression in TME-related cells. We found that NR2F6 was expressed in immune cells, malignant cells, and stromal cells. NR2F6 expression was the highest in malignant cells and stromal cells in the microenvironment of gliomas (**Figure 6**). These findings demonstrated that NR2F6 was closely related to TME in glioma.

### NR2F6 was associated with immunosuppressive properties

**Figure 7:**
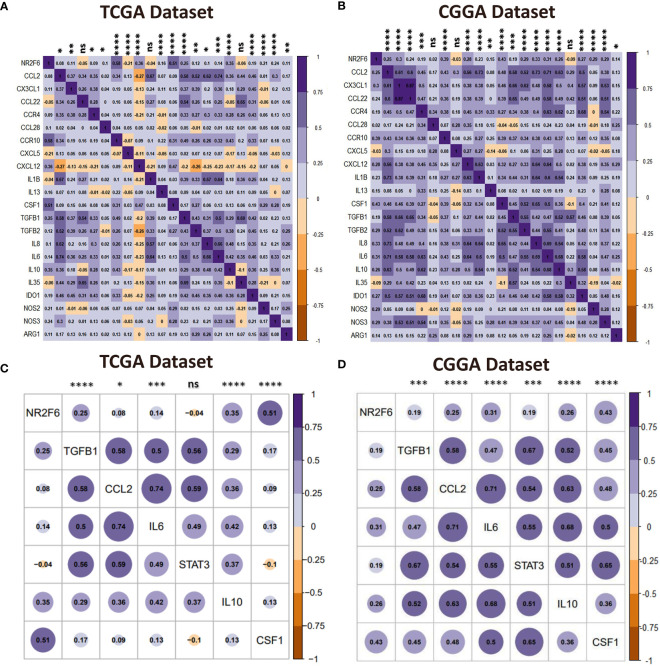
NR2F6 correlates with immunosuppressive activities. **(A, B)** Correlation of NR2F6 and immunosuppressive cells chemokines and immunosuppressive cytokines. The color intensity of the square is proportional to the correlation coefficients. Purple, positive correlation; Brown, negative correlation. **(C, D)** Correlation between NR2F6 and M2-promoting differentiation factors. Plot size and color depth show the intensity of the relationship, purple, positive correlation; brown, negative correlation; larger plot indicates a stronger correlation. *p<0.05; **p<0.01; ***p<0.001; ****p<0.0001; ns, not significant.

Immunosuppressive cells promote immune evasion by producing immunosuppressive cytokines in the tumor microenvironment, resulting in dysfunctional T cells. Our analysis of the tumor-infiltrating immune cells showed that NR2F6 was related to immunosuppressive cells such as regulatory T cells (Tregs), macrophages, and neutrophils. Therefore, we postulated that NR2F6 could be implicated in the immunosuppressive properties of glioma. To validate this, a correlation analysis was performed to determine the relationship between NR2F6 expression and critical immunosuppressive cytokines secreted by Tregs, tumor-associated macrophages, myeloid-derived suppressor cells, and tumor-associated neutrophils, as well as chemokines attracting these cells toward the tumor (54–56). We found that NR2F6 was significantly positively correlated with the majority of the chemokines and immunosuppressive cytokines (**Figures 7A, B**). TAMs are the most important immune cells in the glioma microenvironment, which skew towards an M2 phenotype and play a critical role in immunosuppression (57, 58). Interestingly, NR2F6 was positively associated with key factors driving M2 phenotype differentiation (54, 55) (**Figures 7C, D**). Taken together, these findings revealed that NR2F6 might play an important immunosuppressive role in glioma through recruiting and promoting immunosuppressive cells to secrete immune-inhibitory cytokines, as well as regulating M2 transformation.

### NR2F6 was correlated with other immune checkpoint markers in gliomas

**Figure 8:**
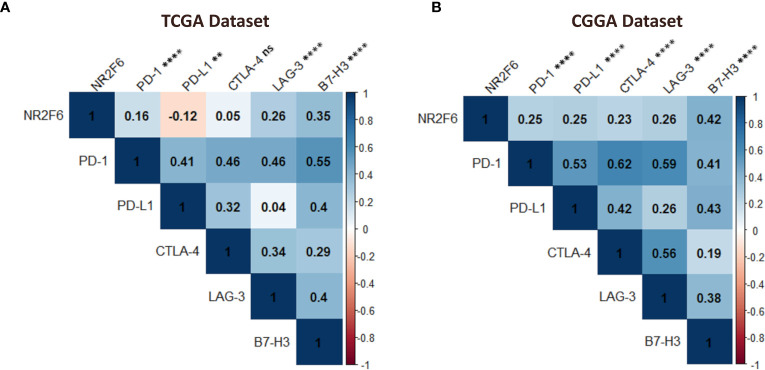
Association between NR2F6 and immune checkpoint markers in gliomas. the correlation of NR2F6 with other immune checkpoints including PD-1, PD-L1, CTLA-4, LAG-3, and B7-H3, based on TCGA **(A)** and CGGA **(B)** datasets. The color intensity of the square is proportional to the correlation coefficients. Blue, positive correlation; Red, negative correlation. **p<0.01; ****p<0.0001; ns, not significant.

Considering the increasing clinical benefits of targeting immune checkpoints as a combination therapy (59, 60), we enrolled several immune checkpoint molecules that have been examined in clinical trials or clinical situations into correlation analysis to assess their relationship with NR2F6 in glioma samples using both TCGA and CGGA datasets (61, 62). NR2F6 showed a positive association with PD-1, LAG-3, and B7-H3 in both datasets (**Figures 8A, B**), indicating the potential synergistic effects of NR2F6 with these checkpoint members.

## Discussion

Glioma, especially glioblastoma, is the most aggressive type of brain cancer and has a severe impact on patient health (63–65). Even with intensive therapies, the prognosis for glioblastoma patients is still dismal. This highlights the urgent need for new therapeutic approaches. In recent years, glioblastoma immunotherapy has gained increased interest, particularly in blocking immune checkpoints CTLA-4 and PD-1 (66). Glioma checkpoint inhibitor therapies have made continuous progress. However, a large proportion of patients do not respond to a single checkpoint inhibitor, therefore, it is necessary to explore novel immune checkpoints for additive or synergistic anti-tumor activities (66–68).

In this study, we comprehensively analyzed the expression pattern and related biological characteristics of the new immune checkpoint NR2F6 and its clinical significance in glioma. First, we proved that the expression of NR2F6 was significantly upregulated in the higher malignant pathological type of gliomas. Moreover, we also found that high expression of NR2F6 was highly enriched in the phenotype of known malignant molecule, the IDH wild-type state. All these results indicated that NR2F6 expression was associated with more malignant biologic process as other solid and hematologic malignancies (33, 36, 69, 70). Most likely, these malignant biologic behaviors have contributed to tumor recurrence and resistance to therapy. Revealing the mechanism of NR2F6 in glioma may be the key to triumphing over this fatal disease. Our findings also showed that a high expression level of NR2F6 in glioma was relevant to a worse prognosis in both the TCGA and CGGA databases. This was consistent with previously reported results (34, 69, 71), overexpression of NR2F6 predicted poor patient prognosis in various malignant tumors, such as ovarian cancer, early cervical cancer, and head and neck cancer.

Through an in-depth analysis of the biological function of NR2F6 in glioma, we found that NR2F6 was involved in extracellular matrix organization, angiogenesis, cell adhesion, and other biological processes related to glioma progression. Meanwhile, NR2F6 was involved in multiple immune-related functions and pathways, such as leukocyte migration, inflammatory response, T cell receptor signaling, and innate immune response. Moreover, the results of the tumor-infiltrating cells analysis showed that NR2F6 expression *significantly correlates with infiltrating stromal and immune cells in the glioma microenvironment.* More interestingly, we found that NR2F6 was expressed in both immune and malignant cells, as well as stromal cells in glioma patients using the TISCH database. Hence, the function of NR2F6 in glioma may be realized by the wide expression of NR2F6 in immune cells, glioma cells, and stromal cells. Previous studies have demonstrated that NR2F6 plays a dual function in immune cells and in tumor cells. In effector T lymphocytes, NR2F6 negatively controls TCR/CD28-mediated signal transduction by antagonizing the DNA accessibility of activation-induced NFAT/AP-1 transcription factors at critical cytokine gene loci such as IL2 and IFNg (28, 72). Recently, it has been shown that the genetic elimination of NR2F6 improves intratumoral CD4^+^ and CD8^+^ T-cell infiltration as well as effector functions by increasing the production of effector cytokines, resulting in strongly decelerated tumor growth in different spontaneous as well as transplantable mouse tumor models (39, 73). Besides its role in immune cells, NR2F6 is upregulated in various human cancer cells, such as cervical cancer (69), ovarian cancer (71, 74), colon carcinoma (36), leukemia (33, 35), lung cancer (70), breast cancer (37), and hepatocellular cancer (75), indicating that NR2F6 is involved in tumor promotion and progression.

Immune evasion and suppression are significant factors that prevent current immunotherapies from effectively fighting glioma. In tumors, immune-suppressive microenvironments promote the lesion’s growth and malignant properties while evading the body’s immune response (76–78). Thus, the discovery of potential immunosuppressive features of glioma has considerable importance. Here, we conducted correlation analysis with two different large datasets, and found that NR2F6 expression significantly positively correlates with chemokines that recruit immunosuppressive immune cells, such as Treg, macrophages, and neutrophils, as well as key immunosuppressive cytokines secreted by these cells. Macrophages are the main immune cells in the glioma microenvironment, which may constitute up to 50% of the total cellular composition and are usually polarized to M2 phenotype (79). In gliomas, M2 macrophages exhibit an immunosuppressive phenotype and are associated with poor prognosis (80). Our analysis found that NR2F6 was positively related to M2 differentiation factors, suggesting that it may contribute to a tumor microenvironment favorable for tumor growth through promoting the M2-polarization of tumor-associated macrophages. In addition, a positive association was observed between NR2F6 and multiple immune checkpoints. Studies have revealed that the upregulation of immune checkpoints such as PD-1, LAG-3, and B7-H3 in glioma aids tumor immune evasion, resulting in T cell dysfunction (81–86), which suggests that NR2F6 may promote glioma immune evasion through upregulation of immune checkpoint expression.

Collectively, we can speculate that, on the one hand, NR2F6 functions as a nonimmunological regulator: expressed in cancer, facilitating angiogenesis and tumor invasion. On the other hand, NR2F6 functions as an innate and adaptative immunity regulator: expressed in immune and stromal cells, promoting tumor escape from immune surveillance, resulting in poor outcomes for glioma patients. Mechanistically, NR2F6 might regulate the immunosuppressive microenvironment by recruiting immunosuppressive cells to produce immunosuppressive cytokines, regulating M2 polarization, and combining with other immune checkpoint inhibitory molecules.

In comparison with monotherapy, immunotherapies targeting combined checkpoint inhibitory pathways have demonstrated profound clinical benefits (87, 88). Specifically, combination treatment approaches were more effective and associated with significantly longer progression-free survival compared to checkpoint monotherapy (87). Recent studies have shown that Nr2f6-deficient mice exhibit tumor growth inhibition due to an enhanced anti-tumor immune response against both solid tumors and metastases, leading to overall survival benefit (39, 73). More importantly, the genetic ablation of NR2F6 in combination with the established blockade of surface checkpoints (PD-L1, CTLA-4) has a strong synergistic effect compared to the inhibition of immune checkpoints alone (39, 89, 90). Moreover, anti-tumor immune responses in the Nr2f6^−/−^ therapy groups did not show any signs of immune-related adverse events (irAE) (39). Thus, it can be inferred that the combination of NR2F6 blockade with other ICIs, such as PD-1, LAG-3, and B7-H3, may be an alternative treatment method for glioma patients.

The current study, which takes advantage of large population databases and systematic data analysis and shows promising transcriptional findings, provides novel insights regarding the involvement of the NR2F6 pathway in immune responses and cancer development. Therefore, it will greatly help with the development of more effective glioma treatment agents. However, future studies would help confirming the crucial role of NR2F6 in gliomas by examining NR2F6 expression at protein levels.

## Conclusion

In recent years, immunotherapy research for glioma has increased exponentially due to the success of immune checkpoint blockade in other cancers. However, current immunotherapies have been proven ineffective for most patients. This has raised our interest in finding novel alternative checkpoint target, which could result in enhanced therapeutic benefits for glioma treatment.

This is the first study exploring the expression pattern, clinical value, and biological function of the immune checkpoint NR2F6 in glioma. We found that high expression of NR2F6 was closely related to high tumor aggressiveness and predicted a poor outcome, and that NR2F6 expression was involved in glioma immunosuppression, tumor invasion, and progression in the inflammatory microenvironment of glioma. Our results highlighted NR2F6, which positively interacts with other checkpoint proteins in glioma, as a promising candidate for immunotherapy. Further investigation is required on the potential use of NR2F6 pathway inhibition in combination with multiple other immune checkpoint blockade for the treatment of glioma.

## Data availability statement

The original contributions presented in the study are included in the article/**Supplementary Material**. Further inquiries can be directed to the corresponding author.

## Ethics statement

The studies involving human participants were reviewed and approved by the Ethical Board of the Ibn Rochd University Hospital of Casablanca. Written informed consent to participate in this study was provided by the participants’ legal guardian/next of kin.

## Author contributions

HM conceived and designed the study; collected, analyzed and interpreted the data; wrote the manuscript and performed the experiment. ON, collected and analyzed the data. SA and AG, contributed to the experimental work. AL, analyzed the data and revised the manuscript. AB, analyzed the data, revised the manuscript and supervised the study. All authors contributed to the article and approved the submitted version. 

## Supplementary material

Supplementary Figure S1 Analysis of tumor immune and stromal cell infiltration relative to the NR2F6 level in the CGGA dataset. **(A)** Proportions of the 22 types of tumor-infiltrating immune cells (TIICs) in different NR2F6 groups. **(B)** Correlation between NR2F6 expression and xCell scores in gliomas. Each colored square within the figure illustrates the correlation between NR2F6 and immune, stromal, and microenvironment scores and 46 cell types. blue, positive correlation; red, negative correlation. * *p*<0.05; ** *p*<0.01; *** *p*<0.001; **** *p*<0.0001; ns, not significant.

Supplementary Table 1 Correlation between NR2F6 and clinicopathological characteristics in patients with gliomas in TCGA and CGGA.

Supplementary Table 2 Correlation between NR2F6 and clinicopathological characteristics in patients with gliomas in in-house cohort.

Supplementary Table 3 2159 DEGs in TCGA, including 1032 upregulated and 1127 downregulated genes.

Supplementary Table 4 772 DEGs in CGGA, including 375 upregulated and 397 downregulated genes.
