# Supplementary figures and images for: NR2F6, a new immune checkpoint that acts as a potential biomarker of immunosuppression and contributes to poor clinical outcome in human glioma
Source: Front Immunol. 2023 Jul 28;14:1139268. doi: 10.3389/fimmu.2023.1139268 (PMC10419227; doi:10.3389/fimmu.2023.1139268)

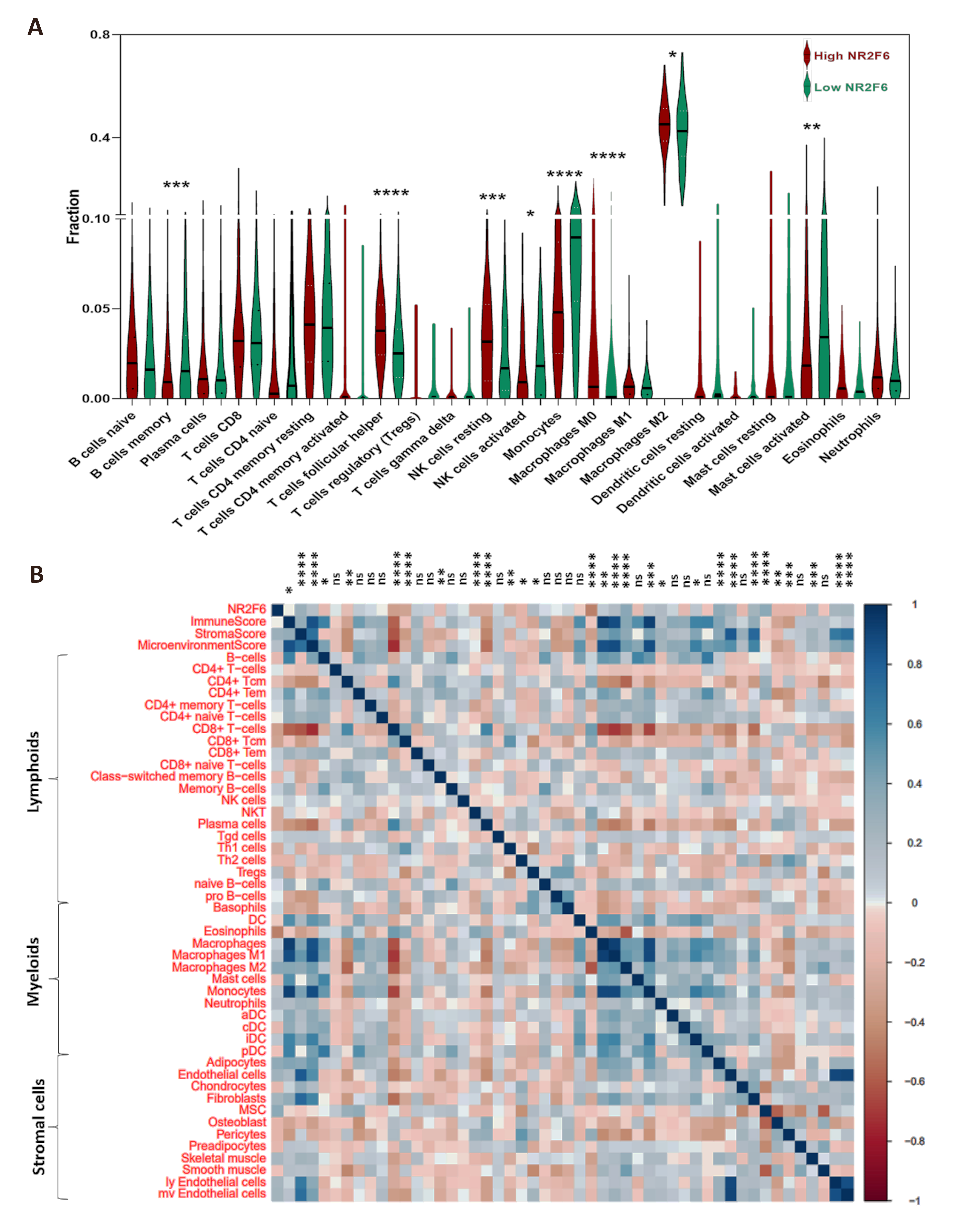

Supplement: Supplementary Figure S1 — Analysis of tumor immune and stromal cell infiltration relative to the NR2F6 level in the CGGA dataset. (A) Proportions of the 22 types of tumor-infiltrating immune cells (TIICs) in different NR2F6 groups. (B) Correlation between NR2F6 expression and xCell scores in gliomas. Each colored square within the figure illustrates the correlation between NR2F6 and immune, stromal, and microenvironment scores and 46 cell types. blue, positive correlation; red, negative correlation. * p<0.05; ** p<0.01; *** p<0.001; **** p<0.0001; ns, not significant. [file Image_1.jpeg]
