# Supplementary material for: NR2F6, a new immune checkpoint that acts as a potential biomarker of immunosuppression and contributes to poor clinical outcome in human glioma
Source: Front Immunol. 2023 Jul 28;14:1139268. doi: 10.3389/fimmu.2023.1139268 (PMC10419227; doi:10.3389/fimmu.2023.1139268)
Supplement: Supplementary Table 1 — Correlation between NR2F6 and clinicopathological characteristics in patients with gliomas in TCGA and CGGA. [file Table_1.docx]

| **TCGA Dataset**  **Variables cases (%) (n=663) *p* value** | **CGGA Dataset**  **Variables** C**ases (%) (n=325) *p* value** |
| --- | --- |
| **Sex**  Male 348 (56.32)  Female 270 (43.68) 0.6966  **Age**  ≤ 44 Years 314 (50.81)  > 44 Years 304 (49.19) <0.0001  **WHO grade**  Low grade (II) 248 (37.52)  High grade (III, IV) 413 (62.48) <0.0001  **IDH mutation status**  Yes 421 (68.01)  No 198 (31.99) <0.0001  **Histological type**  Glioblastoma 194 (29.31)  Astrocytoma 149 (22.51)  Oligoastrocytoma 130 (19.63)  Oligodendroglioma 189 (28.55) <0.0001 | **Sex**  Male 203 (62.46)  Female 122 (37.54) 0.6666  **Age**  ≤ 42 Years 167 (51.38)  > 42 Years 158 (48.62) 0.2531  **WHO grade**  Low grade (II) 103 (32.08)  High grade (III, IV) 218 (67.92) <0.0001  **IDH mutation status**  Yes 175 (54.01)  No 149 (45.99 <0.0001  **Histological type**  Glioblastoma 139 (43.31)  Anaplastic Astrocytoma 62 (19.31)  Anaplastic Oligoastrocytoma 12 (3.74)  Astrocytoma 56 (17.45)  Oligodendroglioma 52 (16.19) <0.0001 |
