# Supplementary material for: NR2F6, a new immune checkpoint that acts as a potential biomarker of immunosuppression and contributes to poor clinical outcome in human glioma
Source: Front Immunol. 2023 Jul 28;14:1139268. doi: 10.3389/fimmu.2023.1139268 (PMC10419227; doi:10.3389/fimmu.2023.1139268)
Supplement: Supplementary Table 2 — Correlation between NR2F6 and clinicopathological characteristics in patients with gliomas in in-house cohort. [file Table_2.docx]

| **In-house cohort**  **Variables cases (%) (n=60) *p* value** |
| --- |
| **Sex**  Male 37 (61.67)  Female 23 (38.33) 0.2046  **Age**  ≤ 34 Years 29 (48.33)  > 34 Years 31 (51.67) 0.0694  **WHO grade**  Low grade (I, II) 30 (50)  High grade (III, IV) 30 (50) <0.0001  **IDH mutation status**  Yes 7 (41.17)  No 10 (58.83) 0.0046  **Histological type**  Glioblastoma 22 (36.67)  Astrocytoma 24 (40)  Oligoastrocytoma 3 (5)  Ependymoma 10 (16.67)  Xantoastrocytoma 1 (1.66) <0.0001 |
