# Supplementary material for: NR2F6, a new immune checkpoint that acts as a potential biomarker of immunosuppression and contributes to poor clinical outcome in human glioma
Source: Front Immunol. 2023 Jul 28;14:1139268. doi: 10.3389/fimmu.2023.1139268 (PMC10419227; doi:10.3389/fimmu.2023.1139268)
Supplement: Supplementary Table 3 — 2159 DEGs in TCGA, including 1032 upregulated and 1127 downregulated genes. [file Table_3.docx]

| **Expression** | **Genes symbol** | **logFC** | **adj.P.Val** |
| --- | --- | --- | --- |
| **Upregulated** | SSBP4 | 0.66589794 | 1.11305E-53 |
|  | CDC37 | 0.414860774 | 9.66813E-53 |
|  | MAP2K2 | 0.437109791 | 1.03195E-51 |
|  | NCLN | 0.510237464 | 9.41508E-51 |
|  | CSNK1G2 | 0.488286565 | 1.49554E-50 |
|  | STK11 | 0.453796213 | 3.91698E-49 |
|  | MRPL4 | 0.468806815 | 3.9181E-49 |
|  | DAPK3 | 0.523755616 | 4.72621E-49 |
|  | ZNF579 | 0.809787166 | 4.07187E-48 |
|  | ZNF628 | 0.668768144 | 4.35292E-48 |
|  | FAM108A1 | 0.575244882 | 2.21197E-47 |
|  | RNF126 | 0.47406195 | 1.13517E-46 |
|  | CTU1 | 0.762693189 | 5.26226E-46 |
|  | ZNF771 | 0.758924799 | 8.99264E-45 |
|  | CHST12 | 0.591369208 | 9.5542E-45 |
|  | C19orf20 | 1.140746017 | 1.29926E-44 |
|  | PRR7 | 1.046348298 | 1.29926E-44 |
|  | C19orf60 | 0.735566296 | 2.14899E-44 |
|  | SCAND1 | 0.632689506 | 1.81291E-43 |
|  | C19orf28 | 0.502887634 | 8.25223E-43 |
|  | ZNF787 | 0.559673374 | 8.39895E-43 |
|  | ATP5D | 0.622167665 | 1.26568E-42 |
|  | CDC34 | 0.516708649 | 1.42392E-42 |
|  | CCDC124 | 0.466666094 | 1.90105E-42 |
|  | CCDC85B | 0.965266557 | 7.26227E-42 |
|  | CCR10 | 1.000045965 | 5.30623E-41 |
|  | MBD3 | 0.488372269 | 9.72747E-41 |
|  | FBXW5 | 0.426514457 | 1.40739E-40 |
|  | CLPP | 0.550787416 | 1.72901E-40 |
|  | TMEM161A | 0.472394581 | 4.62276E-40 |
|  | LRFN3 | 0.526087719 | 4.89644E-40 |
|  | CLEC11A | 0.857193724 | 6.25092E-40 |
|  | ZNF837 | 0.869808869 | 6.76232E-40 |
|  | PPDPF | 0.697324618 | 8.60201E-40 |
|  | IRF2BP1 | 0.476182127 | 2.50512E-39 |
|  | C20orf20 | 0.525037276 | 5.17751E-39 |
|  | SLC39A3 | 0.536823499 | 5.74229E-39 |
|  | MED16 | 0.430569776 | 6.66918E-39 |
|  | FAM128B | 0.586858372 | 9.04257E-39 |
|  | PUS1 | 0.470800363 | 1.06547E-38 |
|  | EPN1 | 0.483521494 | 1.11846E-38 |
|  | CD320 | 0.583849604 | 6.60805E-38 |
|  | C19orf6 | 0.504200345 | 7.2039E-38 |
|  | IER5L | 1.223259113 | 8.32181E-38 |
|  | TMEM160 | 0.826864808 | 8.6535E-38 |
|  | ADAT3 | 1.039225906 | 1.58596E-37 |
|  | ALKBH6 | 0.570140975 | 1.59644E-37 |
|  | GLTPD1 | 0.483159243 | 1.59644E-37 |
|  | TMUB1 | 0.597858484 | 1.79026E-37 |
|  | MFSD10 | 0.619395764 | 2.72507E-37 |
|  | RPS15 | 0.596496176 | 7.22739E-37 |
|  | C19orf43 | 0.493675462 | 7.32911E-37 |
|  | ARHGDIA | 0.4155264 | 1.14051E-36 |
|  | CARM1 | 0.403467425 | 1.29211E-36 |
|  | HSPBP1 | 0.525867999 | 3.24677E-36 |
|  | C19orf24 | 0.629558458 | 4.23861E-36 |
|  | BSG | 0.420356634 | 6.09708E-36 |
|  | PGLS | 0.548417604 | 1.04559E-35 |
|  | RPUSD1 | 0.413667539 | 1.61531E-35 |
|  | KLF16 | 0.565554386 | 3.65256E-35 |
|  | MEX3D | 0.643735555 | 4.13937E-35 |
|  | GPR172A | 0.49759606 | 5.1983E-35 |
|  | MIF | 0.831029639 | 8.60665E-35 |
|  | C7orf47 | 0.691402452 | 1.23609E-34 |
|  | GIPC1 | 0.415199952 | 4.09102E-34 |
|  | NAT14 | 0.626330802 | 6.8645E-34 |
|  | DDRGK1 | 0.40751898 | 9.90015E-34 |
|  | QPCTL | 0.632312515 | 1.52784E-33 |
|  | CRIP2 | 0.555179047 | 1.58737E-33 |
|  | SOLH | 0.49383596 | 2.02702E-33 |
|  | TRAPPC5 | 0.573729373 | 2.36107E-33 |
|  | C16orf13 | 0.476026631 | 2.36107E-33 |
|  | MGAT4B | 0.458896216 | 2.75702E-33 |
|  | GET4 | 0.408152946 | 2.75702E-33 |
|  | APBA3 | 0.500924503 | 2.97763E-33 |
|  | ZBTB45 | 0.413701251 | 4.85174E-33 |
|  | DCAF15 | 0.408206892 | 6.79277E-33 |
|  | LSM4 | 0.50616504 | 9.13148E-33 |
|  | ATG4D | 0.436227377 | 1.06263E-32 |
|  | RABEP2 | 0.476325788 | 1.19542E-32 |
|  | SPATA2L | 0.533645381 | 1.56123E-32 |
|  | ZFPM1 | 0.706108595 | 1.74244E-32 |
|  | RBM42 | 0.512467719 | 2.48527E-32 |
|  | TIMM13 | 0.433107106 | 5.9004E-32 |
|  | MRPS26 | 0.423226878 | 6.98867E-32 |
|  | WDR18 | 0.512031757 | 7.05663E-32 |
|  | ZNF784 | 0.532093476 | 7.86048E-32 |
|  | B3GALT6 | 0.49792527 | 8.95098E-32 |
|  | FAM176B | 1.106321905 | 9.82695E-32 |
|  | TMEM86B | 0.618156158 | 1.4781E-31 |
|  | AES | 0.520751126 | 2.16738E-31 |
|  | C20orf27 | 0.413178131 | 2.23959E-31 |
|  | DUS1L | 0.424424167 | 2.41206E-31 |
|  | LENG9 | 0.892242141 | 2.78674E-31 |
|  | GTPBP3 | 0.472335923 | 4.05895E-31 |
|  | TMEM93 | 0.430321931 | 4.08832E-31 |
|  | PUSL1 | 0.593917538 | 4.26247E-31 |
|  | GIYD2 | 0.6169704 | 5.1513E-31 |
|  | ATP6V0C | 0.429154552 | 5.94489E-31 |
|  | SAC3D1 | 0.544301766 | 6.62909E-31 |
|  | FAM173A | 0.643765456 | 1.00994E-30 |
|  | MRPL12 | 0.454965857 | 1.21498E-30 |
|  | C1orf159 | 0.564232239 | 2.1645E-30 |
|  | ZNF580 | 0.551930829 | 4.58098E-30 |
|  | PIN1 | 0.439283711 | 8.82072E-30 |
|  | GNB2 | 0.458728236 | 1.24589E-29 |
|  | TRADD | 0.670755844 | 1.63003E-29 |
|  | KIAA0415 | 0.408634716 | 1.76206E-29 |
|  | UBE2S | 0.792858928 | 1.81082E-29 |
|  | LOC407835 | 0.606855806 | 1.81082E-29 |
|  | IMPDH1 | 0.482245079 | 2.43396E-29 |
|  | ZNF593 | 0.658114186 | 2.71694E-29 |
|  | CCDC130 | 0.449238515 | 3.7763E-29 |
|  | ZNF414 | 0.497123989 | 4.62847E-29 |
|  | C19orf10 | 0.540728791 | 5.43258E-29 |
|  | LRWD1 | 0.41677509 | 6.08092E-29 |
|  | FASTK | 0.419342604 | 6.88958E-29 |
|  | OGFR | 0.403657132 | 9.69676E-29 |
|  | LSM7 | 0.591384322 | 1.10954E-28 |
|  | FBXL8 | 0.823667181 | 1.46169E-28 |
|  | AURKAIP1 | 0.464136091 | 1.49221E-28 |
|  | MRPS24 | 0.442244724 | 2.96474E-28 |
|  | MRPS12 | 0.635960887 | 3.0579E-28 |
|  | C1orf86 | 0.603205274 | 3.18967E-28 |
|  | CCDC106 | 0.482309955 | 3.42469E-28 |
|  | SCAF1 | 0.415691163 | 3.47224E-28 |
|  | ZNF444 | 0.488668253 | 3.67848E-28 |
|  | SRM | 0.50591778 | 5.89299E-28 |
|  | ZNF358 | 0.455399389 | 6.20023E-28 |
|  | COX8A | 0.410485812 | 7.6214E-28 |
|  | ITPA | 0.433174292 | 8.05258E-28 |
|  | POLR2I | 0.545386182 | 9.28045E-28 |
|  | C7orf30 | 0.467308099 | 1.34383E-27 |
|  | C7orf27 | 0.448278166 | 1.62505E-27 |
|  | BCAT2 | 0.567063338 | 1.79665E-27 |
|  | SIRT6 | 0.407493873 | 2.4576E-27 |
|  | NDUFS8 | 0.419631489 | 2.5084E-27 |
|  | TNFRSF6B | 0.825202272 | 3.61217E-27 |
|  | MOSPD3 | 0.444200293 | 4.38062E-27 |
|  | SNAPC2 | 0.565568926 | 4.38522E-27 |
|  | PRKCDBP | 1.144297258 | 5.36632E-27 |
|  | QTRT1 | 0.53095977 | 5.36632E-27 |
|  | PRR24 | 0.865981024 | 7.2402E-27 |
|  | ALDH16A1 | 0.540745693 | 8.1474E-27 |
|  | PNKP | 0.462631486 | 8.81719E-27 |
|  | CCM2 | 0.40393705 | 9.41772E-27 |
|  | MESDC1 | 0.469291222 | 1.16086E-26 |
|  | GGN | 0.95761252 | 1.3646E-26 |
|  | HIC1 | 0.848409766 | 1.41534E-26 |
|  | INO80B | 0.552923443 | 2.24438E-26 |
|  | SUV420H2 | 0.531968573 | 2.39829E-26 |
|  | ZNF653 | 0.42680015 | 2.39829E-26 |
|  | PSMG3 | 0.549007952 | 2.48452E-26 |
|  | ZNF524 | 0.504322405 | 3.13244E-26 |
|  | SLC2A4RG | 0.691124741 | 3.23609E-26 |
|  | PTP4A3 | 0.708880738 | 4.51693E-26 |
|  | CHPF | 0.579647831 | 7.83975E-26 |
|  | CC2D1A | 0.417861835 | 9.29481E-26 |
|  | FBXW9 | 0.498354969 | 1.16066E-25 |
|  | NAA10 | 0.409085347 | 1.17791E-25 |
|  | UBE2J2 | 0.413998813 | 1.49195E-25 |
|  | NOC4L | 0.405889935 | 1.86383E-25 |
|  | POLR2H | 0.404087985 | 2.47563E-25 |
|  | TMEM8A | 0.454707108 | 2.6476E-25 |
|  | VKORC1 | 0.595090882 | 3.01738E-25 |
|  | RABAC1 | 0.519510182 | 3.03944E-25 |
|  | DPM2 | 0.409014003 | 3.11404E-25 |
|  | JUND | 0.460749006 | 3.81252E-25 |
|  | FDX1L | 0.423077462 | 5.95506E-25 |
|  | PDLIM7 | 0.820134078 | 6.74735E-25 |
|  | MIB2 | 0.462585443 | 7.67486E-25 |
|  | ISYNA1 | 0.667304487 | 8.34712E-25 |
|  | NR2C2AP | 0.471907374 | 8.60403E-25 |
|  | MZF1 | 0.547803606 | 9.91581E-25 |
|  | TRIM28 | 0.436911443 | 1.06664E-24 |
|  | NDUFB7 | 0.460792125 | 1.20084E-24 |
|  | LEPRE1 | 0.656842079 | 1.33857E-24 |
|  | YIF1B | 0.416170255 | 1.34838E-24 |
|  | MRPL34 | 0.45420958 | 1.35171E-24 |
|  | GAPDH | 0.440549376 | 1.37638E-24 |
|  | ANKRD9 | 0.804149148 | 1.94077E-24 |
|  | RCN3 | 0.840458448 | 2.11788E-24 |
|  | MYPOP | 0.505620484 | 2.85664E-24 |
|  | SDF4 | 0.452980295 | 3.26826E-24 |
|  | ALG3 | 0.435445255 | 4.01924E-24 |
|  | IFRD2 | 0.434009049 | 4.08681E-24 |
|  | UCKL1 | 0.418483192 | 4.33287E-24 |
|  | JOSD2 | 0.578217979 | 5.68757E-24 |
|  | LRFN4 | 0.520561552 | 5.79071E-24 |
|  | PSMG4 | 0.443500907 | 6.77942E-24 |
|  | SNHG9 | 0.723491957 | 6.85396E-24 |
|  | UBE2M | 0.400956257 | 9.92653E-24 |
|  | TIMM16 | 0.492339807 | 1.04367E-23 |
|  | TRABD | 0.525171911 | 1.08493E-23 |
|  | CCDC107 | 0.442342017 | 1.12712E-23 |
|  | ZNF581 | 0.509742916 | 1.35818E-23 |
|  | COX5B | 0.449259048 | 1.39362E-23 |
|  | TP53I13 | 0.639945698 | 1.4024E-23 |
|  | DUSP15 | 0.529937644 | 1.43829E-23 |
|  | PAK4 | 0.401426353 | 1.43998E-23 |
|  | C4orf48 | 0.829193271 | 1.80572E-23 |
|  | DHX34 | 0.433893896 | 1.81832E-23 |
|  | H2AFX | 0.597916161 | 2.04671E-23 |
|  | F8A1 | 0.456098037 | 2.8215E-23 |
|  | BCAM | 0.638277011 | 3.52729E-23 |
|  | UNC45A | 0.410447017 | 3.55955E-23 |
|  | WTIP | 0.719939446 | 3.94325E-23 |
|  | ALKBH7 | 0.445418056 | 4.201E-23 |
|  | PLOD3 | 0.561056724 | 5.16443E-23 |
|  | MICALL2 | 0.822680885 | 5.37597E-23 |
|  | LIN37 | 0.401566138 | 6.08974E-23 |
|  | THBS3 | 0.601976312 | 8.48769E-23 |
|  | ZNF428 | 0.460104917 | 8.73025E-23 |
|  | HM13 | 0.417002024 | 9.19566E-23 |
|  | PDF | 0.482889932 | 9.45483E-23 |
|  | MBOAT7 | 0.479614552 | 1.00772E-22 |
|  | SPPL2B | 0.49842016 | 1.08089E-22 |
|  | UQCR11 | 0.426551179 | 1.082E-22 |
|  | RPL28 | 0.491641679 | 1.0984E-22 |
|  | C11orf83 | 0.465788775 | 1.59651E-22 |
|  | RPL36 | 0.470498874 | 1.62646E-22 |
|  | DHRS13 | 0.60863137 | 2.29648E-22 |
|  | SOX18 | 0.83951217 | 2.70005E-22 |
|  | DCI | 0.427465084 | 2.74584E-22 |
|  | FBXL6 | 0.497859365 | 2.82651E-22 |
|  | FAM20C | 0.818138194 | 3.17484E-22 |
|  | CPSF4 | 0.424777801 | 3.52157E-22 |
|  | FAM195A | 0.52287901 | 3.91045E-22 |
|  | NPEPL1 | 0.690185264 | 4.08024E-22 |
|  | EGLN2 | 0.414219125 | 5.07463E-22 |
|  | POLD1 | 0.561892351 | 5.20999E-22 |
|  | PLSCR3 | 0.403249921 | 5.22665E-22 |
|  | BCL3 | 0.883797734 | 5.53619E-22 |
|  | COMTD1 | 0.65039182 | 6.51153E-22 |
|  | CAPNS1 | 0.455793415 | 6.63106E-22 |
|  | GADD45GIP1 | 0.427688272 | 7.21034E-22 |
|  | PPP1R16A | 0.417493813 | 7.45354E-22 |
|  | GLI4 | 0.524946358 | 7.66933E-22 |
|  | FAM100B | 0.517412889 | 7.72749E-22 |
|  | SDHAF1 | 0.437191217 | 1.11557E-21 |
|  | FAM128A | 0.490074795 | 1.17048E-21 |
|  | MXD3 | 0.723990679 | 1.30489E-21 |
|  | NOSIP | 0.443626973 | 1.71261E-21 |
|  | METRNL | 0.680180278 | 2.47733E-21 |
|  | GMDS | 0.424231393 | 2.82952E-21 |
|  | MAP2K3 | 0.545932419 | 3.45364E-21 |
|  | LEPREL2 | 0.676491806 | 4.07459E-21 |
|  | NPFF | 0.783057711 | 4.11311E-21 |
|  | C11orf31 | 0.418722769 | 5.22469E-21 |
|  | LRP3 | 0.430364699 | 5.25055E-21 |
|  | NUCB1 | 0.435034559 | 5.90599E-21 |
|  | C7orf50 | 0.455387509 | 6.03352E-21 |
|  | TMEM44 | 0.479490715 | 6.04832E-21 |
|  | AP2S1 | 0.529230171 | 6.24263E-21 |
|  | GLTSCR1 | 0.44159363 | 6.39136E-21 |
|  | ORAI1 | 0.508411914 | 6.70964E-21 |
|  | SDF2L1 | 0.562094192 | 6.90597E-21 |
|  | SCARF2 | 0.623765387 | 7.73E-21 |
|  | GPC1 | 0.595447514 | 8.02581E-21 |
|  | TARBP2 | 0.412668903 | 8.29633E-21 |
|  | TNFRSF25 | 0.850983713 | 8.52298E-21 |
|  | BBC3 | 0.524480426 | 9.81027E-21 |
|  | TPI1 | 0.421595181 | 1.21563E-20 |
|  | STAP2 | 0.650949663 | 1.36007E-20 |
|  | ZGLP1 | 0.670865172 | 1.36893E-20 |
|  | ADCK5 | 0.512396113 | 1.4739E-20 |
|  | PIM3 | 0.452135343 | 2.24519E-20 |
|  | HOOK2 | 0.511178768 | 2.28426E-20 |
|  | MRPL23 | 0.426323328 | 2.31698E-20 |
|  | MESP1 | 0.575643673 | 3.00785E-20 |
|  | DDX39 | 0.483892964 | 3.21751E-20 |
|  | C11orf80 | 0.510766334 | 3.24101E-20 |
|  | SRRM5 | 0.550607783 | 3.38458E-20 |
|  | IRF3 | 0.456175334 | 3.96313E-20 |
|  | ZYX | 0.678346361 | 4.06465E-20 |
|  | SBNO2 | 0.529106375 | 4.18723E-20 |
|  | GLT25D1 | 0.533302396 | 5.30641E-20 |
|  | P4HB | 0.424268955 | 5.53677E-20 |
|  | UQCRHL | 0.482809093 | 6.17818E-20 |
|  | FOXS1 | 0.832075037 | 7.23635E-20 |
|  | IDUA | 0.615708953 | 8.00933E-20 |
|  | SNRPB | 0.472821831 | 8.421E-20 |
|  | LMNB2 | 0.475584715 | 9.87903E-20 |
|  | NUDT19 | 0.412641199 | 1.06953E-19 |
|  | ICAM3 | 0.4323818 | 1.08693E-19 |
|  | HMG20B | 0.4366885 | 1.24631E-19 |
|  | MGC70857 | 0.479504674 | 1.30067E-19 |
|  | BAX | 0.452241821 | 1.42338E-19 |
|  | UFSP1 | 0.581033772 | 1.47304E-19 |
|  | ISOC2 | 0.435931139 | 2.03014E-19 |
|  | FSTL3 | 0.589680562 | 2.14354E-19 |
|  | LYPLA2P1 | 0.467146844 | 2.30549E-19 |
|  | SLC27A3 | 0.82555757 | 2.54083E-19 |
|  | DNAJC4 | 0.401243832 | 2.94279E-19 |
|  | ROMO1 | 0.523954826 | 3.07134E-19 |
|  | CD151 | 0.698216561 | 3.31026E-19 |
|  | PHPT1 | 0.422023387 | 3.61868E-19 |
|  | CHPF2 | 0.456756391 | 3.89936E-19 |
|  | DDTL | 0.509313713 | 3.92695E-19 |
|  | COX6B1 | 0.41608455 | 3.9486E-19 |
|  | OCEL1 | 0.416016956 | 4.36601E-19 |
|  | WDR34 | 0.509427544 | 5.50624E-19 |
|  | C20orf135 | 0.501240724 | 6.01838E-19 |
|  | TMEM147 | 0.458326463 | 6.42782E-19 |
|  | TSPAN4 | 0.544885968 | 7.7531E-19 |
|  | CENPP | 0.552601508 | 7.95775E-19 |
|  | CDK10 | 0.459391562 | 9.07372E-19 |
|  | LSMD1 | 0.407631052 | 9.43395E-19 |
|  | CFP | 0.613704939 | 1.32331E-18 |
|  | SLC25A10 | 0.441936843 | 1.39815E-18 |
|  | SNHG3 | 0.649408357 | 1.48515E-18 |
|  | ZNF497 | 0.418329266 | 1.65114E-18 |
|  | GUSB | 0.562847531 | 1.79815E-18 |
|  | SYDE1 | 0.606092365 | 1.81169E-18 |
|  | CTXN1 | 0.678325988 | 2.01957E-18 |
|  | DOHH | 0.474605769 | 2.08918E-18 |
|  | CDC25B | 0.42015306 | 2.11089E-18 |
|  | SPON2 | 1.332752876 | 2.14213E-18 |
|  | TGFB1I1 | 0.747072804 | 2.17001E-18 |
|  | UBE2MP1 | 0.599051904 | 2.17657E-18 |
|  | HAGHL | 0.643673149 | 2.25955E-18 |
|  | SNHG12 | 0.568675697 | 2.3936E-18 |
|  | NAB2 | 0.42413167 | 2.39663E-18 |
|  | C8orf73 | 0.585272622 | 2.43366E-18 |
|  | SIVA1 | 0.425859478 | 2.56145E-18 |
|  | GNB1L | 0.40620671 | 2.56145E-18 |
|  | GEMIN7 | 0.449554033 | 2.8418E-18 |
|  | TRPM4 | 0.479532986 | 3.05087E-18 |
|  | PRMT1 | 0.414938891 | 3.16182E-18 |
|  | KDELR1 | 0.489551704 | 3.23993E-18 |
|  | MFSD3 | 0.454161821 | 3.5805E-18 |
|  | GPI | 0.442700135 | 3.70007E-18 |
|  | NXT1 | 0.556043897 | 4.01748E-18 |
|  | LOC388796 | 0.645167005 | 4.94346E-18 |
|  | SHFM1 | 0.423462574 | 5.14073E-18 |
|  | PFN1 | 0.43742824 | 8.67917E-18 |
|  | SC65 | 0.477319244 | 9.23675E-18 |
|  | NME2P1 | 0.666830088 | 1.00388E-17 |
|  | POLR2L | 0.521192151 | 1.11085E-17 |
|  | CRIP1 | 0.899511217 | 1.11945E-17 |
|  | PBX4 | 0.683830815 | 1.17874E-17 |
|  | PHLDB3 | 0.418651098 | 1.19378E-17 |
|  | SNRPD2 | 0.51548134 | 1.20222E-17 |
|  | WDR54 | 0.459729387 | 1.31182E-17 |
|  | SLC26A6 | 0.467394873 | 1.32058E-17 |
|  | FAM43A | 0.590478652 | 1.40817E-17 |
|  | ZNF503 | 0.418616866 | 1.49474E-17 |
|  | NME3 | 0.524480933 | 1.51142E-17 |
|  | NRM | 0.641809292 | 1.617E-17 |
|  | KRT10 | 0.421572864 | 1.74829E-17 |
|  | LOC440957 | 0.507771246 | 2.1963E-17 |
|  | CCDC61 | 0.401387777 | 2.38039E-17 |
|  | UQCRQ | 0.433154219 | 2.4085E-17 |
|  | MBLAC1 | 0.44023656 | 2.462E-17 |
|  | PCOLCE | 1.170230197 | 2.54553E-17 |
|  | DPM3 | 0.454107636 | 2.62379E-17 |
|  | C19orf48 | 0.532826317 | 2.85157E-17 |
|  | C19orf76 | 0.55378643 | 3.00128E-17 |
|  | RP9 | 0.404121021 | 3.00128E-17 |
|  | TMEM91 | 0.587281175 | 3.98341E-17 |
|  | LRFN1 | 0.489973193 | 4.11806E-17 |
|  | PTRH1 | 0.419507576 | 4.37308E-17 |
|  | HMGA1 | 0.518734726 | 4.65027E-17 |
|  | PTMS | 0.435875073 | 4.864E-17 |
|  | YDJC | 0.677308178 | 5.53617E-17 |
|  | BRI3 | 0.426014751 | 5.5923E-17 |
|  | C6orf26 | 0.631497277 | 5.6476E-17 |
|  | TPM2 | 0.700420889 | 8.15608E-17 |
|  | LMNA | 0.52612562 | 8.61928E-17 |
|  | DEDD2 | 0.54924121 | 9.01274E-17 |
|  | SLC22A18 | 0.824553278 | 1.04073E-16 |
|  | RECQL4 | 0.63688285 | 1.0458E-16 |
|  | NPDC1 | 0.461703559 | 1.06818E-16 |
|  | RASSF7 | 0.539707714 | 1.09903E-16 |
|  | PMM2 | 0.400950773 | 1.14801E-16 |
|  | NCRNA00116 | 0.492007411 | 1.22872E-16 |
|  | HAPLN3 | 0.686971525 | 1.23158E-16 |
|  | C16orf59 | 0.648641238 | 1.48376E-16 |
|  | C16orf93 | 0.499996901 | 1.55211E-16 |
|  | PLOD1 | 0.519891221 | 2.0057E-16 |
|  | SEMA3F | 0.914112769 | 2.36615E-16 |
|  | IL4I1 | 0.755113752 | 2.37197E-16 |
|  | H1FX | 0.403644503 | 2.44937E-16 |
|  | NDUFA4L2 | 0.714391814 | 2.65086E-16 |
|  | TBXA2R | 0.619979426 | 2.90216E-16 |
|  | BCL2L12 | 0.774525362 | 3.41724E-16 |
|  | DTYMK | 0.546317229 | 3.82696E-16 |
|  | SLC25A19 | 0.405252249 | 4.58489E-16 |
|  | FBL | 0.416623102 | 5.36764E-16 |
|  | PRKD2 | 0.419926276 | 5.60471E-16 |
|  | RIN1 | 0.965334238 | 5.93453E-16 |
|  | DMPK | 0.483669016 | 6.47069E-16 |
|  | SNRNP70 | 0.491003013 | 6.58959E-16 |
|  | FAM158A | 0.404140173 | 8.32664E-16 |
|  | IL17RE | 0.504124225 | 8.50802E-16 |
|  | COL18A1 | 0.623165548 | 8.76353E-16 |
|  | TNFRSF14 | 0.655018844 | 8.79492E-16 |
|  | LOC606724 | 0.615683197 | 8.85219E-16 |
|  | SLC25A29 | 0.420194709 | 9.49425E-16 |
|  | C2orf81 | 0.480896484 | 1.04059E-15 |
|  | IGFBP2 | 1.656345969 | 1.10999E-15 |
|  | BOLA3 | 0.400382741 | 1.19873E-15 |
|  | EIF4EBP1 | 0.605119037 | 1.37012E-15 |
|  | ERF | 0.415946159 | 1.43908E-15 |
|  | CHAF1A | 0.507367783 | 1.47119E-15 |
|  | SULT1A3 | 0.515163934 | 1.55023E-15 |
|  | PVRL2 | 0.454137342 | 1.63954E-15 |
|  | C6orf226 | 0.411042166 | 1.65547E-15 |
|  | SOD3 | 1.034568914 | 1.7707E-15 |
|  | C7orf53 | 0.536561918 | 2.05274E-15 |
|  | LRRC45 | 0.417021576 | 2.06602E-15 |
|  | MYO19 | 0.401819061 | 2.09779E-15 |
|  | PKN3 | 0.490623114 | 2.27593E-15 |
|  | CD248 | 1.152771736 | 2.30307E-15 |
|  | RNASEH2A | 0.537928385 | 2.55752E-15 |
|  | NME2 | 0.428512325 | 2.89084E-15 |
|  | AGRN | 0.455579969 | 3.2044E-15 |
|  | EFNB1 | 0.404457012 | 3.48298E-15 |
|  | PPIA | 0.40566434 | 3.62585E-15 |
|  | NUMBL | 0.468759736 | 3.71813E-15 |
|  | REEP4 | 0.488214948 | 3.91298E-15 |
|  | ATP5J2 | 0.435571286 | 3.94374E-15 |
|  | MANF | 0.42985747 | 4.00312E-15 |
|  | PABPC1L | 0.861648237 | 4.07219E-15 |
|  | NENF | 0.404581192 | 4.43905E-15 |
|  | NSUN5P1 | 0.614358962 | 4.55608E-15 |
|  | CCDC84 | 0.471007986 | 4.71596E-15 |
|  | ECHDC2 | 0.836243993 | 5.51958E-15 |
|  | HSPB1 | 0.775804627 | 6.36894E-15 |
|  | PRSS53 | 0.536844629 | 6.62652E-15 |
|  | SDC1 | 0.994678542 | 7.02204E-15 |
|  | GALK1 | 0.408450546 | 7.11009E-15 |
|  | EXOC3L | 0.589969402 | 7.399E-15 |
|  | HAUS8 | 0.474083649 | 8.28485E-15 |
|  | ARAP3 | 0.786671235 | 8.61216E-15 |
|  | CCNF | 0.513051496 | 9.47212E-15 |
|  | ATAD3B | 0.504864304 | 1.15198E-14 |
|  | NDUFA3 | 0.437848736 | 1.18181E-14 |
|  | SUSD2 | 0.797268895 | 1.30547E-14 |
|  | CBR3 | 0.535225499 | 1.42036E-14 |
|  | GRAP | 0.483278187 | 1.49662E-14 |
|  | TMSB10 | 0.653446058 | 1.55211E-14 |
|  | AGPAT2 | 0.535366007 | 1.56379E-14 |
|  | ECM1 | 0.570524765 | 1.63537E-14 |
|  | RPS19 | 0.446846857 | 1.64556E-14 |
|  | VASN | 1.146837293 | 1.66778E-14 |
|  | CARHSP1 | 0.458319629 | 1.79664E-14 |
|  | SERPINH1 | 0.89521995 | 1.8905E-14 |
|  | TMEM158 | 0.953672252 | 1.99993E-14 |
|  | DNLZ | 0.412153503 | 2.07714E-14 |
|  | ACYP1 | 0.466666404 | 2.08651E-14 |
|  | LIG1 | 0.424483591 | 2.64705E-14 |
|  | SIX5 | 0.618279131 | 2.7216E-14 |
|  | CEBPD | 0.862103156 | 4.10144E-14 |
|  | HOMER3 | 0.401620585 | 4.13283E-14 |
|  | BGN | 0.836956139 | 4.31977E-14 |
|  | E2F1 | 0.714897916 | 5.26932E-14 |
|  | UPP1 | 0.817488795 | 6.42845E-14 |
|  | LSM10 | 0.406324289 | 7.89119E-14 |
|  | FOXL2 | 0.547835786 | 8.07898E-14 |
|  | TCIRG1 | 0.619559438 | 8.22357E-14 |
|  | TLE2 | 0.685618222 | 8.24342E-14 |
|  | EMILIN1 | 0.685230834 | 8.32426E-14 |
|  | NFKBIL2 | 0.568421407 | 8.40609E-14 |
|  | HLX | 0.593491 | 8.46257E-14 |
|  | CHRNA10 | 0.434299659 | 8.51613E-14 |
|  | GRRP1 | 0.540456207 | 1.06869E-13 |
|  | VEGFA | 1.030078549 | 1.06904E-13 |
|  | IER5 | 0.437382891 | 1.1528E-13 |
|  | SERTAD1 | 0.598420417 | 1.19098E-13 |
|  | COL6A1 | 0.588430745 | 1.21776E-13 |
|  | BCAS4 | 0.505605452 | 1.46323E-13 |
|  | RADIL | 0.441842999 | 1.57977E-13 |
|  | NPIPL3 | 0.632366843 | 1.64252E-13 |
|  | LOC728264 | 0.693821885 | 1.79807E-13 |
|  | RASIP1 | 0.422368837 | 1.85827E-13 |
|  | NOL3 | 0.466438452 | 2.33242E-13 |
|  | RAB34 | 0.963811245 | 2.3531E-13 |
|  | ZNF530 | 0.460284286 | 2.4288E-13 |
|  | PDGFA | 0.74777027 | 2.47527E-13 |
|  | COL6A2 | 1.178966398 | 2.75128E-13 |
|  | FANCA | 0.718214967 | 2.7753E-13 |
|  | NUDT1 | 0.413806717 | 3.08761E-13 |
|  | NTAN1 | 0.403583406 | 3.3413E-13 |
|  | ATF5 | 0.49849257 | 3.66674E-13 |
|  | FCHSD1 | 0.564242919 | 3.77359E-13 |
|  | CDT1 | 0.726561949 | 3.87404E-13 |
|  | CCNE1 | 0.465221416 | 4.34118E-13 |
|  | PPIAL4G | 0.500808944 | 4.36423E-13 |
|  | LRDD | 0.505698026 | 4.57041E-13 |
|  | CSDA | 0.575470437 | 4.79274E-13 |
|  | GPR35 | 0.411855253 | 5.28675E-13 |
|  | PPP1R3G | 0.52207822 | 8.07457E-13 |
|  | LOC541471 | 1.158000344 | 8.10562E-13 |
|  | CHTF18 | 0.547212533 | 8.24691E-13 |
|  | EPS8L2 | 0.68495963 | 9.45712E-13 |
|  | TIMP1 | 1.43795043 | 9.89593E-13 |
|  | CCDC102A | 0.474671088 | 1.04919E-12 |
|  | IGFBP4 | 0.701925939 | 1.10601E-12 |
|  | EPHB4 | 0.501817497 | 1.21539E-12 |
|  | NCRNA00107 | 0.601649629 | 1.33509E-12 |
|  | MRPL41 | 0.409396554 | 1.43887E-12 |
|  | FHOD1 | 0.44431543 | 1.45863E-12 |
|  | C14orf80 | 0.472153424 | 1.58369E-12 |
|  | CRABP2 | 0.714692657 | 1.62849E-12 |
|  | B9D2 | 0.45293123 | 1.65423E-12 |
|  | SOCS1 | 0.822303073 | 1.75448E-12 |
|  | FKBP11 | 0.447790969 | 1.82048E-12 |
|  | CDCA3 | 0.593065028 | 2.14816E-12 |
|  | CD276 | 0.51446313 | 2.21471E-12 |
|  | ADM | 1.225858271 | 2.26502E-12 |
|  | CD97 | 0.704528797 | 2.52788E-12 |
|  | SHC1 | 0.482721093 | 2.6349E-12 |
|  | ENG | 0.505378681 | 2.64483E-12 |
|  | MYL9 | 0.660057888 | 2.71001E-12 |
|  | POC1A | 0.661470044 | 2.85494E-12 |
|  | CDCA4 | 0.536753288 | 3.05516E-12 |
|  | C1orf70 | 0.491530606 | 3.24815E-12 |
|  | CD63 | 0.468231453 | 3.25156E-12 |
|  | SMTN | 0.408707654 | 3.48024E-12 |
|  | PLK1 | 0.775286515 | 3.58536E-12 |
|  | GDF1 | 0.512389958 | 3.5878E-12 |
|  | SLC38A6 | 0.405299231 | 3.63529E-12 |
|  | TBX2 | 0.572301507 | 3.8389E-12 |
|  | WDR90 | 0.508654799 | 3.98087E-12 |
|  | COL5A1 | 1.281421432 | 4.15915E-12 |
|  | TAGLN | 0.733024059 | 4.19626E-12 |
|  | MYL6 | 0.405789717 | 4.78928E-12 |
|  | DLL4 | 0.567950576 | 4.9368E-12 |
|  | FAM43B | 0.52912617 | 4.99112E-12 |
|  | PARVB | 0.487875238 | 5.52E-12 |
|  | TAGLN2 | 0.850123965 | 5.58527E-12 |
|  | LEFTY1 | 0.565333711 | 6.96124E-12 |
|  | FHL3 | 0.469553908 | 7.60464E-12 |
|  | DPP7 | 0.41549337 | 7.73321E-12 |
|  | MSTO2P | 0.420960649 | 7.75485E-12 |
|  | C1QTNF6 | 0.536775484 | 8.69876E-12 |
|  | CLDN5 | 0.494149958 | 1.04767E-11 |
|  | IER2 | 0.480668494 | 1.06671E-11 |
|  | S1PR2 | 0.459445869 | 1.09437E-11 |
|  | BGLAP | 0.410624491 | 1.12922E-11 |
|  | A1BG | 0.609587074 | 1.16399E-11 |
|  | LOC113230 | 0.433122649 | 1.21761E-11 |
|  | STK40 | 0.411129967 | 1.30119E-11 |
|  | GPX8 | 1.221255794 | 1.31386E-11 |
|  | RPPH1 | 0.94718512 | 1.41994E-11 |
|  | ECSCR | 0.816507188 | 1.47888E-11 |
|  | FAM109B | 0.575051875 | 1.48466E-11 |
|  | RBP1 | 1.195043616 | 1.50382E-11 |
|  | ZNF692 | 0.459463984 | 1.52621E-11 |
|  | MAD2L2 | 0.412547589 | 1.60377E-11 |
|  | WDR62 | 0.845054185 | 1.68925E-11 |
|  | TACC3 | 0.742842569 | 1.71234E-11 |
|  | PTRF | 0.665931706 | 1.71282E-11 |
|  | LOC100129534 | 0.419482762 | 1.74614E-11 |
|  | PRICKLE3 | 0.676707024 | 1.79723E-11 |
|  | LOC100130776 | 1.027491444 | 1.81746E-11 |
|  | C10orf114 | 0.587955319 | 1.89991E-11 |
|  | TCEA3 | 0.921715903 | 1.98882E-11 |
|  | IGFBP6 | 0.817751364 | 2.05533E-11 |
|  | NSUN5P2 | 0.505165043 | 2.08357E-11 |
|  | C16orf75 | 0.693426674 | 2.32753E-11 |
|  | GSDMD | 0.610432839 | 2.5065E-11 |
|  | TRAIP | 0.488775186 | 2.9195E-11 |
|  | TMEM121 | 0.550848466 | 3.15463E-11 |
|  | ZDHHC12 | 0.532522218 | 3.24162E-11 |
|  | CFD | 0.773794208 | 3.3238E-11 |
|  | B3GNT7 | 0.819552987 | 3.45046E-11 |
|  | PCSK1N | 0.680533249 | 3.58807E-11 |
|  | LAT | 0.548974067 | 4.07634E-11 |
|  | ANKLE1 | 0.46516054 | 4.16949E-11 |
|  | LAMA5 | 0.422047046 | 4.38917E-11 |
|  | MMP11 | 0.907006019 | 4.40489E-11 |
|  | BATF3 | 0.721485023 | 4.50294E-11 |
|  | EMP3 | 1.373687609 | 4.54321E-11 |
|  | HEBP2 | 0.44371077 | 4.80502E-11 |
|  | MC1R | 0.434492579 | 5.21145E-11 |
|  | COL4A2 | 0.951075045 | 5.47731E-11 |
|  | OLFML2A | 0.589820577 | 5.52674E-11 |
|  | TTYH3 | 0.468051468 | 5.53324E-11 |
|  | LOX | 1.06367983 | 5.75473E-11 |
|  | LOC151534 | 0.55081111 | 6.08931E-11 |
|  | CITED1 | 0.789464551 | 6.18557E-11 |
|  | GHRLOS | 0.402853034 | 6.27995E-11 |
|  | LZTS1 | 0.758609376 | 6.36004E-11 |
|  | LGALS3 | 0.898668018 | 6.60106E-11 |
|  | C1orf113 | 0.540860245 | 6.60106E-11 |
|  | EFEMP2 | 0.825382544 | 6.98169E-11 |
|  | C1orf63 | 0.455260121 | 7.1054E-11 |
|  | RELB | 0.4568542 | 7.23453E-11 |
|  | PEX11G | 0.4035075 | 8.26024E-11 |
|  | ATP6V1C2 | 0.42697523 | 9.25957E-11 |
|  | FES | 0.448823104 | 9.29933E-11 |
|  | ZNF90 | 0.683390334 | 9.57693E-11 |
|  | EHD2 | 0.53881287 | 1.03238E-10 |
|  | FKBP9 | 0.664036643 | 1.15722E-10 |
|  | C16orf79 | 0.518480884 | 1.16138E-10 |
|  | VWA1 | 0.619418553 | 1.19322E-10 |
|  | C3orf47 | 0.43408781 | 1.2214E-10 |
|  | FBXO22OS | 0.413234757 | 1.24024E-10 |
|  | SEC61G | 0.907254551 | 1.32752E-10 |
|  | FLT3LG | 0.419238635 | 1.41423E-10 |
|  | NT5DC2 | 0.415776015 | 1.5141E-10 |
|  | ITPKC | 0.425210503 | 1.6302E-10 |
|  | RNF208 | 0.426569726 | 1.66575E-10 |
|  | PLXDC1 | 0.468143099 | 1.74595E-10 |
|  | MMP25 | 0.610781234 | 1.92322E-10 |
|  | COL16A1 | 0.577159405 | 1.96563E-10 |
|  | PTTG1 | 0.88545132 | 2.1104E-10 |
|  | DOT1L | 0.432687248 | 2.25796E-10 |
|  | ADAMTS7 | 0.741124982 | 2.54589E-10 |
|  | CISH | 0.71806918 | 2.59717E-10 |
|  | PDLIM1 | 0.883901698 | 2.95945E-10 |
|  | TMEM149 | 0.445248623 | 3.12223E-10 |
|  | ANPEP | 0.662947248 | 3.458E-10 |
|  | LOC283663 | 0.440023361 | 3.64923E-10 |
|  | DSN1 | 0.400543811 | 3.85805E-10 |
|  | LDHA | 0.629233439 | 4.21307E-10 |
|  | CUL7 | 0.414301701 | 4.24981E-10 |
|  | TMEM159 | 0.565279047 | 4.32266E-10 |
|  | LTB4R | 0.421964705 | 4.69302E-10 |
|  | KCTD14 | 0.610891472 | 4.79743E-10 |
|  | IL17RC | 0.50295541 | 4.96463E-10 |
|  | TNFRSF12A | 1.065980803 | 5.18892E-10 |
|  | C5orf39 | 0.540309724 | 5.20603E-10 |
|  | LPAR2 | 0.473516891 | 5.26311E-10 |
|  | PTPRCAP | 0.473836073 | 5.45381E-10 |
|  | CEACAM19 | 0.526395466 | 5.4701E-10 |
|  | PDIA4 | 0.437014941 | 5.48986E-10 |
|  | EDARADD | 0.701418405 | 5.57052E-10 |
|  | GPR4 | 0.475673126 | 5.95059E-10 |
|  | LRRC32 | 0.61486428 | 6.21329E-10 |
|  | GNG5 | 0.52594471 | 6.21329E-10 |
|  | LGALS1 | 0.669430854 | 6.95183E-10 |
|  | COL5A3 | 0.542975453 | 6.97446E-10 |
|  | DDB2 | 0.49570694 | 7.31533E-10 |
|  | LAMC3 | 0.578337378 | 7.52739E-10 |
|  | SNHG11 | 0.547063602 | 7.67894E-10 |
|  | SMAGP | 0.573808821 | 8.23033E-10 |
|  | TRIP10 | 0.442315886 | 8.92321E-10 |
|  | OAF | 0.453762695 | 8.9458E-10 |
|  | AURKA | 0.638150345 | 9.10507E-10 |
|  | SLC25A45 | 0.490732619 | 9.13925E-10 |
|  | HSPB6 | 0.823681691 | 9.25071E-10 |
|  | FSCN1 | 0.438901395 | 9.38673E-10 |
|  | FGFRL1 | 0.613797008 | 9.53808E-10 |
|  | CLEC18A | 0.706709205 | 9.65559E-10 |
|  | ACADS | 0.460639079 | 9.79437E-10 |
|  | NANOS3 | 0.410308337 | 9.82586E-10 |
|  | CHCHD10 | 0.485738808 | 1.13959E-09 |
|  | NCRNA00105 | 0.583347183 | 1.17287E-09 |
|  | RPS26 | 0.46364896 | 1.19909E-09 |
|  | PRDX4 | 0.421250414 | 1.23589E-09 |
|  | DOCK6 | 0.408916778 | 1.26532E-09 |
|  | PLP2 | 0.841095409 | 1.35785E-09 |
|  | C10orf47 | 0.447567747 | 1.39613E-09 |
|  | RARRES2 | 1.180105976 | 1.40222E-09 |
|  | TPM4 | 0.406823643 | 1.41435E-09 |
|  | ARID5A | 0.42828882 | 1.54731E-09 |
|  | CCNL2 | 0.440458753 | 1.55079E-09 |
|  | GJC1 | 0.604429508 | 1.61912E-09 |
|  | CCNB1 | 0.682361972 | 1.69134E-09 |
|  | IMPA2 | 0.444933448 | 1.70634E-09 |
|  | FBLIM1 | 0.715383633 | 1.71022E-09 |
|  | ISG20 | 0.669980302 | 1.88404E-09 |
|  | RAD54L | 0.733429397 | 1.91936E-09 |
|  | TK1 | 0.78980713 | 2.00842E-09 |
|  | TUBB6 | 0.623871736 | 2.06239E-09 |
|  | ATP5EP2 | 0.635727569 | 2.07168E-09 |
|  | TEAD3 | 0.707927457 | 2.10037E-09 |
|  | CEL | 0.459348627 | 2.11418E-09 |
|  | HLA-H | 0.475010909 | 2.14739E-09 |
|  | GADD45A | 0.608084451 | 2.30407E-09 |
|  | PTX3 | 1.061932363 | 2.37545E-09 |
|  | NXPH4 | 0.701384963 | 2.46612E-09 |
|  | TRIP6 | 0.54037335 | 2.47402E-09 |
|  | C9orf89 | 0.493057176 | 2.58471E-09 |
|  | CHAF1B | 0.667271263 | 2.64398E-09 |
|  | ADAM19 | 0.600298712 | 2.7784E-09 |
|  | EPHA2 | 0.598518379 | 2.91266E-09 |
|  | PPP1R15A | 0.408463352 | 2.96658E-09 |
|  | TRIB3 | 0.579104619 | 2.98893E-09 |
|  | MUC1 | 0.505354799 | 2.99832E-09 |
|  | RRAS | 0.404299331 | 3.37094E-09 |
|  | FZD8 | 0.515172064 | 3.40777E-09 |
|  | CDK3 | 0.429185596 | 3.54838E-09 |
|  | CENPW | 0.559548807 | 3.67574E-09 |
|  | DUSP23 | 0.456476172 | 3.77046E-09 |
|  | MYO1G | 0.827564615 | 4.12744E-09 |
|  | CDKN2C | 0.719331995 | 4.39829E-09 |
|  | THSD1 | 0.410954718 | 4.64923E-09 |
|  | MCAM | 0.507881231 | 4.68995E-09 |
|  | CDKN3 | 0.736795616 | 4.85895E-09 |
|  | PILRB | 0.471228277 | 5.08697E-09 |
|  | PSRC1 | 0.615645098 | 5.15407E-09 |
|  | C17orf53 | 0.468491329 | 5.22476E-09 |
|  | HSD3B7 | 0.447774762 | 5.26447E-09 |
|  | H2AFJ | 0.411265539 | 5.85495E-09 |
|  | FKBP10 | 0.524943094 | 6.05391E-09 |
|  | CLIC1 | 0.706638588 | 6.09461E-09 |
|  | LOC100132215 | 0.472261876 | 6.09599E-09 |
|  | HSPG2 | 0.686613516 | 6.20098E-09 |
|  | FBXO17 | 0.871190882 | 6.22951E-09 |
|  | ORC6L | 0.524888493 | 6.49516E-09 |
|  | TWIST1 | 0.736505588 | 6.62852E-09 |
|  | CCDC74A | 0.414319736 | 6.71338E-09 |
|  | RHOC | 0.423233242 | 6.79256E-09 |
|  | CKS2 | 0.594841285 | 6.84052E-09 |
|  | S100A6 | 0.608907615 | 6.91077E-09 |
|  | MXRA8 | 0.514244062 | 7.25503E-09 |
|  | ASF1B | 0.855729597 | 7.59471E-09 |
|  | GJA4 | 0.465713141 | 8.06875E-09 |
|  | ADAMTSL4 | 0.614584425 | 8.08298E-09 |
|  | AFAP1L1 | 0.56513634 | 8.22268E-09 |
|  | TEAD4 | 0.709139372 | 8.58685E-09 |
|  | SSC5D | 0.555978798 | 8.79897E-09 |
|  | SPRY1 | 0.687296841 | 8.93017E-09 |
|  | LRRC46 | 0.511573939 | 9.33371E-09 |
|  | RHOJ | 0.501759112 | 9.54832E-09 |
|  | MAP3K6 | 0.41316568 | 9.92079E-09 |
|  | RHBDF1 | 0.480770173 | 1.06014E-08 |
|  | VAV3 | 1.038951 | 1.22212E-08 |
|  | IRF7 | 0.500352371 | 1.2294E-08 |
|  | RINL | 0.490631704 | 1.27781E-08 |
|  | ANG | 0.640523726 | 1.28236E-08 |
|  | MDK | 0.725492609 | 1.32043E-08 |
|  | FZD2 | 0.501915954 | 1.33839E-08 |
|  | PLAT | 0.817030187 | 1.3423E-08 |
|  | LOC100270710 | 0.582065233 | 1.35362E-08 |
|  | GPX7 | 0.48213252 | 1.39834E-08 |
|  | C9orf100 | 0.451878194 | 1.40603E-08 |
|  | ADAM8 | 0.623781712 | 1.41388E-08 |
|  | PKIB | 0.875256768 | 1.44164E-08 |
|  | RPS27 | 0.932788243 | 1.74685E-08 |
|  | PMS2L2 | 0.458036143 | 1.79623E-08 |
|  | IFITM3 | 0.5767023 | 1.83066E-08 |
|  | S100A4 | 0.970234568 | 1.85372E-08 |
|  | CNIH3 | 0.502322241 | 1.91218E-08 |
|  | FLT4 | 0.446355962 | 1.91479E-08 |
|  | NCRNA00085 | 0.414335881 | 1.92237E-08 |
|  | LAMB1 | 0.708133336 | 2.01855E-08 |
|  | COL1A1 | 1.231120454 | 2.04138E-08 |
|  | ACTN1 | 0.615772577 | 2.04285E-08 |
|  | LY6E | 0.424789004 | 2.19114E-08 |
|  | PDIA5 | 0.555281863 | 2.20019E-08 |
|  | GPR3 | 0.726970046 | 2.29549E-08 |
|  | CENPH | 0.448289746 | 2.36295E-08 |
|  | ARL4C | 0.607056555 | 2.42422E-08 |
|  | KLHDC8A | 0.700504801 | 2.46198E-08 |
|  | TSPO | 0.486163725 | 2.61075E-08 |
|  | OCIAD2 | 0.929073592 | 2.76085E-08 |
|  | ACE | 0.654642005 | 2.78225E-08 |
|  | COL9A3 | 0.854484115 | 3.3186E-08 |
|  | LOC400931 | 0.495040526 | 3.53127E-08 |
|  | TUBA1C | 0.787819469 | 3.57326E-08 |
|  | TRIP13 | 0.56256439 | 3.5979E-08 |
|  | PRR22 | 0.420324577 | 4.02241E-08 |
|  | ANXA2 | 0.733477809 | 4.27405E-08 |
|  | COL1A2 | 0.828964919 | 4.32298E-08 |
|  | UBE2T | 0.602917702 | 4.60161E-08 |
|  | CCDC78 | 0.58286897 | 4.74174E-08 |
|  | RAB32 | 0.580535146 | 4.80624E-08 |
|  | CENPM | 0.625064521 | 5.37326E-08 |
|  | GPR89C | 0.49454151 | 5.66056E-08 |
|  | LXN | 0.547362518 | 5.7007E-08 |
|  | C21orf63 | 0.557638205 | 5.8014E-08 |
|  | TYMP | 0.77687149 | 5.83846E-08 |
|  | CCDC8 | 0.748720548 | 5.88282E-08 |
|  | GGH | 0.434536168 | 5.97063E-08 |
|  | HLA-A | 0.414326437 | 6.2475E-08 |
|  | COL3A1 | 1.202001049 | 6.4162E-08 |
|  | UNC93B1 | 0.465306899 | 6.58295E-08 |
|  | CEBPB | 0.426587422 | 7.16351E-08 |
|  | VMO1 | 0.420453676 | 7.38982E-08 |
|  | MRC2 | 0.578906434 | 7.41344E-08 |
|  | LPIN3 | 0.553705686 | 7.54791E-08 |
|  | NODAL | 0.631735729 | 7.56175E-08 |
|  | SLC16A3 | 0.591922037 | 7.95838E-08 |
|  | ADAMTS14 | 0.721384393 | 8.32751E-08 |
|  | TCF19 | 0.445357119 | 8.64993E-08 |
|  | COL5A2 | 0.77252193 | 8.7864E-08 |
|  | RNF135 | 0.457438125 | 8.79526E-08 |
|  | RDH16 | 0.432987824 | 9.04603E-08 |
|  | C5orf62 | 0.574042909 | 9.30863E-08 |
|  | PPIEL | 0.60147435 | 9.31712E-08 |
|  | ARHGEF19 | 0.438844165 | 9.67712E-08 |
|  | DBNDD1 | 0.518123709 | 9.79372E-08 |
|  | SHCBP1 | 0.622131677 | 1.05293E-07 |
|  | MSH5 | 0.443964692 | 1.12588E-07 |
|  | CTHRC1 | 0.839692716 | 1.13762E-07 |
|  | ZDHHC1 | 0.517513293 | 1.16654E-07 |
|  | PPIC | 0.529818342 | 1.185E-07 |
|  | GPR39 | 0.594675247 | 1.18848E-07 |
|  | GINS1 | 0.481140543 | 1.19947E-07 |
|  | COL4A1 | 0.901597304 | 1.21446E-07 |
|  | PICK1 | 0.406884932 | 1.26542E-07 |
|  | GSDMB | 0.456513066 | 1.32476E-07 |
|  | NUAK2 | 0.520459892 | 1.36726E-07 |
|  | NPR1 | 0.595593726 | 1.38306E-07 |
|  | RBPMS2 | 0.401390108 | 1.42099E-07 |
|  | GINS2 | 0.556830098 | 1.44835E-07 |
|  | SPRY4 | 0.811454705 | 1.47379E-07 |
|  | KDELR3 | 0.698101682 | 1.51042E-07 |
|  | PCSK4 | 0.413246628 | 1.52836E-07 |
|  | PLAU | 0.825670768 | 1.53789E-07 |
|  | CAPS | 0.543240442 | 1.57162E-07 |
|  | IGFBP7 | 0.467522741 | 1.63674E-07 |
|  | HSPA6 | 0.783627638 | 1.66404E-07 |
|  | RAD54B | 0.478568223 | 1.7212E-07 |
|  | ITGA7 | 0.486296497 | 1.73847E-07 |
|  | ANXA1 | 0.937539307 | 1.8644E-07 |
|  | PLAUR | 0.665574322 | 1.86824E-07 |
|  | FLNA | 0.444181313 | 1.95362E-07 |
|  | CDC6 | 0.615798189 | 2.18327E-07 |
|  | ZBTB42 | 0.479676483 | 2.26266E-07 |
|  | CCDC109B | 0.736963784 | 2.4081E-07 |
|  | VIM | 0.687608049 | 2.44618E-07 |
|  | TMSL3 | 0.406263649 | 2.50909E-07 |
|  | RBPMS | 0.459696786 | 2.59195E-07 |
|  | SH2B2 | 0.414067934 | 2.62687E-07 |
|  | HK3 | 0.685652599 | 2.62937E-07 |
|  | ANGPT2 | 0.752169075 | 2.69306E-07 |
|  | SLC43A3 | 0.640684826 | 2.72338E-07 |
|  | EMILIN2 | 0.705074299 | 2.79269E-07 |
|  | CNN2 | 0.537665124 | 2.87564E-07 |
|  | C9orf172 | 0.404115842 | 3.33732E-07 |
|  | KIAA0495 | 0.541440264 | 3.75282E-07 |
|  | RDH5 | 0.49575046 | 3.81092E-07 |
|  | FOXF2 | 0.422187147 | 3.96118E-07 |
|  | SSH3 | 0.419470131 | 4.23454E-07 |
|  | CRYGS | 0.446692158 | 4.78309E-07 |
|  | VAMP5 | 0.455677251 | 4.79043E-07 |
|  | C7orf68 | 0.497289989 | 4.89416E-07 |
|  | IRF1 | 0.468665874 | 5.01129E-07 |
|  | ABCA7 | 0.410781841 | 5.01248E-07 |
|  | KIF2C | 0.777979749 | 5.10308E-07 |
|  | XKR8 | 0.534832779 | 5.2962E-07 |
|  | C12orf48 | 0.505957453 | 5.36059E-07 |
|  | ITGA3 | 0.497926654 | 5.3936E-07 |
|  | CDCA8 | 0.71510371 | 5.4898E-07 |
|  | TGIF1 | 0.488961412 | 5.68158E-07 |
|  | EME1 | 0.477626578 | 5.85488E-07 |
|  | A2LD1 | 0.454921293 | 5.8796E-07 |
|  | ACTA2 | 0.536854064 | 5.90399E-07 |
|  | CDCA5 | 0.537792711 | 6.06266E-07 |
|  | PCDH12 | 0.499676548 | 6.28294E-07 |
|  | PIK3R6 | 0.453325151 | 6.48924E-07 |
|  | ZMYND10 | 0.63558824 | 6.49708E-07 |
|  | MCM2 | 0.464564912 | 6.52106E-07 |
|  | TYMS | 0.636068544 | 7.26253E-07 |
|  | PLEKHA4 | 0.638899181 | 7.45557E-07 |
|  | ADAM12 | 0.856599218 | 7.87432E-07 |
|  | SLC17A9 | 0.432304989 | 8.40051E-07 |
|  | PYCARD | 0.447110869 | 8.46707E-07 |
|  | CD101 | 0.541675374 | 8.54503E-07 |
|  | CAV2 | 0.496005926 | 8.75325E-07 |
|  | SPAG5 | 0.463291797 | 8.776E-07 |
|  | NPNT | 0.805068747 | 8.93496E-07 |
|  | BACE2 | 0.454745816 | 9.04076E-07 |
|  | SP6 | 0.468024941 | 9.38517E-07 |
|  | YJEFN3 | 0.477909579 | 1.00759E-06 |
|  | HDHD3 | 0.508435666 | 1.03815E-06 |
|  | C10orf10 | 0.522507948 | 1.04764E-06 |
|  | SOCS2 | 0.638343375 | 1.04867E-06 |
|  | TNFAIP2 | 0.4729402 | 1.05164E-06 |
|  | CCNA2 | 0.624947651 | 1.11093E-06 |
|  | ITPRIPL1 | 0.586595346 | 1.2468E-06 |
|  | RAD51 | 0.601952291 | 1.32873E-06 |
|  | ACCS | 0.517451168 | 1.36345E-06 |
|  | ANXA2P2 | 0.773131993 | 1.39618E-06 |
|  | VWF | 0.406781118 | 1.44585E-06 |
|  | METTL1 | 0.466946695 | 1.46722E-06 |
|  | S100A13 | 0.499543635 | 1.55629E-06 |
|  | FOXM1 | 0.704776622 | 1.59764E-06 |
|  | ARSE | 0.455297897 | 1.69618E-06 |
|  | MMP14 | 0.569201562 | 1.71182E-06 |
|  | WDR66 | 0.473470168 | 1.93338E-06 |
|  | SERINC2 | 0.636145839 | 2.08081E-06 |
|  | MREG | 0.407115944 | 2.11956E-06 |
|  | SLC4A3 | 0.424211804 | 2.13303E-06 |
|  | DIRAS3 | 0.692203988 | 2.44557E-06 |
|  | LSP1 | 0.691579587 | 2.52209E-06 |
|  | MAP1LC3A | 0.431263453 | 2.7464E-06 |
|  | DUSP6 | 0.466255102 | 2.87128E-06 |
|  | SEL1L3 | 0.697598407 | 2.91301E-06 |
|  | TM4SF1 | 0.533854418 | 2.98044E-06 |
|  | LOC441869 | 0.46302474 | 3.08297E-06 |
|  | ORC1L | 0.646897382 | 3.11925E-06 |
|  | CHI3L1 | 1.43413542 | 3.14091E-06 |
|  | SOCS3 | 0.834697822 | 3.16063E-06 |
|  | WEE1 | 0.518178038 | 3.30872E-06 |
|  | STEAP3 | 0.712377972 | 3.40227E-06 |
|  | ITGA5 | 0.494131147 | 3.9072E-06 |
|  | SELM | 0.440488166 | 3.93165E-06 |
|  | TNFRSF1A | 0.417470495 | 3.96764E-06 |
|  | MLF1IP | 0.617320359 | 4.11165E-06 |
|  | AVIL | 0.517757099 | 4.17094E-06 |
|  | TEAD2 | 0.492731838 | 4.64615E-06 |
|  | CARD16 | 0.60206931 | 4.67437E-06 |
|  | HIST1H2BK | 0.407117164 | 4.81416E-06 |
|  | IFI6 | 0.539192289 | 4.95266E-06 |
|  | ST8SIA5 | 0.612361098 | 5.18757E-06 |
|  | MST1P2 | 0.492550375 | 5.22177E-06 |
|  | JUNB | 0.473707451 | 5.35597E-06 |
|  | SCNN1D | 0.424832737 | 5.54366E-06 |
|  | LOXL2 | 0.575988501 | 5.7609E-06 |
|  | PLIN2 | 0.449022944 | 5.79285E-06 |
|  | FCGBP | 0.803210678 | 6.76385E-06 |
|  | TPX2 | 0.612394676 | 6.93954E-06 |
|  | PLAC9 | 0.484476122 | 7.22485E-06 |
|  | IGF2BP3 | 0.867575251 | 7.28099E-06 |
|  | FSTL1 | 0.485887787 | 7.28814E-06 |
|  | SECTM1 | 0.626028897 | 7.38341E-06 |
|  | DDX12 | 0.423996647 | 7.71485E-06 |
|  | ENPEP | 0.61012043 | 7.83027E-06 |
|  | MARVELD1 | 0.447756557 | 8.28739E-06 |
|  | PYGL | 0.455918418 | 8.63377E-06 |
|  | SPHK1 | 0.449663376 | 8.63793E-06 |
|  | PTGES | 0.475887706 | 8.65025E-06 |
|  | BST2 | 0.470643691 | 8.99524E-06 |
|  | IFI30 | 0.647448619 | 9.46891E-06 |
|  | ISG15 | 0.558053044 | 9.6134E-06 |
|  | CD72 | 0.417703619 | 1.0595E-05 |
|  | ANGPTL4 | 0.531790078 | 1.17094E-05 |
|  | MBOAT1 | 0.435426827 | 1.22582E-05 |
|  | METTL7B | 0.888820704 | 1.28454E-05 |
|  | ATAD3C | 0.457714749 | 1.28629E-05 |
|  | PRC1 | 0.431308842 | 1.31804E-05 |
|  | S100A11 | 0.541356646 | 1.44128E-05 |
|  | CCDC3 | 0.445933686 | 1.45624E-05 |
|  | PTGFRN | 0.409627115 | 1.45863E-05 |
|  | COL7A1 | 0.554288537 | 1.47208E-05 |
|  | CAV1 | 0.540396783 | 1.47592E-05 |
|  | FAM84A | 0.420167944 | 1.48364E-05 |
|  | NOS2 | 0.615982908 | 1.61011E-05 |
|  | PDPN | 0.929849601 | 1.61405E-05 |
|  | ETV4 | 0.937008987 | 1.85241E-05 |
|  | LTBP4 | 0.427374782 | 1.86128E-05 |
|  | FABP7 | 0.609933931 | 2.05445E-05 |
|  | SPOCD1 | 0.952123701 | 2.24489E-05 |
|  | TMEM45A | 0.414267281 | 2.26367E-05 |
|  | SOD2 | 0.481513309 | 2.39664E-05 |
|  | APOL1 | 0.440351203 | 2.43175E-05 |
|  | SKA3 | 0.584037345 | 2.81048E-05 |
|  | AEBP1 | 0.728117676 | 2.88608E-05 |
|  | HOPX | 0.557588285 | 2.88923E-05 |
|  | IGFBP3 | 0.705709639 | 2.90589E-05 |
|  | ICAM1 | 0.503058163 | 2.92145E-05 |
|  | RDH10 | 0.459954618 | 2.95004E-05 |
|  | C13orf18 | 0.468715273 | 3.11433E-05 |
|  | FMOD | 0.85502896 | 3.18977E-05 |
|  | BCAT1 | 0.583659355 | 3.28598E-05 |
|  | C21orf7 | 0.60534359 | 3.29124E-05 |
|  | GNB3 | 0.461152897 | 3.38106E-05 |
|  | FAM115C | 0.527098329 | 3.96899E-05 |
|  | FOXJ1 | 0.602984143 | 4.22526E-05 |
|  | LUM | 0.671167775 | 4.33377E-05 |
|  | NMNAT3 | 0.548055742 | 4.46063E-05 |
|  | C1RL | 0.472094551 | 4.60206E-05 |
|  | DUSP5 | 0.553073851 | 4.61367E-05 |
|  | SERPING1 | 0.537869088 | 5.0749E-05 |
|  | CLEC2B | 0.415159635 | 5.15715E-05 |
|  | SPEF1 | 0.501083603 | 5.45066E-05 |
|  | RHBDL1 | 0.413220988 | 6.23875E-05 |
|  | KCNF1 | 0.499113763 | 6.78528E-05 |
|  | IL32 | 0.437854327 | 7.01952E-05 |
|  | EZH2 | 0.408474583 | 7.20398E-05 |
|  | FOXD1 | 0.500874314 | 7.80312E-05 |
|  | GTSE1 | 0.569742621 | 8.42714E-05 |
|  | FHL2 | 0.445013763 | 8.81261E-05 |
|  | JAK3 | 0.446346169 | 8.93727E-05 |
|  | CBR1 | 0.466070242 | 9.71575E-05 |
|  | CD93 | 0.483588585 | 0.000104476 |
|  | NID2 | 0.423301749 | 0.000109129 |
|  | KIAA0040 | 0.489774797 | 0.000109201 |
|  | MGP | 0.649194733 | 0.000122908 |
|  | MAPK13 | 0.411556374 | 0.000122908 |
|  | NCF1 | 0.439757817 | 0.000128535 |
|  | C1R | 0.511800864 | 0.000137299 |
|  | THBD | 0.471813565 | 0.000137519 |
|  | PLVAP | 0.422353586 | 0.00013912 |
|  | MT3 | 0.445867251 | 0.000143233 |
|  | MSMP | 0.400204448 | 0.000145636 |
|  | SLC47A1 | 0.479485831 | 0.00017444 |
|  | CASP4 | 0.433643212 | 0.000185578 |
|  | SHROOM3 | 0.543016847 | 0.000186326 |
|  | COX7A1 | 0.436799822 | 0.000200024 |
|  | CD58 | 0.415879753 | 0.000200246 |
|  | TNFAIP8 | 0.406384856 | 0.00020987 |
|  | CDK1 | 0.538633186 | 0.000212796 |
|  | FBLN1 | 0.423148922 | 0.000215752 |
|  | SNAI2 | 0.451290513 | 0.000220801 |
|  | NCAPH | 0.500577452 | 0.000225291 |
|  | CAPG | 0.4364319 | 0.000236579 |
|  | COL12A1 | 0.458218645 | 0.000246143 |
|  | OSBPL10 | 0.409459555 | 0.000248789 |
|  | COL6A3 | 0.699015204 | 0.000250703 |
|  | KIF4A | 0.530328633 | 0.000298008 |
|  | CMYA5 | 0.525184807 | 0.000303674 |
|  | CTSK | 0.414538031 | 0.000309985 |
|  | NR2E1 | 0.430085766 | 0.000340389 |
|  | DUSP4 | 0.546805691 | 0.000343769 |
|  | SFRP4 | 0.541461288 | 0.000344686 |
|  | CENPE | 0.491032485 | 0.000353688 |
|  | ST14 | 0.493399453 | 0.00037348 |
|  | KCNE4 | 0.463013443 | 0.000396707 |
|  | KIFC1 | 0.515733472 | 0.000450044 |
|  | IGFBP5 | 0.401819674 | 0.000498162 |
|  | F3 | 0.422923193 | 0.000516217 |
|  | NUF2 | 0.457736379 | 0.000549696 |
|  | BUB1 | 0.546635721 | 0.000554048 |
|  | ARC | 0.463342593 | 0.000574158 |
|  | NGFR | 0.466239204 | 0.000623597 |
|  | TGFBI | 0.550281682 | 0.000635992 |
|  | RGS16 | 0.450677599 | 0.000741358 |
|  | CELSR1 | 0.53104498 | 0.000921932 |
|  | FAM26F | 0.428824465 | 0.001098011 |
|  | SERPINE1 | 0.662004747 | 0.001106398 |
|  | AIM1 | 0.429293398 | 0.001156583 |
|  | CFB | 0.402848475 | 0.00129119 |
|  | IGF2 | 0.418914162 | 0.00143501 |
|  | ACP5 | 0.445235362 | 0.001843801 |
|  | NUSAP1 | 0.461243666 | 0.001886227 |
|  | CYR61 | 0.427921863 | 0.002982429 |
|  | DTL | 0.422045604 | 0.003435037 |
|  | CD36 | 0.449195822 | 0.00346316 |
|  | CA12 | 0.428603196 | 0.005091024 |
|  | GPNMB | 0.468594773 | 0.005145849 |
|  | SLC11A1 | 0.405621346 | 0.005863906 |
|  | HLA-DQB1 | 0.455975253 | 0.006938853 |
|  | CENPV | 0.40124645 | 0.013497556 |
|  | CD163 | 0.506074787 | 0.018531223 |
| **Downregulated** | TBCEL | -0.541600234 | 2.48442E-37 |
|  | GPR75 | -1.099187519 | 1.2602E-36 |
|  | WDFY3 | -0.683484852 | 3.80736E-36 |
|  | C5orf41 | -0.823010239 | 7.5707E-36 |
|  | BBX | -0.513179415 | 1.51537E-35 |
|  | SAMD8 | -1.109013928 | 4.34123E-35 |
|  | RAB30 | -0.853009358 | 5.45127E-34 |
|  | GAN | -0.840948271 | 6.20201E-34 |
|  | FAM199X | -0.615750016 | 2.81846E-33 |
|  | DPP8 | -0.875355943 | 6.24775E-33 |
|  | NBEAL1 | -1.256095582 | 7.36797E-33 |
|  | PURA | -0.502210546 | 9.79092E-33 |
|  | CLCN3 | -0.590005665 | 2.47626E-32 |
|  | OGFRL1 | -0.924528299 | 3.28887E-32 |
|  | RC3H2 | -0.763889735 | 5.58377E-32 |
|  | KLHL5 | -0.552103078 | 9.50076E-32 |
|  | UBXN7 | -0.915988352 | 1.04008E-31 |
|  | ADD3 | -0.768269958 | 1.43129E-31 |
|  | ZC3H12C | -0.474450165 | 1.62083E-31 |
|  | CEP350 | -0.56493186 | 2.63107E-31 |
|  | PIAS1 | -0.524272426 | 3.37675E-31 |
|  | NHLRC2 | -0.921949514 | 5.44273E-31 |
|  | CEP97 | -0.778234584 | 5.73298E-31 |
|  | DMXL1 | -0.513620319 | 6.75607E-31 |
|  | ASXL2 | -0.910559554 | 7.88206E-31 |
|  | C9orf5 | -0.433850245 | 7.9278E-31 |
|  | DENND4A | -0.52799729 | 1.12932E-30 |
|  | ANKRD44 | -0.835501962 | 2.30868E-30 |
|  | TAOK1 | -1.286433058 | 2.47E-30 |
|  | FAM149B1 | -0.478610593 | 4.99237E-30 |
|  | C4orf12 | -0.947536298 | 8.27139E-30 |
|  | RABGAP1L | -0.704294975 | 1.12258E-29 |
|  | RCBTB1 | -0.49965894 | 1.37884E-29 |
|  | STRN | -0.840626546 | 2.56049E-29 |
|  | CCDC75 | -0.747677799 | 2.83095E-29 |
|  | FSD1L | -0.884728025 | 4.71199E-29 |
|  | DNAJB14 | -0.571675258 | 7.21839E-29 |
|  | HMBOX1 | -0.818014452 | 8.02812E-29 |
|  | KCNN3 | -1.2277334 | 1.10954E-28 |
|  | FAM169A | -0.650258503 | 1.31912E-28 |
|  | TMCC3 | -0.614176701 | 1.42422E-28 |
|  | ZNF81 | -0.794940947 | 1.77836E-28 |
|  | RAPGEF6 | -0.79391427 | 2.1502E-28 |
|  | PPM1L | -1.128099104 | 3.67848E-28 |
|  | SESTD1 | -0.425443859 | 3.80338E-28 |
|  | USP32 | -0.419512088 | 4.04615E-28 |
|  | C1orf58 | -0.585613692 | 5.98876E-28 |
|  | C10orf118 | -0.727088717 | 8.72129E-28 |
|  | MAP3K2 | -0.591902604 | 1.18873E-27 |
|  | NCOA2 | -1.050176089 | 1.1991E-27 |
|  | SECISBP2L | -0.578330483 | 1.77411E-27 |
|  | ADAM22 | -1.060588578 | 1.81207E-27 |
|  | IL6ST | -1.134299184 | 5.05204E-27 |
|  | MYO9A | -0.904498386 | 5.61947E-27 |
|  | YIPF6 | -0.580031741 | 7.2402E-27 |
|  | ANKIB1 | -0.448755697 | 8.82911E-27 |
|  | TNKS2 | -0.468440606 | 9.8175E-27 |
|  | ZNF641 | -0.541840338 | 1.11544E-26 |
|  | ARHGAP12 | -0.546480578 | 1.291E-26 |
|  | BIRC6 | -0.526306613 | 1.38159E-26 |
|  | PIKFYVE | -0.499718685 | 1.97391E-26 |
|  | SFMBT2 | -0.896696463 | 2.08095E-26 |
|  | LMBRD2 | -0.91327558 | 2.21968E-26 |
|  | SULT1C4 | -0.8664962 | 2.75529E-26 |
|  | TCP11L2 | -0.693810613 | 4.14492E-26 |
|  | EXOC6B | -0.804459455 | 5.04783E-26 |
|  | UHRF1BP1L | -0.439195645 | 5.46224E-26 |
|  | OSBPL11 | -0.804089577 | 5.66996E-26 |
|  | PDP2 | -0.608434904 | 7.74695E-26 |
|  | KPNA5 | -0.833578953 | 9.755E-26 |
|  | ALS2CR8 | -0.514617772 | 1.30291E-25 |
|  | HIPK3 | -0.818647905 | 1.31773E-25 |
|  | ZNF678 | -0.8300009 | 1.86383E-25 |
|  | ASH1L | -0.537826399 | 2.19375E-25 |
|  | FNIP2 | -0.705177582 | 2.20583E-25 |
|  | RPS6KA5 | -0.86518385 | 2.29173E-25 |
|  | PDZD8 | -0.799615908 | 2.46137E-25 |
|  | TTBK2 | -0.956039466 | 2.47563E-25 |
|  | NCOA1 | -0.425102628 | 2.80288E-25 |
|  | FAM171B | -0.639046564 | 3.10633E-25 |
|  | BMP2K | -0.625418624 | 3.16723E-25 |
|  | ATE1 | -0.783115187 | 3.4023E-25 |
|  | MTM1 | -0.44871313 | 3.40945E-25 |
|  | RBM41 | -0.419413036 | 3.88763E-25 |
|  | RUNDC2A | -0.84784611 | 3.99455E-25 |
|  | VPS13B | -0.491041205 | 4.48498E-25 |
|  | MED13 | -0.410222232 | 5.06425E-25 |
|  | FMNL2 | -0.479864104 | 6.26681E-25 |
|  | FAM63B | -0.899188633 | 8.52555E-25 |
|  | C2orf49 | -0.440686109 | 9.52558E-25 |
|  | TNIK | -0.701707042 | 1.35171E-24 |
|  | ZKSCAN1 | -0.754494495 | 1.73268E-24 |
|  | KIF2A | -0.522337577 | 1.90904E-24 |
|  | C9orf102 | -0.918871827 | 1.98766E-24 |
|  | CNTN1 | -1.288086009 | 2.3234E-24 |
|  | KLF7 | -0.631032162 | 2.47415E-24 |
|  | SLC30A1 | -0.474041905 | 2.72806E-24 |
|  | IKZF5 | -0.478258278 | 2.77831E-24 |
|  | C18orf1 | -0.560833892 | 3.2506E-24 |
|  | LNPEP | -0.832551422 | 3.32016E-24 |
|  | NR2C2 | -0.477876257 | 3.3544E-24 |
|  | MAPRE2 | -0.406606272 | 3.39708E-24 |
|  | KLHL28 | -0.539992544 | 4.37685E-24 |
|  | PANK3 | -0.575564531 | 4.50915E-24 |
|  | SC5DL | -0.454462894 | 5.066E-24 |
|  | MPDZ | -0.589688097 | 5.58905E-24 |
|  | ZDHHC21 | -0.60949404 | 5.68356E-24 |
|  | LATS1 | -0.752943141 | 5.81954E-24 |
|  | FAM45B | -0.477859929 | 6.46004E-24 |
|  | MGAT5 | -1.007637297 | 6.47583E-24 |
|  | RFX3 | -0.748978844 | 7.16417E-24 |
|  | C20orf177 | -0.682773109 | 7.85637E-24 |
|  | TLR4 | -0.870501003 | 8.53931E-24 |
|  | FAM168A | -0.890738799 | 9.82638E-24 |
|  | DENND4C | -0.408670331 | 1.09728E-23 |
|  | USP12 | -0.73307665 | 1.15267E-23 |
|  | KIAA1109 | -0.639844646 | 1.18701E-23 |
|  | AKAP11 | -0.573414207 | 1.35951E-23 |
|  | MAN1A2 | -0.626243532 | 1.43249E-23 |
|  | PPARA | -0.60362215 | 1.50208E-23 |
|  | TBC1D12 | -0.489309956 | 1.50307E-23 |
|  | ARL5B | -0.767395815 | 1.57651E-23 |
|  | UBR2 | -0.410196323 | 1.88E-23 |
|  | CCDC88A | -0.454056639 | 1.88312E-23 |
|  | KIAA2026 | -0.565493148 | 1.91411E-23 |
|  | C15orf38 | -0.425881936 | 2.04007E-23 |
|  | WDR31 | -0.737856504 | 2.11959E-23 |
|  | USP34 | -0.447613982 | 2.43531E-23 |
|  | ZNF619 | -0.509282819 | 3.41767E-23 |
|  | SLK | -0.487849146 | 4.94666E-23 |
|  | SKIL | -0.545420192 | 4.94666E-23 |
|  | NF1 | -0.566900574 | 5.82743E-23 |
|  | RIF1 | -0.593437843 | 6.13226E-23 |
|  | NPAT | -0.416263075 | 6.16062E-23 |
|  | ERCC6 | -0.592979053 | 6.92126E-23 |
|  | ATF2 | -0.516367144 | 7.07933E-23 |
|  | MBOAT2 | -0.424459888 | 7.53045E-23 |
|  | GCLC | -0.585703153 | 8.8963E-23 |
|  | SHPRH | -0.72440806 | 9.45483E-23 |
|  | C21orf34 | -0.628218426 | 9.50887E-23 |
|  | ZBTB26 | -0.599601448 | 9.87449E-23 |
|  | CLOCK | -0.782594646 | 1.11389E-22 |
|  | TJP1 | -0.412214927 | 1.13129E-22 |
|  | UEVLD | -0.495348479 | 1.19891E-22 |
|  | APOOL | -0.74214391 | 1.50274E-22 |
|  | RNF141 | -0.450992962 | 1.52603E-22 |
|  | ACBD5 | -0.503576118 | 1.53539E-22 |
|  | KIAA2018 | -0.457900122 | 1.56686E-22 |
|  | ZNF192 | -0.828106197 | 1.87542E-22 |
|  | ZNF660 | -0.717289128 | 1.97488E-22 |
|  | PTPRG | -0.551121447 | 1.97911E-22 |
|  | SORL1 | -0.693003622 | 2.03192E-22 |
|  | LRP1B | -0.99464464 | 2.09234E-22 |
|  | PIK3C2A | -0.480631676 | 2.18236E-22 |
|  | SESN3 | -0.878006154 | 2.18236E-22 |
|  | SPTLC2 | -0.519694181 | 2.72639E-22 |
|  | SETD7 | -0.455364113 | 3.02107E-22 |
|  | EIF2C4 | -0.504296984 | 3.19893E-22 |
|  | SDCCAG1 | -0.437935513 | 4.65727E-22 |
|  | PAFAH1B2 | -0.527355331 | 4.87204E-22 |
|  | EPB41L2 | -0.489439586 | 4.91327E-22 |
|  | RSF1 | -0.569749968 | 5.07463E-22 |
|  | KBTBD7 | -0.697433971 | 5.07463E-22 |
|  | TMLHE | -0.422244988 | 5.09477E-22 |
|  | CPEB4 | -0.482282099 | 5.13145E-22 |
|  | DOCK4 | -0.402918706 | 5.20999E-22 |
|  | PREX2 | -1.069813123 | 6.77054E-22 |
|  | VPS13C | -0.47156451 | 7.21034E-22 |
|  | FGD4 | -0.521375202 | 7.21034E-22 |
|  | SGMS1 | -0.536475684 | 7.23131E-22 |
|  | ELK4 | -0.544080547 | 8.19029E-22 |
|  | DHFRL1 | -0.430053481 | 9.53699E-22 |
|  | N4BP2 | -0.860679987 | 9.67801E-22 |
|  | NIPBL | -0.400152279 | 1.0044E-21 |
|  | ZNF778 | -0.621525428 | 1.02722E-21 |
|  | FADS1 | -0.51061161 | 1.04407E-21 |
|  | KLHL23 | -0.740379095 | 1.19004E-21 |
|  | MYST3 | -0.463844244 | 1.30489E-21 |
|  | ZC3H13 | -0.503010225 | 1.32855E-21 |
|  | SBF2 | -0.465975213 | 1.39951E-21 |
|  | UTP14C | -0.403667292 | 1.57541E-21 |
|  | KIAA1147 | -0.54194928 | 1.61138E-21 |
|  | PIK3CA | -0.517454512 | 1.68132E-21 |
|  | MYCBP2 | -0.661288055 | 2.02359E-21 |
|  | TET2 | -0.445507865 | 2.16559E-21 |
|  | PPTC7 | -0.560148358 | 2.21022E-21 |
|  | TRIP11 | -0.507798382 | 2.4417E-21 |
|  | ARHGAP5 | -0.481348442 | 2.45984E-21 |
|  | CLASP2 | -0.561675796 | 2.60775E-21 |
|  | MAPK8 | -0.645303456 | 3.11488E-21 |
|  | LIFR | -0.724000839 | 3.16374E-21 |
|  | CHD9 | -0.503680843 | 3.18146E-21 |
|  | GCC2 | -0.41954951 | 3.7483E-21 |
|  | DDX21 | -0.49206085 | 3.9494E-21 |
|  | MOBKL2B | -0.835932513 | 4.24343E-21 |
|  | NEU3 | -0.693461238 | 4.35324E-21 |
|  | ITPR2 | -0.602654096 | 4.90393E-21 |
|  | ALDH6A1 | -0.700551757 | 5.47124E-21 |
|  | ACADSB | -0.537857369 | 5.96282E-21 |
|  | AMOT | -0.853950506 | 6.49719E-21 |
|  | TMED10P1 | -0.545666192 | 7.81785E-21 |
|  | JMJD1C | -0.572181671 | 8.29817E-21 |
|  | ZNF281 | -0.60608045 | 8.29817E-21 |
|  | ATF7IP | -0.616784886 | 8.99511E-21 |
|  | ZEB2 | -0.491378512 | 9.37595E-21 |
|  | NEK7 | -0.423310921 | 1.01748E-20 |
|  | SPAG9 | -0.42299002 | 1.04258E-20 |
|  | CHST15 | -0.637333993 | 1.05868E-20 |
|  | MARCH1 | -0.687694001 | 1.25793E-20 |
|  | RNF150 | -0.934574711 | 1.40562E-20 |
|  | PTPRD | -0.83356402 | 1.47761E-20 |
|  | AKAP9 | -0.54660427 | 1.73404E-20 |
|  | LIMCH1 | -0.608769312 | 1.79095E-20 |
|  | SERINC5 | -1.121473271 | 1.84616E-20 |
|  | STOX2 | -0.892932394 | 1.8914E-20 |
|  | LCA5 | -0.46517487 | 1.97062E-20 |
|  | C14orf118 | -0.448531229 | 1.97847E-20 |
|  | P2RY12 | -1.09162581 | 2.06078E-20 |
|  | TRIM2 | -0.486588941 | 2.31045E-20 |
|  | SLC30A4 | -0.64380642 | 2.66351E-20 |
|  | VPS13A | -0.573870107 | 3.13792E-20 |
|  | STAG1 | -0.439914149 | 3.45277E-20 |
|  | LOC284441 | -0.600920838 | 3.51415E-20 |
|  | PDE3B | -0.611533966 | 3.61558E-20 |
|  | GLUD2 | -0.661255214 | 4.10438E-20 |
|  | CAMSAP1L1 | -0.431647474 | 4.33076E-20 |
|  | HIPK2 | -0.731090341 | 4.33844E-20 |
|  | MED13L | -0.535793323 | 4.33987E-20 |
|  | BRWD3 | -0.819551379 | 4.36628E-20 |
|  | PPP4R2 | -0.456970518 | 4.40337E-20 |
|  | RICTOR | -0.670762559 | 5.15094E-20 |
|  | ZMYND11 | -0.514611538 | 5.71958E-20 |
|  | MAP9 | -0.49333777 | 6.01151E-20 |
|  | ARID4A | -0.430197855 | 6.10074E-20 |
|  | DENND5A | -0.429823239 | 6.17818E-20 |
|  | USP46 | -0.517482641 | 6.34359E-20 |
|  | MSI2 | -0.443075663 | 6.61775E-20 |
|  | HERC3 | -0.44443728 | 7.35687E-20 |
|  | HEATR5A | -0.533008795 | 7.42187E-20 |
|  | RAPGEF2 | -0.492125923 | 9.05911E-20 |
|  | MARCH8 | -0.891097161 | 9.09978E-20 |
|  | APC | -0.484840247 | 9.34595E-20 |
|  | MARCH6 | -0.438294505 | 1.01009E-19 |
|  | TACC1 | -0.417248327 | 1.01278E-19 |
|  | LASS6 | -0.491239709 | 1.02096E-19 |
|  | VPS13D | -0.695246456 | 1.07314E-19 |
|  | MIB1 | -0.454522742 | 1.09937E-19 |
|  | DAAM2 | -1.273182046 | 1.22137E-19 |
|  | IDE | -0.464680938 | 1.29586E-19 |
|  | BAZ2B | -0.483705421 | 1.42338E-19 |
|  | FAM59A | -0.563873964 | 1.45346E-19 |
|  | GTF3C4 | -0.536890639 | 1.45963E-19 |
|  | EP300 | -0.491852169 | 1.49528E-19 |
|  | BOD1L | -0.448624374 | 1.55899E-19 |
|  | ATM | -0.433066108 | 1.83359E-19 |
|  | IKZF2 | -0.47451413 | 1.87357E-19 |
|  | QKI | -0.432478075 | 1.90683E-19 |
|  | KIAA1958 | -0.6506923 | 1.96189E-19 |
|  | BDP1 | -0.444789572 | 2.04952E-19 |
|  | EPC1 | -0.556173332 | 2.17224E-19 |
|  | MOSPD2 | -0.438904561 | 2.17733E-19 |
|  | C6orf204 | -0.787340455 | 2.50461E-19 |
|  | MAML2 | -0.755563281 | 2.67116E-19 |
|  | NRXN1 | -1.044933346 | 3.04E-19 |
|  | DIP2B | -0.441650828 | 3.09169E-19 |
|  | P2RX7 | -0.842520082 | 3.24638E-19 |
|  | TMEM170B | -0.469920073 | 4.55687E-19 |
|  | CALCRL | -1.001673361 | 4.56289E-19 |
|  | PTPN11 | -0.417306599 | 4.82495E-19 |
|  | NTRK2 | -1.025960812 | 6.16497E-19 |
|  | ATP9A | -0.664687267 | 6.62772E-19 |
|  | EIF2AK2 | -0.513773725 | 6.93361E-19 |
|  | PLCL1 | -0.840307833 | 6.95506E-19 |
|  | NR1D2 | -0.510715329 | 7.59997E-19 |
|  | SMG1 | -0.422462031 | 7.86854E-19 |
|  | KCTD20 | -0.404474248 | 8.38989E-19 |
|  | SLC12A6 | -0.458305759 | 8.95644E-19 |
|  | KIF27 | -0.553793882 | 9.09109E-19 |
|  | SORBS1 | -0.58492158 | 9.24259E-19 |
|  | FAM73A | -0.544746963 | 9.42691E-19 |
|  | PBLD | -0.527270872 | 1.00606E-18 |
|  | NALCN | -0.964442878 | 1.20993E-18 |
|  | WNK3 | -0.826205354 | 1.26307E-18 |
|  | PIK3R1 | -0.596476344 | 1.28904E-18 |
|  | PTENP1 | -0.40922886 | 1.35713E-18 |
|  | CHD6 | -0.467954712 | 1.38729E-18 |
|  | MBD5 | -0.403188106 | 1.51192E-18 |
|  | AKAP6 | -0.754769733 | 1.54847E-18 |
|  | DYNC2H1 | -0.540146733 | 1.57222E-18 |
|  | KIAA1244 | -1.230099423 | 1.72272E-18 |
|  | C8orf37 | -0.463808969 | 1.7713E-18 |
|  | WNK1 | -0.402619964 | 1.83601E-18 |
|  | ARHGAP31 | -0.545105312 | 1.86664E-18 |
|  | LANCL1 | -0.417301593 | 2.01017E-18 |
|  | LRCH2 | -0.516822998 | 2.21095E-18 |
|  | FGF2 | -0.484129312 | 2.57481E-18 |
|  | ABLIM1 | -0.623369095 | 2.61512E-18 |
|  | ARHGAP42 | -0.558588325 | 2.78931E-18 |
|  | MYST4 | -0.623078429 | 2.83821E-18 |
|  | SATB1 | -0.728591534 | 3.41139E-18 |
|  | FAM123A | -0.940959963 | 3.56833E-18 |
|  | NLGN1 | -0.494737401 | 4.31052E-18 |
|  | SIRPB1 | -0.693761503 | 5.3996E-18 |
|  | ST6GALNAC3 | -0.570130437 | 5.44786E-18 |
|  | DICER1 | -0.406403316 | 5.69488E-18 |
|  | PDE4DIP | -0.566105035 | 5.9878E-18 |
|  | PXK | -0.438710648 | 6.24136E-18 |
|  | JMY | -0.437660655 | 7.18787E-18 |
|  | NEK1 | -0.405302363 | 7.44352E-18 |
|  | SLC23A2 | -0.416161682 | 7.57697E-18 |
|  | PRRG1 | -0.617762859 | 8.20879E-18 |
|  | NEBL | -0.827700281 | 8.46442E-18 |
|  | PDPR | -0.606346112 | 8.47028E-18 |
|  | ZNF430 | -0.474086899 | 9.07479E-18 |
|  | GPM6B | -0.499830727 | 1.27267E-17 |
|  | FAM190B | -0.524698591 | 1.42262E-17 |
|  | TUBGCP4 | -0.441105656 | 1.49893E-17 |
|  | PTPN4 | -0.495675558 | 1.59364E-17 |
|  | FRS2 | -0.408142598 | 1.6424E-17 |
|  | SBNO1 | -0.601846349 | 1.77349E-17 |
|  | SASH1 | -0.564595092 | 1.86652E-17 |
|  | ZNF292 | -0.480675677 | 1.87094E-17 |
|  | PLCB1 | -0.895847311 | 1.89451E-17 |
|  | KIAA1370 | -0.423319704 | 1.95349E-17 |
|  | PPP2R3A | -0.53572628 | 1.99903E-17 |
|  | TRIM44 | -0.482936923 | 2.01307E-17 |
|  | THSD7A | -0.968411109 | 2.07659E-17 |
|  | KLF12 | -0.539733947 | 2.41393E-17 |
|  | GRAMD1B | -0.917861597 | 2.45389E-17 |
|  | FAM55C | -0.437103148 | 2.66701E-17 |
|  | PHF16 | -0.643365498 | 2.73946E-17 |
|  | SOX2OT | -0.535988841 | 2.85157E-17 |
|  | FAM167A | -0.868687969 | 2.8802E-17 |
|  | RGP1 | -0.555477374 | 2.95056E-17 |
|  | KIAA0564 | -0.42639827 | 3.15043E-17 |
|  | ICK | -0.476717836 | 3.28766E-17 |
|  | AKD1 | -0.48250283 | 3.37849E-17 |
|  | ITSN1 | -0.430234099 | 3.50481E-17 |
|  | C10orf12 | -0.589770276 | 3.55597E-17 |
|  | CAMK1D | -0.617970444 | 3.60461E-17 |
|  | RFTN2 | -0.580553292 | 3.84332E-17 |
|  | H3F3C | -0.60686417 | 3.9437E-17 |
|  | MLL | -0.568406549 | 3.9803E-17 |
|  | DGKI | -0.891486226 | 3.98298E-17 |
|  | SGK269 | -0.46960315 | 4.13317E-17 |
|  | PPP1R12B | -0.519271638 | 4.32527E-17 |
|  | SLC15A2 | -0.831103574 | 4.472E-17 |
|  | REPS2 | -1.134778979 | 4.49209E-17 |
|  | TJP2 | -0.820679126 | 4.65027E-17 |
|  | DST | -0.648115878 | 4.7752E-17 |
|  | PAIP2B | -0.955223052 | 4.8528E-17 |
|  | RALGPS2 | -0.683734025 | 5.04986E-17 |
|  | DHTKD1 | -0.474945834 | 5.15065E-17 |
|  | FRY | -0.700152557 | 5.5816E-17 |
|  | CELF2 | -0.443491723 | 5.90741E-17 |
|  | MDN1 | -0.667349538 | 5.99765E-17 |
|  | FIGN | -0.664694778 | 6.11468E-17 |
|  | KIAA1211 | -0.623352536 | 6.19375E-17 |
|  | BRAF | -0.522470171 | 6.32796E-17 |
|  | GPRIN3 | -0.735426708 | 6.37749E-17 |
|  | SLC9A6 | -0.420637078 | 6.53685E-17 |
|  | GPRC5B | -0.524109004 | 6.68202E-17 |
|  | ZBTB10 | -0.448007022 | 6.85365E-17 |
|  | BCL2 | -0.483270099 | 7.3261E-17 |
|  | BMPR1A | -0.403624481 | 7.49954E-17 |
|  | PDLIM5 | -0.526628071 | 7.69911E-17 |
|  | MLL3 | -0.44295927 | 9.05277E-17 |
|  | SIRT1 | -0.430053374 | 9.26074E-17 |
|  | PLEKHM3 | -0.538054505 | 9.83092E-17 |
|  | SSPN | -0.600381384 | 9.92486E-17 |
|  | CCDC160 | -0.676563063 | 9.97965E-17 |
|  | NAV3 | -0.780403469 | 1.04326E-16 |
|  | ZXDA | -0.503523836 | 1.13007E-16 |
|  | JHDM1D | -0.479975919 | 1.16098E-16 |
|  | CADM2 | -0.839168661 | 1.19313E-16 |
|  | ZNF609 | -0.451768251 | 1.26497E-16 |
|  | ASB1 | -0.41549052 | 1.32048E-16 |
|  | PRKCA | -0.501622996 | 1.48403E-16 |
|  | C14orf37 | -0.505789359 | 1.64616E-16 |
|  | ANTXR1 | -0.426290854 | 1.69668E-16 |
|  | PPM1K | -0.538052843 | 1.77552E-16 |
|  | ADRBK2 | -0.586443853 | 1.89216E-16 |
|  | ZBTB20 | -0.85212983 | 1.92998E-16 |
|  | EDIL3 | -1.121342445 | 1.95135E-16 |
|  | PAQR8 | -0.670763505 | 1.97286E-16 |
|  | MEGF9 | -0.429212934 | 1.99515E-16 |
|  | FAT3 | -0.739888704 | 2.37487E-16 |
|  | CPEB3 | -0.792123203 | 2.41287E-16 |
|  | TXLNG | -0.524789603 | 2.54777E-16 |
|  | WWC2 | -0.405530869 | 2.64918E-16 |
|  | ZNF704 | -0.743647917 | 2.67372E-16 |
|  | KIAA0754 | -0.953126506 | 2.67372E-16 |
|  | BPTF | -0.40622882 | 2.75264E-16 |
|  | WASF3 | -0.628039398 | 2.75327E-16 |
|  | TIAM1 | -0.457458079 | 2.78559E-16 |
|  | SMC3 | -0.451254693 | 2.9962E-16 |
|  | BTAF1 | -0.49938209 | 3.08764E-16 |
|  | GOLIM4 | -0.573399051 | 3.20466E-16 |
|  | MYSM1 | -0.675281969 | 3.43698E-16 |
|  | C14orf21 | -0.499540751 | 3.9528E-16 |
|  | DZIP3 | -0.419604035 | 4.298E-16 |
|  | CMTM4 | -0.597048184 | 4.31206E-16 |
|  | RASAL2 | -0.425635502 | 4.37145E-16 |
|  | WDR17 | -0.57500416 | 5.29122E-16 |
|  | C10orf18 | -0.411055276 | 5.39598E-16 |
|  | KCNJ10 | -0.708989573 | 5.68036E-16 |
|  | KIAA1671 | -0.627248936 | 5.74199E-16 |
|  | ZRANB1 | -0.433147149 | 5.75872E-16 |
|  | ZDHHC2 | -0.496627037 | 5.89805E-16 |
|  | ROCK2 | -0.503985003 | 6.0095E-16 |
|  | ANKRD46 | -0.409459054 | 6.27184E-16 |
|  | LPP | -0.614034227 | 6.46349E-16 |
|  | ETV3 | -0.610011184 | 6.85732E-16 |
|  | AMOTL1 | -0.424251786 | 7.51102E-16 |
|  | ZBED3 | -0.627360847 | 8.08983E-16 |
|  | PHYHIPL | -0.798414279 | 8.11131E-16 |
|  | RALGAPA1 | -0.428186556 | 8.39929E-16 |
|  | MXI1 | -0.501196427 | 9.06009E-16 |
|  | USP54 | -0.690123216 | 9.29606E-16 |
|  | KIF13A | -0.599744104 | 9.76557E-16 |
|  | CDH20 | -0.895543488 | 1.00568E-15 |
|  | PEA15 | -0.509536359 | 1.03039E-15 |
|  | DENND1B | -0.494168841 | 1.04022E-15 |
|  | NCKAP5 | -0.543109822 | 1.14945E-15 |
|  | RAD54L2 | -0.605128698 | 1.32155E-15 |
|  | SPATA13 | -0.685303331 | 1.40502E-15 |
|  | LOC729176 | -0.410664862 | 1.40858E-15 |
|  | SCAPER | -0.456791285 | 1.43908E-15 |
|  | KIAA1161 | -0.748233344 | 1.57773E-15 |
|  | PARD3 | -0.498155879 | 1.61427E-15 |
|  | FAM115A | -0.694101911 | 1.63954E-15 |
|  | ATP8A1 | -0.878987433 | 1.75284E-15 |
|  | ZBTB16 | -1.029897774 | 1.79754E-15 |
|  | ZNF717 | -0.530584819 | 1.79946E-15 |
|  | FMO5 | -0.525499473 | 1.85959E-15 |
|  | FAM161B | -0.500890159 | 1.8942E-15 |
|  | ZNF391 | -0.528456833 | 1.95864E-15 |
|  | GPAM | -0.459569732 | 2.08331E-15 |
|  | ALDH5A1 | -0.59063992 | 2.12532E-15 |
|  | ZNF275 | -0.453036558 | 2.13645E-15 |
|  | PCYT1B | -0.527029809 | 2.18838E-15 |
|  | DOCK10 | -0.543860084 | 2.30342E-15 |
|  | SCD | -0.777325249 | 2.31987E-15 |
|  | C9orf93 | -0.592606974 | 2.40475E-15 |
|  | MTMR7 | -0.88091942 | 2.47049E-15 |
|  | ALCAM | -0.590071994 | 2.548E-15 |
|  | FAM13C | -0.754343293 | 2.58174E-15 |
|  | RGPD3 | -0.426963956 | 2.58868E-15 |
|  | SELL | -1.423238165 | 2.59184E-15 |
|  | PDE8B | -0.793438286 | 2.71314E-15 |
|  | PRICKLE2 | -0.416073556 | 2.71526E-15 |
|  | ZNF827 | -0.657355467 | 2.90829E-15 |
|  | OPHN1 | -0.764095926 | 2.93471E-15 |
|  | DOCK1 | -0.420868096 | 3.19337E-15 |
|  | DSTYK | -0.409230851 | 3.38375E-15 |
|  | KAL1 | -0.702425136 | 3.4071E-15 |
|  | CPE | -0.656913326 | 3.77945E-15 |
|  | SLCO1A2 | -1.177566112 | 3.77945E-15 |
|  | RSPH3 | -0.435765707 | 3.91962E-15 |
|  | PLCL2 | -0.456585367 | 4.243E-15 |
|  | HERC1 | -0.469680927 | 4.63666E-15 |
|  | SYNPO2 | -0.772194989 | 4.96442E-15 |
|  | ZNF774 | -0.455727401 | 5.01172E-15 |
|  | PVRL3 | -0.634670566 | 5.47412E-15 |
|  | B3GAT2 | -1.050545822 | 5.82949E-15 |
|  | MMP16 | -0.782786549 | 5.83904E-15 |
|  | RAB11FIP2 | -0.425642647 | 5.9456E-15 |
|  | MFSD6 | -0.527897787 | 6.27318E-15 |
|  | ZNF33A | -0.405568763 | 6.47234E-15 |
|  | ENPP4 | -0.646285567 | 6.47827E-15 |
|  | SGCD | -1.118846403 | 6.66868E-15 |
|  | KIF1B | -0.481016918 | 6.90866E-15 |
|  | TMOD2 | -0.600194135 | 7.36183E-15 |
|  | ABI3BP | -1.131692189 | 7.43628E-15 |
|  | DENND5B | -0.454962701 | 7.53836E-15 |
|  | RNF168 | -0.479885756 | 7.6554E-15 |
|  | OSGIN2 | -0.550416565 | 7.82289E-15 |
|  | HECW2 | -0.581134303 | 7.96691E-15 |
|  | SYNE1 | -0.570966071 | 8.2506E-15 |
|  | AGXT2L1 | -1.538348835 | 8.79393E-15 |
|  | C20orf194 | -0.431786203 | 9.10622E-15 |
|  | GUCY1A3 | -0.776473791 | 9.43488E-15 |
|  | SLC16A9 | -0.619924492 | 9.74327E-15 |
|  | ADIPOR2 | -0.401727577 | 1.13313E-14 |
|  | DMXL2 | -0.414987027 | 1.13457E-14 |
|  | PDK4 | -0.882614747 | 1.30928E-14 |
|  | SPARCL1 | -0.644966303 | 1.33902E-14 |
|  | DPY19L3 | -0.519738102 | 1.39295E-14 |
|  | LRP6 | -0.430171732 | 1.43495E-14 |
|  | BAG4 | -0.494651042 | 1.66778E-14 |
|  | MRO | -0.807610962 | 1.67711E-14 |
|  | DCLK1 | -0.670581126 | 1.72948E-14 |
|  | AHNAK | -0.74447833 | 2.04111E-14 |
|  | TGFBR3 | -0.629478847 | 2.06212E-14 |
|  | SLC8A1 | -0.404647024 | 2.14787E-14 |
|  | GDPD1 | -0.547677165 | 2.30515E-14 |
|  | ARRB1 | -0.630968798 | 2.31771E-14 |
|  | MAST4 | -0.610266581 | 2.35568E-14 |
|  | KIAA1107 | -0.720750347 | 2.44073E-14 |
|  | RNF125 | -0.539965563 | 2.48581E-14 |
|  | MYO10 | -0.44291587 | 2.51034E-14 |
|  | TUB | -0.703935064 | 2.67018E-14 |
|  | AVL9 | -0.457576296 | 2.72735E-14 |
|  | REEP3 | -0.42357976 | 3.10824E-14 |
|  | IL6R | -0.574246037 | 3.65823E-14 |
|  | RALGAPA2 | -0.568182603 | 3.68324E-14 |
|  | FOXO3B | -0.429218764 | 4.05546E-14 |
|  | ERBB4 | -0.928880709 | 4.159E-14 |
|  | ZNF462 | -0.561538218 | 4.51112E-14 |
|  | C7orf41 | -0.755346521 | 4.76556E-14 |
|  | SOX5 | -0.80449561 | 5.38397E-14 |
|  | FAM107B | -0.532808379 | 5.57858E-14 |
|  | MAPK10 | -0.471762007 | 5.58428E-14 |
|  | KIAA1324L | -0.592657507 | 5.75015E-14 |
|  | NET1 | -0.71627688 | 5.7539E-14 |
|  | UTRN | -0.480886616 | 5.82297E-14 |
|  | NBEA | -0.624420333 | 5.8254E-14 |
|  | ELOVL7 | -0.593288766 | 5.88807E-14 |
|  | RFX7 | -0.401107512 | 6.35947E-14 |
|  | GRAMD3 | -0.493798568 | 6.55197E-14 |
|  | DEPDC6 | -0.547535248 | 6.88694E-14 |
|  | ZNF844 | -0.413651267 | 6.91614E-14 |
|  | CAMK2G | -0.550536436 | 6.96094E-14 |
|  | PLCXD2 | -0.848457418 | 7.36513E-14 |
|  | PLXNC1 | -0.708778681 | 7.46339E-14 |
|  | PRKX | -0.58504547 | 7.54933E-14 |
|  | SPIRE1 | -0.4032542 | 9.2684E-14 |
|  | DLG2 | -0.760144617 | 9.59738E-14 |
|  | MRVI1 | -0.736825936 | 1.00447E-13 |
|  | DOPEY1 | -0.415595461 | 1.0113E-13 |
|  | LRRC8B | -0.442629784 | 1.05118E-13 |
|  | ZNF426 | -0.414908476 | 1.09025E-13 |
|  | AKT3 | -0.473047506 | 1.16738E-13 |
|  | NAP1L2 | -0.851455101 | 1.17185E-13 |
|  | AKR1C3 | -0.910766326 | 1.17333E-13 |
|  | C14orf132 | -0.701166797 | 1.19993E-13 |
|  | NCAM2 | -0.678390872 | 1.24841E-13 |
|  | PGBD4 | -0.422355583 | 1.38368E-13 |
|  | C2orf88 | -0.690080785 | 1.44133E-13 |
|  | HPCAL4 | -1.136434632 | 1.46089E-13 |
|  | SLC38A1 | -0.821282061 | 1.52726E-13 |
|  | AHCYL1 | -0.587984887 | 1.63291E-13 |
|  | CACNB4 | -0.561583523 | 1.67221E-13 |
|  | ARHGEF6 | -0.408829314 | 1.67512E-13 |
|  | MAP3K5 | -0.52876662 | 1.7685E-13 |
|  | GLUD1 | -0.515041363 | 1.79807E-13 |
|  | PSAT1 | -0.467723158 | 1.93974E-13 |
|  | SIDT1 | -0.861265319 | 1.94655E-13 |
|  | PIK3IP1 | -0.433305532 | 2.20894E-13 |
|  | ABCG1 | -0.47915393 | 2.26074E-13 |
|  | UPF2 | -0.420320061 | 2.266E-13 |
|  | ZEB1 | -0.485377326 | 2.3657E-13 |
|  | OMG | -0.746776272 | 2.58186E-13 |
|  | LRIG1 | -0.596073508 | 2.60827E-13 |
|  | REV3L | -0.469809628 | 2.65774E-13 |
|  | LOC150622 | -0.945012913 | 2.67877E-13 |
|  | LOC646214 | -0.521216358 | 2.82104E-13 |
|  | DHX33 | -0.43607779 | 3.01236E-13 |
|  | SALL2 | -0.447748368 | 3.28099E-13 |
|  | C1orf198 | -0.465058682 | 3.4588E-13 |
|  | KLHDC10 | -0.441504236 | 3.61445E-13 |
|  | DIP2C | -0.450489529 | 3.73801E-13 |
|  | PHACTR1 | -0.703126509 | 3.82769E-13 |
|  | NAP1L3 | -0.602385151 | 3.86897E-13 |
|  | ANKRD26 | -0.542691804 | 4.12506E-13 |
|  | STXBP5 | -0.61756479 | 4.2704E-13 |
|  | FCHSD2 | -0.465602546 | 5.17831E-13 |
|  | ZNF813 | -0.44286394 | 5.18143E-13 |
|  | PCDH17 | -0.432474426 | 5.78519E-13 |
|  | ZNF573 | -0.520929534 | 6.75488E-13 |
|  | LPIN1 | -0.41179346 | 7.0268E-13 |
|  | TGFBRAP1 | -0.472438926 | 7.30798E-13 |
|  | ZNF710 | -0.402150902 | 8.13195E-13 |
|  | SLC1A4 | -0.598602981 | 8.88804E-13 |
|  | STON1 | -0.704528711 | 9.03934E-13 |
|  | KLHL9 | -0.490967327 | 9.28933E-13 |
|  | GNAL | -1.150148694 | 9.65569E-13 |
|  | LPHN3 | -0.535877045 | 1.17848E-12 |
|  | RGPD1 | -0.465872114 | 1.27569E-12 |
|  | KIF3A | -0.475603751 | 1.33599E-12 |
|  | PELI2 | -0.480914441 | 1.34255E-12 |
|  | MCART6 | -0.558768221 | 1.45588E-12 |
|  | SCN8A | -0.876457436 | 1.7058E-12 |
|  | GGTA1 | -0.799959502 | 1.87488E-12 |
|  | SPARC | -0.546330357 | 1.95432E-12 |
|  | SLC4A4 | -0.691856851 | 2.18529E-12 |
|  | ARHGAP32 | -0.477417062 | 2.32673E-12 |
|  | DOCK5 | -0.906137551 | 2.3735E-12 |
|  | RASGEF1B | -0.529576403 | 2.37549E-12 |
|  | CCDC85A | -0.877537671 | 2.52825E-12 |
|  | PPP2R5A | -0.457065755 | 2.54885E-12 |
|  | HNMT | -0.428519785 | 2.59803E-12 |
|  | PTPRB | -0.549489717 | 2.6542E-12 |
|  | RORA | -0.42511553 | 2.68898E-12 |
|  | PCDH7 | -0.882708429 | 2.77861E-12 |
|  | TET1 | -0.724061751 | 2.96452E-12 |
|  | LMTK2 | -0.633703513 | 3.06745E-12 |
|  | FAT1 | -0.544113424 | 3.11225E-12 |
|  | DNAJC6 | -0.607666756 | 3.13135E-12 |
|  | PRKAR2A | -0.474413821 | 3.26669E-12 |
|  | TTLL7 | -0.616081996 | 3.4566E-12 |
|  | TAF3 | -0.454899473 | 3.52079E-12 |
|  | PRTG | -0.632638761 | 3.56921E-12 |
|  | NR3C2 | -0.598185966 | 3.59114E-12 |
|  | FAM124A | -0.537826801 | 3.77879E-12 |
|  | FGD6 | -0.435320353 | 3.80076E-12 |
|  | ZDBF2 | -0.661920532 | 3.85773E-12 |
|  | KCND2 | -0.887835062 | 3.87564E-12 |
|  | TMEM100 | -0.990756367 | 4.10222E-12 |
|  | PLP1 | -0.90863167 | 4.11815E-12 |
|  | ARAP2 | -0.526259655 | 4.15654E-12 |
|  | NAV1 | -0.471084216 | 4.27629E-12 |
|  | ADCY2 | -0.72882717 | 4.35953E-12 |
|  | ABAT | -0.51726316 | 4.4892E-12 |
|  | DOCK9 | -0.523833352 | 4.77076E-12 |
|  | DLGAP1 | -0.882533027 | 5.12365E-12 |
|  | SLC7A2 | -0.777256118 | 5.26867E-12 |
|  | SORCS3 | -0.843915571 | 5.34531E-12 |
|  | DIAPH2 | -0.454791782 | 6.62441E-12 |
|  | HMGN5 | -0.591077687 | 6.89107E-12 |
|  | TTYH2 | -0.473218963 | 7.4961E-12 |
|  | GRIA1 | -0.956077775 | 7.74165E-12 |
|  | CAB39L | -0.46815401 | 7.76698E-12 |
|  | SSTR2 | -0.985549114 | 7.82014E-12 |
|  | ABCC9 | -0.669578929 | 7.82354E-12 |
|  | MPP6 | -0.59786931 | 8.38846E-12 |
|  | OTUD1 | -0.491990652 | 9.30389E-12 |
|  | ATRNL1 | -0.838792928 | 1.04175E-11 |
|  | FAM69A | -0.442860021 | 1.23057E-11 |
|  | CD84 | -0.740484932 | 1.33726E-11 |
|  | ARL10 | -0.503235505 | 1.3487E-11 |
|  | FAM49A | -0.459940537 | 1.35005E-11 |
|  | GPR98 | -0.923459048 | 1.36691E-11 |
|  | CNTF | -0.574281047 | 1.59005E-11 |
|  | NDRG2 | -0.667404149 | 1.64894E-11 |
|  | MAP2 | -0.615559144 | 1.65058E-11 |
|  | KIF5A | -0.938459879 | 1.75987E-11 |
|  | NCAM1 | -0.500446782 | 1.76495E-11 |
|  | KBTBD11 | -0.600293092 | 1.77267E-11 |
|  | TMEM56 | -0.67778645 | 1.77303E-11 |
|  | RAB6B | -0.500381776 | 1.78253E-11 |
|  | SH3BGRL2 | -0.451192942 | 1.86302E-11 |
|  | MAGI3 | -0.434404858 | 1.89155E-11 |
|  | C12orf51 | -0.432481345 | 1.99748E-11 |
|  | DAAM1 | -0.44636201 | 2.07837E-11 |
|  | PAR-SN | -0.512236858 | 2.1013E-11 |
|  | CPEB1 | -0.541649071 | 2.17271E-11 |
|  | GUSBL1 | -0.665333237 | 2.38036E-11 |
|  | TTN | -0.59329612 | 2.41012E-11 |
|  | SDPR | -0.750706815 | 2.4446E-11 |
|  | SNORD116-28 | -0.614072895 | 2.5573E-11 |
|  | MASP1 | -0.693339325 | 2.63884E-11 |
|  | ZNF280B | -0.402820657 | 2.69936E-11 |
|  | EPHB1 | -0.850960843 | 2.88435E-11 |
|  | EIF2C2 | -0.455857362 | 2.88442E-11 |
|  | ZMAT3 | -0.49478375 | 3.03605E-11 |
|  | DNAH7 | -0.743275218 | 3.57729E-11 |
|  | MGC16275 | -0.408475933 | 3.7624E-11 |
|  | SACS | -0.401571075 | 4.03101E-11 |
|  | KANK1 | -0.406146534 | 4.11118E-11 |
|  | ALDH2 | -0.487912875 | 4.11596E-11 |
|  | MAP3K13 | -0.517467295 | 4.266E-11 |
|  | SPON1 | -0.869449276 | 4.40659E-11 |
|  | SYT6 | -0.710723639 | 4.43961E-11 |
|  | APCDD1 | -0.400901052 | 4.53947E-11 |
|  | DGKB | -0.782286793 | 4.76871E-11 |
|  | DTNA | -0.405905162 | 4.80502E-11 |
|  | sept-03 | -0.610666469 | 4.91918E-11 |
|  | SOX6 | -0.628060583 | 5.00362E-11 |
|  | SLC9A7 | -0.64370065 | 5.05435E-11 |
|  | NTM | -0.541247251 | 5.17601E-11 |
|  | ANKS1B | -0.778817467 | 5.21861E-11 |
|  | TLR3 | -0.542648797 | 5.32887E-11 |
|  | HMGCR | -0.417129831 | 5.38936E-11 |
|  | DNM3 | -0.718854451 | 5.54251E-11 |
|  | TTC3 | -0.431496218 | 5.70264E-11 |
|  | FADS2 | -0.450299885 | 6.18859E-11 |
|  | LRRC37A4 | -0.73552046 | 6.32321E-11 |
|  | SCD5 | -0.458730071 | 6.53471E-11 |
|  | RAB6C | -0.48382652 | 6.68983E-11 |
|  | ALDH1L2 | -0.571765898 | 6.75841E-11 |
|  | HDAC4 | -0.482635298 | 6.88822E-11 |
|  | S1PR1 | -0.485596702 | 6.92521E-11 |
|  | NBPF10 | -0.556458111 | 7.1054E-11 |
|  | IFIT2 | -0.467668177 | 7.30984E-11 |
|  | DAPK1 | -0.488231934 | 7.82256E-11 |
|  | GUCY1A2 | -0.61149634 | 8.4125E-11 |
|  | KIF21B | -0.739141471 | 8.7713E-11 |
|  | C21orf91 | -0.576571645 | 9.12664E-11 |
|  | ATP1A2 | -0.818466399 | 9.75175E-11 |
|  | ANK2 | -0.40635779 | 1.02207E-10 |
|  | SYN3 | -0.84817254 | 1.17308E-10 |
|  | IPCEF1 | -0.776131586 | 1.26198E-10 |
|  | LIMS1 | -0.442923044 | 1.27051E-10 |
|  | SLC7A11 | -0.526538791 | 1.27909E-10 |
|  | ZMIZ1 | -0.4052938 | 1.29652E-10 |
|  | MERTK | -0.404375121 | 1.3827E-10 |
|  | RAP2A | -0.45399467 | 1.42377E-10 |
|  | HIVEP2 | -0.436200915 | 1.43209E-10 |
|  | DOK6 | -0.990301554 | 1.45275E-10 |
|  | LOC100271836 | -0.427472269 | 1.54401E-10 |
|  | GPR155 | -0.402580938 | 1.69744E-10 |
|  | PAQR5 | -0.429648558 | 1.69854E-10 |
|  | FAT4 | -0.561147736 | 1.73461E-10 |
|  | C14orf49 | -0.585060997 | 1.7348E-10 |
|  | CTTNBP2 | -0.607045677 | 1.77142E-10 |
|  | PLEKHA5 | -0.566921228 | 1.77973E-10 |
|  | PRDM5 | -0.436612076 | 1.89868E-10 |
|  | ADAMTSL3 | -0.741103177 | 2.13022E-10 |
|  | HMGCS1 | -0.484133099 | 2.29106E-10 |
|  | FAM171A1 | -0.487249582 | 2.33738E-10 |
|  | MAN2A1 | -0.480081383 | 2.41149E-10 |
|  | TECTA | -0.454167407 | 2.59429E-10 |
|  | TAGAP | -0.537206403 | 2.63853E-10 |
|  | TCF12 | -0.501528897 | 2.77257E-10 |
|  | SEZ6L | -1.018797089 | 2.8594E-10 |
|  | DDR2 | -0.839793657 | 2.88604E-10 |
|  | LRRC8A | -0.446357608 | 2.88885E-10 |
|  | AFF3 | -0.585650921 | 3.02873E-10 |
|  | IRAK3 | -0.424452887 | 3.15926E-10 |
|  | KIAA1598 | -0.560590459 | 3.39178E-10 |
|  | ACVR2B | -0.503506372 | 3.41366E-10 |
|  | ZNF711 | -0.44944567 | 3.43994E-10 |
|  | KIAA1549 | -0.454948809 | 3.47989E-10 |
|  | NFASC | -0.47330411 | 3.54435E-10 |
|  | HSPB8 | -0.723781108 | 3.6935E-10 |
|  | PER3 | -0.570917016 | 3.75102E-10 |
|  | RYR1 | -0.744184696 | 3.8506E-10 |
|  | SMOC1 | -1.292796111 | 4.2057E-10 |
|  | C8orf46 | -0.476071937 | 4.29845E-10 |
|  | KCNT2 | -0.607921915 | 4.43056E-10 |
|  | ATP10B | -0.793836852 | 4.91452E-10 |
|  | IGSF9B | -0.815305819 | 4.9265E-10 |
|  | LEPROT | -0.40034365 | 5.01301E-10 |
|  | KCNH1 | -0.907143096 | 5.39193E-10 |
|  | SGIP1 | -0.516878604 | 5.52377E-10 |
|  | EPB41L3 | -0.582414307 | 5.75452E-10 |
|  | FOXO4 | -0.440714718 | 7.00505E-10 |
|  | GNAI1 | -0.539653717 | 7.63623E-10 |
|  | C11orf41 | -0.590686956 | 7.76707E-10 |
|  | PRUNE2 | -0.45272076 | 7.97605E-10 |
|  | ALDOC | -0.706999707 | 8.06161E-10 |
|  | REST | -0.550389027 | 8.47126E-10 |
|  | KIF5C | -0.549678032 | 8.9458E-10 |
|  | EDNRB | -0.65887157 | 1.05776E-09 |
|  | SYBU | -0.46222314 | 1.14708E-09 |
|  | PRDM11 | -0.441415763 | 1.19694E-09 |
|  | ANK3 | -0.794519323 | 1.22784E-09 |
|  | MYO5A | -0.456479867 | 1.23556E-09 |
|  | ATP6V1G2 | -0.664967163 | 1.26599E-09 |
|  | PIP4K2A | -0.484937994 | 1.31833E-09 |
|  | GRIN3A | -1.071155545 | 1.33861E-09 |
|  | GABRB3 | -1.064184762 | 1.37884E-09 |
|  | FERMT1 | -1.015313051 | 1.39541E-09 |
|  | STXBP6 | -0.730963909 | 1.43347E-09 |
|  | LONRF3 | -0.593793069 | 1.43478E-09 |
|  | KIAA1409 | -0.873537033 | 1.54731E-09 |
|  | GPR173 | -0.424144415 | 1.58756E-09 |
|  | HLF | -0.657723535 | 1.62712E-09 |
|  | BEST1 | -0.522532229 | 1.75533E-09 |
|  | TAL1 | -0.438604056 | 1.87478E-09 |
|  | ST3GAL6 | -0.426001202 | 1.92796E-09 |
|  | GRIK4 | -0.467385551 | 2.1731E-09 |
|  | TCEAL2 | -0.629245278 | 2.24683E-09 |
|  | NT5DC1 | -0.437834634 | 2.28715E-09 |
|  | ZNF804A | -0.782924595 | 2.36661E-09 |
|  | MYRIP | -0.548394956 | 2.43937E-09 |
|  | SCN3A | -0.713803368 | 2.46807E-09 |
|  | ZNF488 | -0.995832476 | 2.58309E-09 |
|  | IL33 | -0.519084852 | 2.59388E-09 |
|  | GVIN1 | -0.464218514 | 2.61938E-09 |
|  | RND2 | -0.480682289 | 2.63274E-09 |
|  | SLC6A1 | -0.598349226 | 2.73985E-09 |
|  | FAM155A | -0.897433055 | 2.82481E-09 |
|  | PPP1R16B | -0.733925139 | 3.06557E-09 |
|  | SGSM1 | -0.859169868 | 3.20261E-09 |
|  | GPR158 | -0.65972096 | 3.30049E-09 |
|  | PCSK6 | -0.939022172 | 3.3212E-09 |
|  | TF | -0.80519884 | 3.36405E-09 |
|  | NKD1 | -0.611145325 | 3.50474E-09 |
|  | SETBP1 | -0.407738668 | 3.60086E-09 |
|  | P2RY13 | -0.551637744 | 3.60863E-09 |
|  | PPP1R9A | -0.516845209 | 3.6709E-09 |
|  | CACNA1D | -0.571059139 | 3.9351E-09 |
|  | STOX1 | -0.759830406 | 3.94636E-09 |
|  | GABBR1 | -0.692341111 | 3.97932E-09 |
|  | ATP7B | -0.400123678 | 4.07914E-09 |
|  | MAP7 | -0.576865892 | 4.20285E-09 |
|  | FAM107A | -0.593914222 | 4.23952E-09 |
|  | KIAA1462 | -0.465626878 | 4.47321E-09 |
|  | TRAM1L1 | -0.595480479 | 4.68995E-09 |
|  | LRRN1 | -0.514175169 | 4.79858E-09 |
|  | SLAIN1 | -0.44680249 | 4.96237E-09 |
|  | BMPR1B | -0.496341938 | 5.02574E-09 |
|  | IFIT3 | -0.473901761 | 5.2044E-09 |
|  | IGF1 | -0.499401135 | 5.50265E-09 |
|  | SCG3 | -0.780507581 | 5.75653E-09 |
|  | PLEKHH1 | -0.583104214 | 5.83869E-09 |
|  | C6orf174 | -0.496750157 | 6.48354E-09 |
|  | TMEM47 | -0.493088662 | 6.62852E-09 |
|  | BCHE | -0.428785007 | 6.69393E-09 |
|  | HEPH | -0.602514167 | 7.23677E-09 |
|  | KCNIP3 | -0.743799948 | 7.3545E-09 |
|  | CASS4 | -0.455620864 | 7.43472E-09 |
|  | MAPT | -0.515898 | 7.90149E-09 |
|  | VCAN | -0.472905398 | 7.94573E-09 |
|  | DNAH10 | -0.438841933 | 8.13958E-09 |
|  | PPP2R2C | -0.940630952 | 8.20459E-09 |
|  | C7orf58 | -0.564913422 | 8.21243E-09 |
|  | NMNAT2 | -0.809832705 | 8.22268E-09 |
|  | RAB3IP | -0.509618697 | 8.26965E-09 |
|  | MAN1A1 | -0.491582559 | 8.2864E-09 |
|  | RASGRF2 | -0.668646586 | 8.32914E-09 |
|  | MYCL1 | -0.581325113 | 8.63476E-09 |
|  | CNKSR2 | -0.610764806 | 9.22915E-09 |
|  | KIT | -0.632163266 | 9.88374E-09 |
|  | THRB | -0.495922629 | 1.09608E-08 |
|  | PRKCB | -0.736010607 | 1.11156E-08 |
|  | DNER | -0.424694572 | 1.11325E-08 |
|  | GRM1 | -0.79427249 | 1.22122E-08 |
|  | SAMD12 | -0.605386654 | 1.22257E-08 |
|  | LRRC4C | -0.473783152 | 1.24423E-08 |
|  | FGF12 | -0.644546861 | 1.33752E-08 |
|  | EVI2A | -0.542176984 | 1.49451E-08 |
|  | UNC80 | -0.65031286 | 1.59241E-08 |
|  | ABCA1 | -0.406928491 | 1.61397E-08 |
|  | IGSF1 | -0.887066037 | 1.6295E-08 |
|  | MID2 | -0.486701801 | 1.71515E-08 |
|  | SLC1A3 | -0.423011209 | 1.75035E-08 |
|  | MAOA | -0.428704916 | 1.77142E-08 |
|  | CHD7 | -0.493869035 | 1.78904E-08 |
|  | LRRK1 | -0.470034374 | 1.88689E-08 |
|  | SYT15 | -0.639473643 | 1.90513E-08 |
|  | ATP8B4 | -0.468318895 | 1.91352E-08 |
|  | RAPGEF5 | -0.61435995 | 1.98341E-08 |
|  | INPP4B | -0.487925144 | 2.04179E-08 |
|  | ATP13A4 | -0.542129983 | 2.28678E-08 |
|  | CLIC5 | -0.52631223 | 2.35965E-08 |
|  | THRA | -0.400545169 | 2.45133E-08 |
|  | PLXDC2 | -0.468066112 | 2.61555E-08 |
|  | KIF1A | -0.56952506 | 2.94297E-08 |
|  | ELFN2 | -1.006917526 | 3.12667E-08 |
|  | GPR34 | -0.537388848 | 3.14459E-08 |
|  | AQP4 | -0.631471047 | 3.22578E-08 |
|  | STARD4 | -0.513028089 | 3.30875E-08 |
|  | CXADR | -0.514352115 | 3.34511E-08 |
|  | DHDPSL | -0.561480102 | 3.42835E-08 |
|  | DOCK8 | -0.496590926 | 3.46546E-08 |
|  | MAGEE1 | -0.570118952 | 3.57204E-08 |
|  | SPTLC3 | -0.493855097 | 3.71666E-08 |
|  | SIGLEC8 | -0.574603677 | 3.85629E-08 |
|  | PRSS35 | -0.831957181 | 3.97016E-08 |
|  | CACNA2D2 | -0.716960368 | 4.2207E-08 |
|  | ANLN | -0.598600641 | 4.3819E-08 |
|  | CLSTN2 | -0.623916366 | 4.54924E-08 |
|  | FILIP1 | -0.422775665 | 4.81444E-08 |
|  | SLC14A1 | -1.162785779 | 5.01034E-08 |
|  | SCN3B | -0.844048717 | 5.04169E-08 |
|  | MYOM1 | -0.678936938 | 5.20341E-08 |
|  | INA | -1.121653221 | 5.35067E-08 |
|  | PSD2 | -0.602183655 | 5.37992E-08 |
|  | SLITRK5 | -0.672416009 | 5.43313E-08 |
|  | EFHA2 | -0.402916215 | 6.34441E-08 |
|  | RNF165 | -0.690146282 | 6.46365E-08 |
|  | CRY2 | -0.432034418 | 6.56858E-08 |
|  | MTAP | -0.423046558 | 7.00444E-08 |
|  | NTN4 | -0.704911055 | 7.1061E-08 |
|  | PCDHB19P | -0.482982845 | 8.06892E-08 |
|  | GHR | -0.472580076 | 8.41761E-08 |
|  | SLC1A2 | -0.684541664 | 8.44433E-08 |
|  | ZNF681 | -0.485265834 | 8.57738E-08 |
|  | EEF1A1P9 | -0.801655625 | 8.57983E-08 |
|  | PTCH1 | -0.548656255 | 8.61667E-08 |
|  | UST | -0.417794822 | 9.3322E-08 |
|  | OPCML | -0.725750988 | 9.36226E-08 |
|  | SYTL4 | -0.526782525 | 1.00824E-07 |
|  | MN1 | -0.666808331 | 1.03129E-07 |
|  | CRTAC1 | -0.958856111 | 1.05719E-07 |
|  | RASSF2 | -0.422345694 | 1.07199E-07 |
|  | RTN1 | -0.610937778 | 1.12286E-07 |
|  | PLEKHB1 | -0.434931356 | 1.15389E-07 |
|  | THSD4 | -0.641099881 | 1.24123E-07 |
|  | RFPL1S | -0.824470511 | 1.27288E-07 |
|  | PID1 | -0.479144462 | 1.30735E-07 |
|  | GNAO1 | -0.440062309 | 1.35992E-07 |
|  | MPEG1 | -0.482464077 | 1.41552E-07 |
|  | PFKFB2 | -0.402679222 | 1.43462E-07 |
|  | KIAA1377 | -0.564650095 | 1.50306E-07 |
|  | BMS1P5 | -0.442949364 | 1.54138E-07 |
|  | HSPA12A | -0.518846968 | 1.62878E-07 |
|  | CNTNAP2 | -0.836693018 | 1.66004E-07 |
|  | NACC2 | -0.407152359 | 1.67912E-07 |
|  | RHBDL3 | -0.571946371 | 1.68057E-07 |
|  | SNCAIP | -0.458504547 | 1.70648E-07 |
|  | ADHFE1 | -0.49309464 | 1.78345E-07 |
|  | KCNAB1 | -0.585118009 | 1.80837E-07 |
|  | PLCB4 | -0.502168137 | 1.89673E-07 |
|  | TEX9 | -0.432176091 | 1.94252E-07 |
|  | SLC30A10 | -0.419602571 | 2.17522E-07 |
|  | RGS9 | -0.69800498 | 2.23775E-07 |
|  | DTX4 | -0.460576633 | 2.28791E-07 |
|  | RAPGEF4 | -0.600803856 | 2.34622E-07 |
|  | MTHFD2 | -0.481625073 | 2.36872E-07 |
|  | GRIA2 | -0.633690182 | 2.39214E-07 |
|  | RAB11FIP4 | -0.568000735 | 2.43692E-07 |
|  | TEF | -0.448078091 | 2.44096E-07 |
|  | LY75 | -0.436130524 | 2.46921E-07 |
|  | TMTC1 | -0.420660242 | 2.69368E-07 |
|  | GRIA4 | -0.698157128 | 2.72538E-07 |
|  | HYDIN | -0.544779559 | 2.73581E-07 |
|  | LDB3 | -0.722314789 | 2.78288E-07 |
|  | GPLD1 | -0.46969595 | 2.82741E-07 |
|  | ATP2B2 | -0.554367897 | 2.87533E-07 |
|  | DCX | -0.850663114 | 2.92335E-07 |
|  | TMEM232 | -0.42358035 | 3.01231E-07 |
|  | DOCK3 | -0.490608506 | 3.05174E-07 |
|  | SEMA4D | -0.423429968 | 3.27109E-07 |
|  | SLCO1C1 | -0.570162336 | 3.27509E-07 |
|  | SUSD5 | -0.948583986 | 3.48636E-07 |
|  | TPPP | -0.700645526 | 3.55605E-07 |
|  | GABBR2 | -0.780928833 | 3.61214E-07 |
|  | GRIA3 | -0.411948914 | 4.36275E-07 |
|  | C10orf75 | -0.452622131 | 4.40139E-07 |
|  | FAIM2 | -0.492807481 | 4.53561E-07 |
|  | C1orf183 | -0.402074458 | 4.70098E-07 |
|  | BSN | -0.610525734 | 4.98225E-07 |
|  | VAT1L | -0.751411281 | 5.11848E-07 |
|  | DYNC1I1 | -0.632028403 | 5.42022E-07 |
|  | SPOCK1 | -0.604811434 | 5.58789E-07 |
|  | EFHD1 | -0.488640427 | 5.62661E-07 |
|  | ERC2 | -0.505951579 | 5.6752E-07 |
|  | ZNF365 | -0.446508967 | 5.93023E-07 |
|  | KCNMA1 | -0.407484418 | 6.32101E-07 |
|  | KCNIP2 | -0.794813246 | 6.38466E-07 |
|  | ZFPM2 | -0.650207017 | 6.38795E-07 |
|  | ENPP2 | -0.685422846 | 6.49708E-07 |
|  | LRP4 | -0.454859077 | 7.06204E-07 |
|  | FHDC1 | -0.611894313 | 7.46818E-07 |
|  | ABLIM3 | -0.495307976 | 7.84459E-07 |
|  | RND1 | -0.415213236 | 8.19983E-07 |
|  | PDZD2 | -0.49118435 | 8.34251E-07 |
|  | LONRF2 | -0.420315378 | 9.07195E-07 |
|  | ARHGEF37 | -0.41370702 | 9.26063E-07 |
|  | RIMS3 | -0.560023881 | 9.41197E-07 |
|  | GALNTL2 | -0.46519298 | 9.46531E-07 |
|  | KCNA2 | -0.476448341 | 9.7421E-07 |
|  | ACBD7 | -0.674596042 | 1.00571E-06 |
|  | DNAJC12 | -0.427514864 | 1.01261E-06 |
|  | LOC100190938 | -0.475042617 | 1.03456E-06 |
|  | PLSCR4 | -0.415738642 | 1.05164E-06 |
|  | NXPH1 | -0.695661838 | 1.08143E-06 |
|  | SCN2A | -0.544875204 | 1.1205E-06 |
|  | LRRTM2 | -0.472833849 | 1.13917E-06 |
|  | NRXN3 | -0.671688111 | 1.147E-06 |
|  | PDGFRA | -0.696388996 | 1.15397E-06 |
|  | HMP19 | -0.936312481 | 1.1638E-06 |
|  | LMO3 | -0.58314348 | 1.23545E-06 |
|  | CD24 | -0.712757608 | 1.24699E-06 |
|  | EBF1 | -0.427175573 | 1.37093E-06 |
|  | SLC25A18 | -0.471773118 | 1.42022E-06 |
|  | TEK | -0.423002745 | 1.48115E-06 |
|  | SLC1A1 | -0.53569454 | 1.66055E-06 |
|  | PDE10A | -0.499449034 | 1.66345E-06 |
|  | CADM3 | -0.577745117 | 1.78876E-06 |
|  | PRKCE | -0.412724753 | 2.00749E-06 |
|  | BASP1 | -0.486240598 | 2.03608E-06 |
|  | BEX2 | -0.452499472 | 2.0518E-06 |
|  | ST6GAL2 | -0.63825293 | 2.06018E-06 |
|  | FAM38B | -0.645127559 | 2.06775E-06 |
|  | CNTN4 | -0.674505952 | 2.08855E-06 |
|  | PCLO | -0.736568284 | 2.10746E-06 |
|  | BMP2 | -0.675578128 | 2.1391E-06 |
|  | RASGRP1 | -0.490994887 | 2.18218E-06 |
|  | RICH2 | -0.65118179 | 2.19851E-06 |
|  | CSF2RA | -0.437904846 | 2.24595E-06 |
|  | P2RY1 | -0.591248693 | 2.34634E-06 |
|  | PRKCQ | -0.46438856 | 2.62682E-06 |
|  | ERMN | -0.868132715 | 2.62682E-06 |
|  | SEMA4A | -0.443005254 | 2.7623E-06 |
|  | ABCA8 | -0.480636149 | 2.81247E-06 |
|  | CACNA1E | -0.665574464 | 3.07133E-06 |
|  | ZNF423 | -0.402712779 | 3.08363E-06 |
|  | SH3TC2 | -0.572450983 | 3.20271E-06 |
|  | WDFY4 | -0.4558165 | 3.37363E-06 |
|  | FAM189A2 | -0.482872448 | 3.598E-06 |
|  | CRB1 | -0.405436449 | 3.6573E-06 |
|  | LPCAT2 | -0.424421088 | 3.70703E-06 |
|  | DGCR6 | -0.458836248 | 4.07235E-06 |
|  | ENHO | -0.538885902 | 4.13014E-06 |
|  | TMEM144 | -0.655193538 | 4.61228E-06 |
|  | PTAFR | -0.510607324 | 4.74931E-06 |
|  | FAM65B | -0.454498682 | 4.81695E-06 |
|  | FGF1 | -0.437809999 | 4.89747E-06 |
|  | SYCP2 | -0.469702347 | 5.13272E-06 |
|  | MACROD2 | -0.537324739 | 5.46525E-06 |
|  | PLCH1 | -0.565403486 | 5.82366E-06 |
|  | SERPINI1 | -0.579705862 | 5.99003E-06 |
|  | KCNA6 | -0.443360231 | 6.2995E-06 |
|  | GRM3 | -0.5769057 | 6.45236E-06 |
|  | FAM19A2 | -0.695790095 | 6.60761E-06 |
|  | PEG3 | -0.549443833 | 6.81005E-06 |
|  | FAM84B | -0.444418599 | 7.17353E-06 |
|  | TMEM132B | -0.581194382 | 7.46206E-06 |
|  | LHFPL3 | -0.665499175 | 8.37488E-06 |
|  | NDN | -0.503675187 | 8.54587E-06 |
|  | MKRN3 | -0.455016269 | 8.6465E-06 |
|  | RNF182 | -0.41094456 | 9.02501E-06 |
|  | KIAA0513 | -0.408112537 | 9.03638E-06 |
|  | PTPRZ1 | -0.41115295 | 9.55788E-06 |
|  | SYT1 | -0.846276696 | 9.92723E-06 |
|  | ZDHHC22 | -0.672068141 | 1.07072E-05 |
|  | CCDC163P | -0.416456941 | 1.09129E-05 |
|  | NEGR1 | -0.611233228 | 1.09977E-05 |
|  | FRMPD1 | -0.522563821 | 1.35279E-05 |
|  | SLIT1 | -0.616101365 | 1.38144E-05 |
|  | PIK3CG | -0.471005776 | 1.40158E-05 |
|  | LOC254559 | -0.619381554 | 1.48371E-05 |
|  | JPH3 | -0.821692158 | 1.52677E-05 |
|  | TOX | -0.462479088 | 1.55154E-05 |
|  | SLC2A13 | -0.420629501 | 1.58223E-05 |
|  | AK5 | -0.730546966 | 1.58682E-05 |
|  | TMEM151B | -0.654234258 | 1.59329E-05 |
|  | ERBB3 | -0.582663986 | 1.82042E-05 |
|  | CDKN2B | -0.61016392 | 1.88434E-05 |
|  | PLEKHA6 | -0.43936507 | 1.88448E-05 |
|  | DACH1 | -0.488686418 | 1.91626E-05 |
|  | FLRT2 | -0.453886451 | 1.92987E-05 |
|  | EMX2OS | -0.56887817 | 2.01612E-05 |
|  | SMPD3 | -0.51165326 | 2.21432E-05 |
|  | DGKE | -0.430554728 | 2.30215E-05 |
|  | DIRAS2 | -0.499046788 | 2.30343E-05 |
|  | MEGF10 | -0.467616533 | 2.7285E-05 |
|  | TCEAL5 | -0.429358297 | 2.7939E-05 |
|  | BCAS1 | -0.651230294 | 2.84028E-05 |
|  | ZNF469 | -0.444710919 | 3.01894E-05 |
|  | SLC4A10 | -0.661715312 | 3.06291E-05 |
|  | KIAA1755 | -0.40009851 | 3.08212E-05 |
|  | ALDH1A1 | -0.461595155 | 3.17822E-05 |
|  | JPH4 | -0.574458276 | 3.34005E-05 |
|  | CASQ1 | -0.40270048 | 3.35087E-05 |
|  | CHGB | -0.704173778 | 3.55938E-05 |
|  | SCN4B | -0.429539523 | 3.84371E-05 |
|  | SLC6A17 | -0.778721042 | 3.86018E-05 |
|  | NWD1 | -0.581017432 | 3.95593E-05 |
|  | CABLES1 | -0.45440715 | 4.01525E-05 |
|  | STEAP2 | -0.487592189 | 4.67369E-05 |
|  | COL4A4 | -0.476038827 | 4.73327E-05 |
|  | SYN2 | -0.812465385 | 5.56411E-05 |
|  | LPL | -0.487079218 | 6.02406E-05 |
|  | FLRT1 | -0.500005724 | 6.10798E-05 |
|  | PTGDS | -0.529872169 | 6.80067E-05 |
|  | PDE2A | -0.529085514 | 7.87858E-05 |
|  | BCL11B | -0.443888793 | 7.9044E-05 |
|  | LGI3 | -0.577517272 | 8.00764E-05 |
|  | CX3CR1 | -0.512487049 | 8.12084E-05 |
|  | SEMA3G | -0.433911114 | 8.35271E-05 |
|  | CYP2J2 | -0.423706745 | 8.59342E-05 |
|  | SORCS1 | -0.532848363 | 9.65905E-05 |
|  | CYS1 | -0.474015218 | 0.000104206 |
|  | GRIN2C | -0.543080949 | 0.000104781 |
|  | GRIK2 | -0.480299197 | 0.000105086 |
|  | RASL10A | -0.614277078 | 0.000111924 |
|  | MFSD4 | -0.432545595 | 0.000116295 |
|  | DUSP26 | -0.486474224 | 0.00012795 |
|  | LOC339674 | -0.419452885 | 0.000133511 |
|  | MBP | -0.738564521 | 0.000161465 |
|  | AMZ1 | -0.508517626 | 0.000164068 |
|  | PCDHGC4 | -0.483299968 | 0.000170387 |
|  | SHISA9 | -0.603324117 | 0.000171204 |
|  | ODZ2 | -0.622235395 | 0.000176822 |
|  | GOLGA7B | -0.482898915 | 0.000179822 |
|  | NAPB | -0.420883259 | 0.000187392 |
|  | H2AFY2 | -0.498172764 | 0.00018847 |
|  | DGKG | -0.442213235 | 0.000189622 |
|  | PTCHD2 | -0.464668149 | 0.000192135 |
|  | MCTP1 | -0.449655898 | 0.000205218 |
|  | HS6ST3 | -0.649088025 | 0.000212565 |
|  | ATOH8 | -0.471077549 | 0.000231304 |
|  | SNAP25 | -0.663377 | 0.000250873 |
|  | CRYAB | -0.414550097 | 0.000255366 |
|  | CPS1 | -0.403963591 | 0.000258489 |
|  | EGR3 | -0.49762197 | 0.000338429 |
|  | UNC13A | -0.482561993 | 0.000347769 |
|  | SNCA | -0.43962174 | 0.000392531 |
|  | CHRNB2 | -0.459374387 | 0.000397176 |
|  | CARNS1 | -0.686609091 | 0.000422325 |
|  | SLC12A5 | -0.691058291 | 0.000456121 |
|  | SYNJ2 | -0.401116907 | 0.000502598 |
|  | NEFH | -0.532929275 | 0.000533272 |
|  | PCDHB4 | -0.435800965 | 0.000588729 |
|  | CDH13 | -0.402958403 | 0.000595152 |
|  | C1orf192 | -0.422305156 | 0.00060647 |
|  | TMEM130 | -0.631220172 | 0.000646561 |
|  | SHC3 | -0.500396415 | 0.000700731 |
|  | BEST3 | -0.448064331 | 0.000734858 |
|  | SPTB | -0.441715737 | 0.000763551 |
|  | C11orf9 | -0.53029482 | 0.000765004 |
|  | PTER | -0.479466794 | 0.000777398 |
|  | ATP1A3 | -0.479771801 | 0.000920446 |
|  | SULF1 | -0.529963961 | 0.001001693 |
|  | LHFPL4 | -0.407866139 | 0.001012816 |
|  | CAMK2A | -0.706117542 | 0.001028778 |
|  | CNTN2 | -0.539064312 | 0.001078898 |
|  | CHRDL1 | -0.484980499 | 0.001106398 |
|  | SLC45A3 | -0.417948807 | 0.001190229 |
|  | PCBP3 | -0.432658193 | 0.001234937 |
|  | DLL1 | -0.406380807 | 0.00130419 |
|  | CYP4X1 | -0.417882438 | 0.00152966 |
|  | ANK1 | -0.436834309 | 0.001693062 |
|  | PRDM8 | -0.426055159 | 0.001787141 |
|  | GPIHBP1 | -0.400862539 | 0.001820427 |
|  | DLL3 | -0.584215123 | 0.001841632 |
|  | KIAA0319 | -0.417830483 | 0.00189806 |
|  | NOG | -0.463465576 | 0.001985695 |
|  | RGS4 | -0.560802253 | 0.002244993 |
|  | SOX8 | -0.433755002 | 0.002721218 |
|  | CH25H | -0.437318606 | 0.002766446 |
|  | PHYHIP | -0.466712521 | 0.003185678 |
|  | AIFM3 | -0.508995331 | 0.003636298 |
|  | GAD1 | -0.403974448 | 0.003819899 |
|  | PSD | -0.42243587 | 0.004094821 |
|  | UNC5A | -0.43138864 | 0.004098181 |
|  | CLDN11 | -0.407446937 | 0.00519026 |
|  | SNCG | -0.424447914 | 0.006159681 |
|  | EGFR | -0.419327448 | 0.007722855 |
|  | DDN | -0.516321742 | 0.008508611 |
|  | L1CAM | -0.53190461 | 0.010297191 |
