# Supplementary material for: NR2F6, a new immune checkpoint that acts as a potential biomarker of immunosuppression and contributes to poor clinical outcome in human glioma
Source: Front Immunol. 2023 Jul 28;14:1139268. doi: 10.3389/fimmu.2023.1139268 (PMC10419227; doi:10.3389/fimmu.2023.1139268)
Supplement: Supplementary Table 4 — 772 DEGs in CGGA, including 375 upregulated and 397 downregulated genes. [file Table_4.docx]

| **Expression** | **Genes symbol** | **logFC** | **adj.P.Val** |
| --- | --- | --- | --- |
| **Upregulated** | MEX3D | 0.610888364 | 5.2561E-18 |
|  | CSNK1G2 | 0.428175564 | 2.63423E-17 |
|  | EVA1B | 0.938536748 | 5.48285E-15 |
|  | NXT1 | 0.547367483 | 1.73663E-14 |
|  | UBE2S | 0.764744448 | 4.69001E-14 |
|  | SYDE1 | 0.733247721 | 4.69001E-14 |
|  | TRAPPC5 | 0.439934445 | 6.13123E-14 |
|  | C19orf10 | 0.450423734 | 5.30666E-13 |
|  | SERPINH1 | 1.33555086 | 2.05263E-12 |
|  | SNAPC2 | 0.539780303 | 2.05263E-12 |
|  | MRPS12 | 0.491801092 | 2.71016E-12 |
|  | MRGBP | 0.440534702 | 2.88889E-12 |
|  | CCNB1 | 1.030572459 | 2.91921E-12 |
|  | CDR2 | 0.56143522 | 6.67125E-12 |
|  | MESDC1 | 0.50877763 | 1.2408E-11 |
|  | CHPF2 | 0.617507448 | 1.91307E-11 |
|  | SBNO2 | 0.671061006 | 2.30391E-11 |
|  | TACC3 | 1.002393614 | 2.88558E-11 |
|  | PRR7 | 0.635265392 | 3.54023E-11 |
|  | FBXW9 | 0.424828634 | 5.36073E-11 |
|  | GGH | 0.625028209 | 5.754E-11 |
|  | ZNF593 | 0.452580755 | 6.74314E-11 |
|  | LEPREL4 | 0.534213363 | 6.84612E-11 |
|  | H2AFX | 0.509062401 | 7.50286E-11 |
|  | ZNF771 | 0.410266442 | 8.44603E-11 |
|  | IGFBP2 | 1.914872278 | 1.12065E-10 |
|  | SPRY1 | 1.055337513 | 1.19205E-10 |
|  | C19orf24 | 0.45543311 | 1.31669E-10 |
|  | B3GALT6 | 0.407663542 | 1.79354E-10 |
|  | MFSD10 | 0.429458152 | 3.0502E-10 |
|  | PRC1 | 0.86317904 | 4.77145E-10 |
|  | CARHSP1 | 0.496649645 | 5.44275E-10 |
|  | TXNDC5 | 0.518923474 | 5.60444E-10 |
|  | GEMIN7 | 0.428445235 | 5.75182E-10 |
|  | CLEC11A | 0.631241791 | 6.0153E-10 |
|  | LEPRE1 | 0.659435279 | 6.17316E-10 |
|  | VKORC1 | 0.474740763 | 6.17316E-10 |
|  | CNIH4 | 0.440500417 | 7.49269E-10 |
|  | TGFB1I1 | 0.717988914 | 8.21407E-10 |
|  | PRR24 | 0.755830772 | 8.29752E-10 |
|  | MCM2 | 0.754500825 | 8.96956E-10 |
|  | DDOST | 0.404619122 | 9.0279E-10 |
|  | DHRS13 | 0.465198484 | 1.06213E-09 |
|  | CKAP4 | 0.478011438 | 1.18602E-09 |
|  | PLOD1 | 0.676523518 | 1.21481E-09 |
|  | LYPLA2 | 0.412950495 | 1.26788E-09 |
|  | UBALD2 | 0.526523669 | 1.33412E-09 |
|  | MCAM | 0.825306813 | 1.4928E-09 |
|  | RP5-940J5,9 | 0.724568322 | 1.90152E-09 |
|  | VEGFA | 1.488254793 | 1.95216E-09 |
|  | EMILIN1 | 0.989443801 | 2.2125E-09 |
|  | TMEM2 | 0.687039018 | 2.25102E-09 |
|  | IPO4 | 0.41841023 | 2.3346E-09 |
|  | AP2S1 | 0.461558691 | 2.39668E-09 |
|  | TPI1 | 0.477185535 | 3.48118E-09 |
|  | SNRPB | 0.474687682 | 3.79787E-09 |
|  | HDAC7 | 0.423018227 | 4.33796E-09 |
|  | RGS3 | 0.652568258 | 4.42073E-09 |
|  | COL6A2 | 1.463680319 | 4.58635E-09 |
|  | PUS7 | 0.411085045 | 4.67356E-09 |
|  | ORAI1 | 0.406789548 | 4.67356E-09 |
|  | PMM2 | 0.47536552 | 4.70296E-09 |
|  | COL4A2 | 1.431727463 | 5.26115E-09 |
|  | CD97 | 0.733357294 | 5.27185E-09 |
|  | TPGS1 | 0.442016596 | 5.63555E-09 |
|  | MYO1B | 0.817354196 | 5.85775E-09 |
|  | DUSP6 | 0.811443332 | 6.15779E-09 |
|  | SLC29A1 | 0.468955696 | 7.17911E-09 |
|  | PDIA4 | 0.606781973 | 7.1807E-09 |
|  | COL6A1 | 0.831413545 | 7.39469E-09 |
|  | SMC4 | 0.754025465 | 7.44201E-09 |
|  | SLC4A2 | 0.442512129 | 7.49359E-09 |
|  | PVR | 0.43483927 | 7.98265E-09 |
|  | SSBP4 | 0.403982962 | 8.39181E-09 |
|  | PFN1 | 0.444907697 | 8.55514E-09 |
|  | TSPAN9 | 0.476103785 | 9.44665E-09 |
|  | CDK2 | 0.700372568 | 1.00006E-08 |
|  | FAM20C | 0.882611597 | 1.10946E-08 |
|  | PRDX4 | 0.473399417 | 1.11512E-08 |
|  | TGIF1 | 0.71885597 | 1.35642E-08 |
|  | TIMP1 | 1.845854388 | 1.39307E-08 |
|  | CDKN2C | 0.895558633 | 1.40558E-08 |
|  | CD276 | 0.687527655 | 1.56493E-08 |
|  | LAMB1 | 1.042063986 | 1.60624E-08 |
|  | FAM126A | 0.56524533 | 1.65549E-08 |
|  | IMPDH1 | 0.404881476 | 1.73863E-08 |
|  | KLF16 | 0.44452507 | 1.77311E-08 |
|  | KPNA2 | 0.526260028 | 1.77943E-08 |
|  | KDELR2 | 0.469657647 | 1.9898E-08 |
|  | ITGA5 | 0.966818364 | 2.20109E-08 |
|  | PDLIM7 | 0.679951574 | 2.26464E-08 |
|  | OSTC | 0.493916296 | 2.46936E-08 |
|  | LMNB2 | 0.50649891 | 2.63386E-08 |
|  | CD151 | 0.599905551 | 2.7233E-08 |
|  | PCOLCE | 0.990977485 | 2.75792E-08 |
|  | ITGB3BP | 0.507894253 | 2.911E-08 |
|  | ARL4C | 0.815873952 | 3.23437E-08 |
|  | TPM4 | 0.601582351 | 3.26525E-08 |
|  | RECQL4 | 0.625918417 | 3.27289E-08 |
|  | CHPF | 0.558337252 | 3.32528E-08 |
|  | FEN1 | 0.421243297 | 3.36225E-08 |
|  | MANF | 0.461830483 | 4.03115E-08 |
|  | TUBB6 | 0.847238026 | 4.35158E-08 |
|  | ZYX | 0.60722055 | 4.50706E-08 |
|  | NAB2 | 0.448188641 | 4.50706E-08 |
|  | DSN1 | 0.45125155 | 4.7907E-08 |
|  | CALU | 0.647075455 | 4.85011E-08 |
|  | C19orf48 | 0.447459529 | 5.29878E-08 |
|  | LAMC1 | 0.849744549 | 5.4631E-08 |
|  | ECM1 | 0.643222382 | 6.19168E-08 |
|  | CBR3 | 0.484786744 | 7.16938E-08 |
|  | WDR34 | 0.401013195 | 7.37114E-08 |
|  | FURIN | 0.411309629 | 7.51405E-08 |
|  | SEC24D | 0.597101895 | 7.71759E-08 |
|  | SMYD2 | 0.590308184 | 7.75693E-08 |
|  | PLOD3 | 0.486865093 | 7.88885E-08 |
|  | CKS2 | 0.723691832 | 7.91906E-08 |
|  | PGK1 | 0.515805052 | 8.40306E-08 |
|  | IFNGR2 | 0.447261987 | 8.4707E-08 |
|  | LDHA | 0.818722077 | 8.70964E-08 |
|  | CHAF1A | 0.522859074 | 9.19848E-08 |
|  | ISYNA1 | 0.588067837 | 9.42125E-08 |
|  | PTP4A3 | 0.596513268 | 9.6952E-08 |
|  | TMEM160 | 0.701651692 | 9.70549E-08 |
|  | TSKU | 0.47469329 | 9.81871E-08 |
|  | BZW2 | 0.425320926 | 9.83358E-08 |
|  | EFNB1 | 0.464661163 | 1.12272E-07 |
|  | PLP2 | 0.988156167 | 1.16391E-07 |
|  | MIF | 0.446185684 | 1.16391E-07 |
|  | PMEPA1 | 0.549663475 | 1.18873E-07 |
|  | GNG5 | 0.587097273 | 1.19501E-07 |
|  | MCM6 | 0.421053638 | 1.19501E-07 |
|  | RNASEH2A | 0.499804876 | 1.24198E-07 |
|  | TUBB4B | 0.414785549 | 1.28249E-07 |
|  | EFNB2 | 0.665724056 | 1.32195E-07 |
|  | FLNA | 0.771124568 | 1.43914E-07 |
|  | CITED2 | 0.496255067 | 1.53554E-07 |
|  | GAPDH | 0.569983902 | 1.55783E-07 |
|  | SCARF2 | 0.466769364 | 1.55783E-07 |
|  | STK40 | 0.49808052 | 1.57285E-07 |
|  | SLC20A1 | 0.504948952 | 1.69811E-07 |
|  | DNAJB1 | 0.513601189 | 1.78116E-07 |
|  | CDCA7L | 0.679522487 | 1.8348E-07 |
|  | COL1A2 | 1.347592187 | 1.93403E-07 |
|  | JUNB | 0.823941842 | 1.99803E-07 |
|  | YBX3 | 0.660060363 | 2.13104E-07 |
|  | EFEMP2 | 0.851390435 | 2.31201E-07 |
|  | MAP2K3 | 0.534503374 | 2.37302E-07 |
|  | HSPA5 | 0.426802395 | 2.40571E-07 |
|  | IGFBP4 | 0.797587818 | 2.56088E-07 |
|  | NDUFA4L2 | 0.767004733 | 2.78476E-07 |
|  | KANK2 | 0.560292016 | 3.06732E-07 |
|  | FN1 | 1.055345981 | 3.54571E-07 |
|  | TSPAN4 | 0.41869014 | 3.635E-07 |
|  | PLXDC1 | 0.597903038 | 3.64105E-07 |
|  | TFRC | 0.468389298 | 4.13312E-07 |
|  | LAMA4 | 0.662558252 | 4.47087E-07 |
|  | FZD1 | 0.484475332 | 4.64633E-07 |
|  | GBE1 | 0.515826965 | 4.73849E-07 |
|  | CEBPB | 0.67728676 | 4.88318E-07 |
|  | COL18A1 | 0.698166022 | 5.14904E-07 |
|  | PTK7 | 0.477919874 | 5.24749E-07 |
|  | CHST2 | 0.597801425 | 5.55975E-07 |
|  | SLC9A1 | 0.480700121 | 5.57529E-07 |
|  | POLD1 | 0.477869997 | 5.64581E-07 |
|  | FNDC3B | 0.532743714 | 6.35306E-07 |
|  | CALD1 | 0.586403009 | 6.82292E-07 |
|  | AC010441,1 | 0.749347032 | 8.44609E-07 |
|  | COLGALT1 | 0.433567153 | 8.52667E-07 |
|  | PTGFRN | 0.653295078 | 8.83104E-07 |
|  | FSTL1 | 0.770388747 | 8.88138E-07 |
|  | MRC2 | 0.852009475 | 8.89172E-07 |
|  | TTYH3 | 0.469232042 | 9.28111E-07 |
|  | BCAM | 0.488277184 | 9.33135E-07 |
|  | PCNA | 0.425054377 | 9.65572E-07 |
|  | TMEM106C | 0.447271248 | 1.10782E-06 |
|  | PRSS23 | 0.804129532 | 1.12089E-06 |
|  | VASP | 0.522953011 | 1.16562E-06 |
|  | MMP14 | 0.900435394 | 1.22228E-06 |
|  | IER5 | 0.419640981 | 1.3134E-06 |
|  | BACE2 | 0.650634568 | 1.327E-06 |
|  | CLIC1 | 0.823391573 | 1.38058E-06 |
|  | RHBDF1 | 0.510217685 | 1.45292E-06 |
|  | TNFAIP8L1 | 0.45258295 | 1.51391E-06 |
|  | MXRA7 | 0.423020176 | 1.68373E-06 |
|  | LPHN2 | 0.578772319 | 1.98481E-06 |
|  | C1orf85 | 0.474199323 | 1.99049E-06 |
|  | HIST1H1C | 0.64699495 | 2.36975E-06 |
|  | OAF | 0.596877317 | 2.45606E-06 |
|  | MIR22HG | 0.552902855 | 2.46033E-06 |
|  | SHC1 | 0.551800542 | 2.46764E-06 |
|  | LBH | 0.55340915 | 2.5339E-06 |
|  | CUL7 | 0.442685659 | 2.55426E-06 |
|  | SPAG5 | 0.548415575 | 2.57428E-06 |
|  | P4HB | 0.44045763 | 2.61354E-06 |
|  | ECE1 | 0.509334266 | 2.80711E-06 |
|  | DCBLD2 | 0.564336669 | 2.84961E-06 |
|  | ERRFI1 | 0.48821328 | 2.90439E-06 |
|  | ANXA1 | 1.187529818 | 2.94238E-06 |
|  | STK17A | 0.47102629 | 3.16392E-06 |
|  | EPHB4 | 0.486290126 | 3.42694E-06 |
|  | SLC25A19 | 0.415246981 | 3.78581E-06 |
|  | RACGAP1 | 0.464821636 | 3.82492E-06 |
|  | FKBP10 | 0.569617312 | 3.9144E-06 |
|  | ATF5 | 0.538192845 | 4.11587E-06 |
|  | BICD1 | 0.401689332 | 4.42082E-06 |
|  | RAB34 | 0.783127339 | 4.7826E-06 |
|  | ITPKC | 0.505190715 | 4.86659E-06 |
|  | METTL1 | 0.655858363 | 4.95395E-06 |
|  | ACTN1 | 0.783181985 | 4.99299E-06 |
|  | METRNL | 0.578077255 | 5.04701E-06 |
|  | CAV1 | 0.816068295 | 5.15036E-06 |
|  | CLEC14A | 0.451651339 | 5.16942E-06 |
|  | SPRY2 | 0.487464293 | 5.19844E-06 |
|  | ENG | 0.528653975 | 5.76014E-06 |
|  | SLC2A4RG | 0.450740631 | 6.02675E-06 |
|  | TAGLN2 | 0.806924203 | 6.29413E-06 |
|  | SPRED2 | 0.410170341 | 6.89786E-06 |
|  | IER2 | 0.531626515 | 6.96485E-06 |
|  | FHOD1 | 0.4437046 | 7.10263E-06 |
|  | SLC39A8 | 0.506773511 | 7.16278E-06 |
|  | GADD45A | 0.664428904 | 8.08659E-06 |
|  | TAGLN | 0.872635675 | 8.61944E-06 |
|  | RRAS | 0.491793278 | 9.31621E-06 |
|  | NAMPT | 0.825985151 | 9.84376E-06 |
|  | NT5DC2 | 0.453647417 | 1.03244E-05 |
|  | ERO1L | 0.405485716 | 1.03881E-05 |
|  | MYADM | 0.53159445 | 1.1012E-05 |
|  | VIM | 0.903481542 | 1.18577E-05 |
|  | HIST2H2AA4 | 0.6196316 | 1.23402E-05 |
|  | PTRF | 0.679473829 | 1.26398E-05 |
|  | BGN | 0.792350074 | 1.30458E-05 |
|  | SMIM4 | 0.401472985 | 1.32691E-05 |
|  | RCC1 | 0.430104984 | 1.33674E-05 |
|  | TM4SF1 | 0.681050535 | 1.33798E-05 |
|  | AKAP2 | 0.450458576 | 1.37539E-05 |
|  | TUBA1C | 0.785831164 | 1.39713E-05 |
|  | ITGA3 | 0.676233138 | 1.4286E-05 |
|  | CHIC2 | 0.478418365 | 1.50136E-05 |
|  | UACA | 0.431285213 | 1.50577E-05 |
|  | TRIP10 | 0.438173234 | 1.50991E-05 |
|  | MTMR11 | 0.459423995 | 1.57431E-05 |
|  | CASP7 | 0.40906032 | 1.61879E-05 |
|  | CAV2 | 0.633886435 | 1.70505E-05 |
|  | FHL3 | 0.422538098 | 1.75215E-05 |
|  | CCDC3 | 0.572559059 | 1.82144E-05 |
|  | TMSB10 | 0.603393801 | 1.82286E-05 |
|  | LAMB2 | 0.491812155 | 2.01703E-05 |
|  | CDH5 | 0.433608383 | 2.24199E-05 |
|  | RBMS1 | 0.486473317 | 2.26903E-05 |
|  | SLC27A3 | 0.522503604 | 2.31679E-05 |
|  | AEN | 0.40460666 | 2.53606E-05 |
|  | HIST1H2BK | 0.565972125 | 2.59283E-05 |
|  | FKBP11 | 0.428719497 | 2.62111E-05 |
|  | VMP1 | 0.429098921 | 2.64147E-05 |
|  | FKBP9 | 0.624712311 | 2.72304E-05 |
|  | LGALS3 | 0.86657006 | 2.77984E-05 |
|  | HILPDA | 0.562157816 | 2.97856E-05 |
|  | ESAM | 0.44037467 | 3.05399E-05 |
|  | RCN3 | 0.587336317 | 3.2154E-05 |
|  | SLC16A3 | 0.691783959 | 3.26758E-05 |
|  | LMNA | 0.413877752 | 3.35652E-05 |
|  | SMS | 0.402109263 | 3.43643E-05 |
|  | HMGB2 | 0.458422485 | 3.49937E-05 |
|  | SLC2A3 | 0.59448377 | 3.50391E-05 |
|  | HSPB1 | 0.601646435 | 3.61722E-05 |
|  | LGALS1 | 0.686525829 | 3.63562E-05 |
|  | KLF10 | 0.464516694 | 3.70747E-05 |
|  | TNFRSF14 | 0.495280516 | 3.82744E-05 |
|  | ACTA2 | 0.714042553 | 4.03236E-05 |
|  | MICALL2 | 0.550278704 | 4.20554E-05 |
|  | AKAP12 | 0.62779557 | 4.26715E-05 |
|  | PPP1R15A | 0.472590008 | 4.63158E-05 |
|  | ITGB1 | 0.45325468 | 4.66437E-05 |
|  | CD34 | 0.465580958 | 4.79767E-05 |
|  | PVRL2 | 0.436289156 | 5.09979E-05 |
|  | PDGFA | 0.551471308 | 5.45093E-05 |
|  | CCDC85B | 0.40883983 | 6.13228E-05 |
|  | NRP1 | 0.543077542 | 6.23572E-05 |
|  | C16orf93 | 0.441400857 | 6.36536E-05 |
|  | COL5A3 | 0.554660241 | 6.73298E-05 |
|  | PLOD2 | 0.543411641 | 6.73298E-05 |
|  | MYL9 | 0.630795978 | 6.78792E-05 |
|  | CTSK | 0.589741179 | 6.79963E-05 |
|  | NOTCH3 | 0.45220775 | 6.79963E-05 |
|  | TMSB4X | 0.503573749 | 6.8142E-05 |
|  | KNTC1 | 0.420593333 | 7.46631E-05 |
|  | IRF1 | 0.51334619 | 7.47949E-05 |
|  | JUN | 0.466241409 | 7.47949E-05 |
|  | LMNB1 | 0.589398242 | 7.8885E-05 |
|  | CAPN5 | 0.464733902 | 8.14654E-05 |
|  | EIF4EBP1 | 0.436837671 | 8.23634E-05 |
|  | NID2 | 0.47278043 | 8.66232E-05 |
|  | PDGFRB | 0.463078351 | 8.73345E-05 |
|  | FOSL2 | 0.555876404 | 9.57852E-05 |
|  | IQGAP1 | 0.47542001 | 9.65706E-05 |
|  | TRIB2 | 0.440387659 | 0.000102223 |
|  | ACVRL1 | 0.409392583 | 0.000104564 |
|  | PIM1 | 0.424554696 | 0.000106136 |
|  | PYGL | 0.562715485 | 0.000107449 |
|  | EGR1 | 0.776915883 | 0.000109505 |
|  | EMP1 | 0.701568305 | 0.000111521 |
|  | ANXA2 | 0.864466365 | 0.000116538 |
|  | PLEKHG2 | 0.434060319 | 0.000119543 |
|  | NFATC4 | 0.446286431 | 0.000120083 |
|  | HES1 | 0.434627946 | 0.000121838 |
|  | GSDMD | 0.506854299 | 0.000130215 |
|  | PLK3 | 0.444834858 | 0.000135116 |
|  | MYL12A | 0.507168415 | 0.000136404 |
|  | EHD2 | 0.48318862 | 0.000144404 |
|  | PARVB | 0.426545562 | 0.000146155 |
|  | VWF | 0.489946803 | 0.00015299 |
|  | CASP4 | 0.584834485 | 0.000167694 |
|  | ARID5A | 0.402202877 | 0.000174651 |
|  | TCIRG1 | 0.489054009 | 0.000186655 |
|  | UPP1 | 0.581756189 | 0.000196141 |
|  | TNC | 0.710662107 | 0.000258662 |
|  | SOCS2 | 0.579281698 | 0.000282638 |
|  | RBPMS | 0.484664319 | 0.000315914 |
|  | KLF2 | 0.428868776 | 0.000327045 |
|  | RDH10 | 0.624361725 | 0.000342185 |
|  | IGFBP7 | 0.452894466 | 0.00035443 |
|  | TPM2 | 0.460740751 | 0.000359871 |
|  | FABP5 | 0.885788843 | 0.000360587 |
|  | ITGA7 | 0.439452435 | 0.000579122 |
|  | PLS3 | 0.401196929 | 0.000652572 |
|  | PSRC1 | 0.453195569 | 0.000655051 |
|  | HMOX1 | 0.670977036 | 0.000695613 |
|  | PLIN2 | 0.501657476 | 0.000738015 |
|  | RP11-386G11,10 | 0.541420967 | 0.000761919 |
|  | RCAN1 | 0.495349625 | 0.000778878 |
|  | MMP2 | 0.459578196 | 0.000808705 |
|  | S100A11 | 0.607397778 | 0.000916735 |
|  | DYNLT3 | 0.490652012 | 0.000959859 |
|  | CDK4 | 0.496155016 | 0.001002161 |
|  | SEC61G | 0.637737538 | 0.001012711 |
|  | PLK2 | 0.42423684 | 0.00115936 |
|  | SERPING1 | 0.616594486 | 0.001226144 |
|  | RFTN1 | 0.418399733 | 0.001272649 |
|  | PLSCR1 | 0.429379716 | 0.001321743 |
|  | SERPINF1 | 0.469615766 | 0.001543922 |
|  | MEST | 0.420617326 | 0.001585017 |
|  | MT2A | 0.492356296 | 0.001585689 |
|  | C4orf48 | 0.44471256 | 0.001595939 |
|  | IGFBP5 | 0.579440966 | 0.001656547 |
|  | TRIP6 | 0.402840252 | 0.001707902 |
|  | DCN | 0.517256552 | 0.001778615 |
|  | MSN | 0.491963968 | 0.001928034 |
|  | IFITM3 | 0.518086918 | 0.002130078 |
|  | C1QTNF1 | 0.479664948 | 0.002146301 |
|  | BHLHE40 | 0.451204743 | 0.002382535 |
|  | MARCH9 | 0.404876871 | 0.002386432 |
|  | HSPA1B | 0.471794408 | 0.00243707 |
|  | IFI30 | 0.645474042 | 0.002724856 |
|  | MDK | 0.521593635 | 0.003151113 |
|  | DUSP1 | 0.489410183 | 0.003159888 |
|  | C1R | 0.61012391 | 0.003275285 |
|  | WWTR1 | 0.444153177 | 0.003464873 |
|  | RND3 | 0.41454025 | 0.003690417 |
|  | VWA1 | 0.422489631 | 0.003898461 |
|  | GAP43 | 0.45065408 | 0.004058437 |
|  | FILIP1L | 0.404956984 | 0.004444452 |
|  | NRN1 | 0.479973851 | 0.004454865 |
|  | SOD2 | 0.576445994 | 0.004659377 |
|  | FOS | 0.534618402 | 0.004877813 |
|  | ATF3 | 0.451041751 | 0.005166427 |
|  | S1PR3 | 0.435258994 | 0.00623393 |
|  | C10orf10 | 0.431296585 | 0.006795576 |
|  | GADD45B | 0.407869929 | 0.00847126 |
|  | EFEMP1 | 0.488276802 | 0.009612589 |
|  | FABP7 | 0.489063046 | 0.011609385 |
|  | CTGF | 0.403719454 | 0.016048293 |
|  | ISG15 | 0.406848543 | 0.019101195 |
|  | AEBP1 | 0.52370289 | 0.019957927 |
|  | SPP1 | 0.524545802 | 0.040387874 |
| **Downregulated** | PEA15 | -0.792581168 | 1.64177E-12 |
|  | NT5C2 | -0.470323624 | 1.64177E-12 |
|  | ADD3 | -0.790454189 | 2.05263E-12 |
|  | ATP9A | -0.83570047 | 2.10676E-12 |
|  | HIPK2 | -0.825958278 | 2.71016E-12 |
|  | OSBPL11 | -0.705959391 | 3.03178E-12 |
|  | C20orf194 | -0.484165014 | 4.35047E-12 |
|  | ATM | -0.434507737 | 6.82716E-12 |
|  | C22orf46 | -0.51692797 | 8.92899E-12 |
|  | KIAA1109 | -0.46582936 | 2.30391E-11 |
|  | DOCK10 | -0.638451186 | 4.4275E-11 |
|  | TBC1D5 | -0.50888678 | 4.4275E-11 |
|  | SLC39A11 | -0.522011085 | 6.74314E-11 |
|  | ABLIM1 | -0.634383397 | 1.79354E-10 |
|  | KLHL5 | -0.451788169 | 2.42817E-10 |
|  | GCLC | -0.578019311 | 2.57481E-10 |
|  | WDFY3 | -0.545682042 | 2.61921E-10 |
|  | NTRK2 | -1.176810047 | 2.75764E-10 |
|  | DOPEY1 | -0.43221018 | 2.84098E-10 |
|  | THRA | -0.778723421 | 2.88443E-10 |
|  | CTNND2 | -0.74117687 | 3.0239E-10 |
|  | C7orf41 | -1.023201491 | 3.95921E-10 |
|  | ALDH2 | -0.786325303 | 3.95921E-10 |
|  | ANKFY1 | -0.422267114 | 4.96031E-10 |
|  | SLC23A2 | -0.452583064 | 5.10248E-10 |
|  | AKAP6 | -0.702518778 | 6.44293E-10 |
|  | CLASP2 | -0.684413249 | 6.44293E-10 |
|  | FBXW4 | -0.527288858 | 7.05639E-10 |
|  | ASTN2 | -0.635989537 | 7.49269E-10 |
|  | MGEA5 | -0.427905302 | 8.13296E-10 |
|  | MXI1 | -0.609939151 | 8.21407E-10 |
|  | USP54 | -0.826719359 | 8.27003E-10 |
|  | PDE4DIP | -0.582392844 | 8.29752E-10 |
|  | SLC9A9 | -0.492730431 | 8.29752E-10 |
|  | AP2B1 | -0.429307351 | 8.29752E-10 |
|  | EPB41L2 | -0.495446836 | 8.43636E-10 |
|  | IKZF5 | -0.437723589 | 9.36594E-10 |
|  | CEP350 | -0.416755731 | 1.05526E-09 |
|  | SORBS1 | -0.588189348 | 1.18312E-09 |
|  | TOM1L2 | -0.515806993 | 1.18602E-09 |
|  | ALDH6A1 | -0.722143846 | 1.24194E-09 |
|  | RND2 | -0.836687767 | 1.26788E-09 |
|  | PRKAG2 | -0.50440895 | 1.26788E-09 |
|  | KIF1A | -0.969427729 | 1.33412E-09 |
|  | LPIN1 | -0.463467921 | 1.42692E-09 |
|  | SNHG14 | -0.55893833 | 1.78648E-09 |
|  | RCBTB1 | -0.469546369 | 2.34229E-09 |
|  | DST | -0.438219127 | 2.34229E-09 |
|  | WASF3 | -0.802658612 | 2.57716E-09 |
|  | SCD5 | -0.807705431 | 2.64668E-09 |
|  | LIMCH1 | -0.593102661 | 2.86046E-09 |
|  | NEBL | -0.902766678 | 3.4623E-09 |
|  | TJP1 | -0.420142501 | 3.46657E-09 |
|  | KMT2A | -0.46991944 | 3.54935E-09 |
|  | ACADSB | -0.458865067 | 3.86635E-09 |
|  | FCHSD2 | -0.577859482 | 4.16739E-09 |
|  | ACBD5 | -0.4886225 | 4.33796E-09 |
|  | GNAO1 | -0.865854856 | 4.42073E-09 |
|  | MAPRE2 | -0.415106338 | 4.70826E-09 |
|  | PAAF1 | -0.433101956 | 4.88312E-09 |
|  | OGFRL1 | -0.772377764 | 4.90937E-09 |
|  | PLEKHB1 | -0.873782328 | 5.21523E-09 |
|  | SLC1A4 | -0.759067517 | 5.21523E-09 |
|  | PEAK1 | -0.422413823 | 5.30011E-09 |
|  | SAMD8 | -0.475707884 | 5.85775E-09 |
|  | RRAGD | -0.43200629 | 7.38785E-09 |
|  | INPP1 | -0.44641043 | 7.39469E-09 |
|  | KIAA1737 | -0.437564773 | 8.08181E-09 |
|  | ZFYVE16 | -0.460537907 | 8.28633E-09 |
|  | ANGEL1 | -0.444872403 | 8.28633E-09 |
|  | BAZ2B | -0.415334291 | 9.11358E-09 |
|  | PER3 | -0.554378675 | 9.41567E-09 |
|  | PPP1R12B | -0.401060323 | 9.58234E-09 |
|  | MARCH8 | -0.568219572 | 9.6741E-09 |
|  | MYCBP2 | -0.481929452 | 9.79895E-09 |
|  | CAB39L | -0.586777603 | 1.10946E-08 |
|  | MAP3K5 | -0.618779758 | 1.11512E-08 |
|  | DCLK2 | -0.620571468 | 1.15923E-08 |
|  | ASCC1 | -0.419313911 | 1.15923E-08 |
|  | MAP4 | -0.402135747 | 1.22085E-08 |
|  | ALDH5A1 | -0.695294821 | 1.23148E-08 |
|  | LRIG1 | -0.707668965 | 1.38673E-08 |
|  | C1orf198 | -0.609219266 | 1.54378E-08 |
|  | CHST15 | -0.55081042 | 1.60624E-08 |
|  | SCD | -0.888070424 | 1.66785E-08 |
|  | EPHX1 | -0.4836307 | 1.6694E-08 |
|  | PIK3IP1 | -0.524986873 | 1.6898E-08 |
|  | SNN | -0.539349096 | 1.69887E-08 |
|  | NCAM1 | -0.705526669 | 1.70307E-08 |
|  | RAB30 | -0.470355361 | 1.73216E-08 |
|  | VPS13D | -0.610612537 | 1.88128E-08 |
|  | SORL1 | -0.587369333 | 1.89555E-08 |
|  | MAPK10 | -0.532049466 | 1.90948E-08 |
|  | ARHGAP12 | -0.501887081 | 1.97469E-08 |
|  | sept-03 | -0.930308838 | 1.9898E-08 |
|  | GLUD1 | -0.697208938 | 2.2016E-08 |
|  | ATP6V1G2 | -0.976592948 | 2.33644E-08 |
|  | KIAA1147 | -0.432803949 | 2.45545E-08 |
|  | sept-08 | -0.583030306 | 2.73433E-08 |
|  | ZMYND11 | -0.497924816 | 2.83056E-08 |
|  | RNF141 | -0.461153106 | 2.83056E-08 |
|  | DHTKD1 | -0.452559625 | 2.92287E-08 |
|  | ZFYVE20 | -0.428762149 | 3.07817E-08 |
|  | KIAA1755 | -0.63599572 | 3.08114E-08 |
|  | MT-ND5 | -0.518233417 | 3.78091E-08 |
|  | SOX2-OT | -0.61598471 | 3.94191E-08 |
|  | RASSF4 | -0.594317839 | 4.1711E-08 |
|  | ITPK1 | -0.587139841 | 4.64283E-08 |
|  | FAM13C | -0.662231372 | 4.7907E-08 |
|  | BEX4 | -0.515754549 | 4.85011E-08 |
|  | DZIP3 | -0.409323606 | 4.85011E-08 |
|  | PHLPP1 | -0.544890674 | 4.89253E-08 |
|  | HDAC4 | -0.516599992 | 5.10055E-08 |
|  | KIF3A | -0.507709762 | 5.10272E-08 |
|  | IGIP | -0.58324716 | 5.1654E-08 |
|  | FTO | -0.417918315 | 5.1654E-08 |
|  | ARL3 | -0.46871742 | 5.18108E-08 |
|  | EFHD1 | -0.897382342 | 5.35889E-08 |
|  | FMN2 | -0.548913677 | 5.40182E-08 |
|  | LHPP | -0.924033104 | 6.13025E-08 |
|  | GPM6B | -0.532960209 | 6.20033E-08 |
|  | NAP1L3 | -0.724301971 | 6.23568E-08 |
|  | HMGN5 | -0.546473978 | 6.98074E-08 |
|  | DGCR6 | -0.973086732 | 7.07603E-08 |
|  | ARNT2 | -0.521992472 | 7.07603E-08 |
|  | AHCYL1 | -0.654857085 | 7.51405E-08 |
|  | NF1 | -0.43050854 | 7.52356E-08 |
|  | TMOD2 | -0.609761368 | 7.63814E-08 |
|  | FADS1 | -0.537716268 | 7.8006E-08 |
|  | CPE | -0.870947717 | 8.33586E-08 |
|  | GPRC5B | -0.635042339 | 9.08973E-08 |
|  | FAM171A1 | -0.576215236 | 9.6272E-08 |
|  | FMNL2 | -0.423273017 | 1.00599E-07 |
|  | NDRG2 | -1.050450332 | 1.02516E-07 |
|  | KIF1B | -0.475657158 | 1.0991E-07 |
|  | MAP1A | -0.628114982 | 1.15607E-07 |
|  | CCSER2 | -0.430816175 | 1.23572E-07 |
|  | CSGALNACT1 | -0.575258494 | 1.27644E-07 |
|  | PPM1K | -0.530330494 | 1.30354E-07 |
|  | USP46 | -0.532347803 | 1.32276E-07 |
|  | TTYH2 | -0.667965225 | 1.34204E-07 |
|  | MFSD6 | -0.53466876 | 1.42639E-07 |
|  | GABBR1 | -0.830793137 | 1.43283E-07 |
|  | TRIM2 | -0.518300867 | 1.50552E-07 |
|  | EEPD1 | -0.692854604 | 1.52811E-07 |
|  | ADIPOR2 | -0.430434648 | 1.7933E-07 |
|  | PIP4K2A | -0.680380621 | 1.83559E-07 |
|  | AHNAK | -0.55561841 | 1.85704E-07 |
|  | ABAT | -0.745896498 | 1.93403E-07 |
|  | TMCC3 | -0.437983315 | 2.06665E-07 |
|  | PPP2R5A | -0.538804082 | 2.07569E-07 |
|  | CNRIP1 | -0.587145406 | 2.0936E-07 |
|  | PPP2R2B | -0.48184653 | 2.0936E-07 |
|  | TRIM16L | -0.412274587 | 2.11558E-07 |
|  | ALDOC | -1.077714201 | 2.15937E-07 |
|  | HSPB8 | -0.912922693 | 2.20526E-07 |
|  | ABCG1 | -0.486941458 | 2.30112E-07 |
|  | NTM | -0.620589879 | 2.33118E-07 |
|  | KIF1C | -0.463366841 | 2.33118E-07 |
|  | DPYSL3 | -0.498142209 | 2.36562E-07 |
|  | FADS2 | -0.735111288 | 2.37472E-07 |
|  | FOXO4 | -0.488790645 | 2.40206E-07 |
|  | C14orf132 | -0.670184588 | 2.4224E-07 |
|  | FHL1 | -0.472542181 | 2.43851E-07 |
|  | CRYL1 | -0.444591547 | 2.46299E-07 |
|  | ACO2 | -0.419473891 | 2.52176E-07 |
|  | FAM69A | -0.512971839 | 2.53283E-07 |
|  | MICAL3 | -0.561117981 | 2.5868E-07 |
|  | RAB6B | -0.612177722 | 2.63759E-07 |
|  | KIF2A | -0.439009165 | 2.68966E-07 |
|  | PAQR8 | -0.76209527 | 2.73828E-07 |
|  | PARD3 | -0.401739026 | 2.85283E-07 |
|  | SULT1C4 | -0.618167384 | 2.99152E-07 |
|  | BDNF-AS | -0.452641725 | 3.0867E-07 |
|  | NDRG3 | -0.405455678 | 3.14288E-07 |
|  | SALL2 | -0.521880776 | 3.15228E-07 |
|  | RAP2A | -0.541921137 | 3.16844E-07 |
|  | ZCCHC24 | -0.563391001 | 3.24336E-07 |
|  | SLC9A6 | -0.438150828 | 3.41391E-07 |
|  | PC | -0.410681677 | 3.49538E-07 |
|  | LDLRAD4 | -0.460485275 | 3.68938E-07 |
|  | PKP4 | -0.521732185 | 4.13935E-07 |
|  | SECISBP2L | -0.43185402 | 4.18757E-07 |
|  | TPCN1 | -0.440704069 | 4.34612E-07 |
|  | PSAT1 | -0.653670728 | 4.64633E-07 |
|  | CAMK2G | -0.619998263 | 5.27078E-07 |
|  | PPP1R3E | -0.488288586 | 5.28987E-07 |
|  | PLP1 | -1.326472824 | 5.42545E-07 |
|  | PTGDS | -1.169244972 | 5.60599E-07 |
|  | SLAIN1 | -0.66752567 | 5.72005E-07 |
|  | TOB2 | -0.413633014 | 5.84419E-07 |
|  | MAPT | -0.806061903 | 6.04743E-07 |
|  | GRAMD3 | -0.552999536 | 6.04743E-07 |
|  | PRKX | -0.469996198 | 6.11834E-07 |
|  | ABHD6 | -0.491635514 | 6.27944E-07 |
|  | PHGDH | -0.56195476 | 6.35306E-07 |
|  | ANKRD6 | -0.440813373 | 6.36468E-07 |
|  | ARRB1 | -0.596499028 | 6.81452E-07 |
|  | TSPAN7 | -0.721642752 | 7.0552E-07 |
|  | SYBU | -0.631284818 | 7.32074E-07 |
|  | ALCAM | -0.647072484 | 7.4867E-07 |
|  | RICTOR | -0.40407292 | 7.55222E-07 |
|  | CMTM4 | -0.552794569 | 8.01725E-07 |
|  | PRKCA | -0.439110103 | 8.88138E-07 |
|  | LPHN3 | -0.549609789 | 1.00456E-06 |
|  | KIAA0930 | -0.466329459 | 1.02249E-06 |
|  | RFTN2 | -0.583846926 | 1.06855E-06 |
|  | QKI | -0.451915552 | 1.08987E-06 |
|  | ELMO1 | -0.672258589 | 1.17856E-06 |
|  | CBX7 | -0.576297807 | 1.2197E-06 |
|  | HDAC11 | -0.545811639 | 1.2701E-06 |
|  | WNK1 | -0.400668164 | 1.37005E-06 |
|  | SATB1 | -0.543611188 | 1.37253E-06 |
|  | FAIM2 | -0.754598244 | 1.3735E-06 |
|  | SEMA4D | -0.603194863 | 1.437E-06 |
|  | NAV1 | -0.428791774 | 1.74922E-06 |
|  | NRXN2 | -0.664399727 | 1.86979E-06 |
|  | SLC6A1 | -0.765280265 | 1.90443E-06 |
|  | RIN2 | -0.425736707 | 1.92286E-06 |
|  | ZMIZ1 | -0.405802699 | 1.93441E-06 |
|  | TSC22D4 | -0.564673337 | 1.94406E-06 |
|  | CCBL1 | -0.420712965 | 1.97481E-06 |
|  | ZBTB47 | -0.415581272 | 2.03989E-06 |
|  | MTSS1L | -0.601604682 | 2.12234E-06 |
|  | KB-1507C5,2 | -0.424928965 | 2.21206E-06 |
|  | PHACTR1 | -0.55564522 | 2.42013E-06 |
|  | ZBTB18 | -0.451550385 | 2.46033E-06 |
|  | SASH1 | -0.433552692 | 2.49209E-06 |
|  | FAM107A | -0.915375867 | 2.77842E-06 |
|  | DDAH1 | -0.469207546 | 2.89073E-06 |
|  | PFN2 | -0.452124588 | 3.09435E-06 |
|  | RTN1 | -1.039617991 | 3.09564E-06 |
|  | ZHX2 | -0.413371475 | 3.23188E-06 |
|  | ARHGAP32 | -0.44047618 | 3.35561E-06 |
|  | RP11-396K3,1 | -0.432129203 | 3.42694E-06 |
|  | PRKACB | -0.469771156 | 3.50008E-06 |
|  | HNMT | -0.428352751 | 3.72023E-06 |
|  | TF | -1.079633058 | 3.86604E-06 |
|  | OSGIN2 | -0.463366956 | 3.94563E-06 |
|  | MPP2 | -0.434548227 | 3.94563E-06 |
|  | RPS6KL1 | -0.51086325 | 4.30307E-06 |
|  | FAM107B | -0.549089583 | 4.48715E-06 |
|  | RASSF2 | -0.588859684 | 4.68451E-06 |
|  | RRP7B | -0.437962756 | 4.71785E-06 |
|  | RTN3 | -0.432774178 | 4.89716E-06 |
|  | CNP | -0.623172155 | 5.00259E-06 |
|  | PDLIM5 | -0.444433045 | 5.18108E-06 |
|  | sept-04 | -0.793485557 | 5.86562E-06 |
|  | LSAMP | -0.498457761 | 6.17806E-06 |
|  | TEF | -0.543832827 | 6.33885E-06 |
|  | ARHGAP31 | -0.415959422 | 7.66087E-06 |
|  | LANCL1 | -0.447024798 | 7.83446E-06 |
|  | DICER1-AS1 | -0.45877867 | 8.00067E-06 |
|  | SCHIP1 | -0.449788898 | 8.29792E-06 |
|  | BIN1 | -0.470877487 | 8.64593E-06 |
|  | MTND2P28 | -0.611446375 | 8.65519E-06 |
|  | HRSP12 | -0.562277841 | 9.07034E-06 |
|  | ASIC1 | -0.653371512 | 9.1795E-06 |
|  | ZMAT1 | -0.407612841 | 9.41807E-06 |
|  | RP11-617F23,1 | -0.435293535 | 9.55112E-06 |
|  | IDNK | -0.40485157 | 9.6181E-06 |
|  | FAXDC2 | -0.467788689 | 9.95323E-06 |
|  | NIPA1 | -0.461330144 | 1.11688E-05 |
|  | APCDD1 | -0.496439532 | 1.11858E-05 |
|  | B3GAT1 | -0.601793584 | 1.12277E-05 |
|  | PLXNB1 | -0.440925485 | 1.13862E-05 |
|  | C10orf54 | -0.481753967 | 1.1925E-05 |
|  | TNK2 | -0.618815744 | 1.19692E-05 |
|  | ARHGEF4 | -0.509722307 | 1.24716E-05 |
|  | PRTFDC1 | -0.411650247 | 1.27078E-05 |
|  | FAM213A | -0.462604524 | 1.28042E-05 |
|  | SLC22A17 | -0.484351061 | 1.34284E-05 |
|  | LRRC8A | -0.430473845 | 1.44232E-05 |
|  | GRIA2 | -0.761487397 | 1.45341E-05 |
|  | SEC31B | -0.418076533 | 1.59929E-05 |
|  | ANKRD46 | -0.407106155 | 1.60944E-05 |
|  | SYTL4 | -0.57038218 | 1.63704E-05 |
|  | PDZD4 | -0.655981145 | 1.69911E-05 |
|  | SLC44A1 | -0.515500883 | 1.7241E-05 |
|  | KIF5C | -0.586012565 | 1.85627E-05 |
|  | LCAT | -0.52028137 | 1.87468E-05 |
|  | KLHL25 | -0.466802223 | 1.91165E-05 |
|  | SESN1 | -0.429133521 | 1.93816E-05 |
|  | PIK3R1 | -0.473818264 | 1.95881E-05 |
|  | MTUS1 | -0.42020491 | 1.97642E-05 |
|  | TMCC2 | -0.538998254 | 2.12224E-05 |
|  | GRIA3 | -0.453316201 | 2.26554E-05 |
|  | CAPN3 | -0.86861145 | 2.31679E-05 |
|  | DAPK1 | -0.444142197 | 2.4099E-05 |
|  | LIFR | -0.538681588 | 2.44879E-05 |
|  | ST6GAL1 | -0.433863 | 2.55624E-05 |
|  | APC2 | -0.567310831 | 2.58867E-05 |
|  | SLC48A1 | -0.433688399 | 2.92103E-05 |
|  | MBP | -1.488017477 | 2.95568E-05 |
|  | S1PR1 | -0.450034429 | 2.96295E-05 |
|  | TMEM63A | -0.571171227 | 2.98184E-05 |
|  | MT-RNR1 | -0.4706491 | 3.11852E-05 |
|  | NFASC | -0.421116727 | 3.13616E-05 |
|  | LRRC4 | -0.50768926 | 3.4176E-05 |
|  | PLSCR4 | -0.452516653 | 3.43419E-05 |
|  | GPM6A | -0.552876428 | 3.49695E-05 |
|  | AMOTL2 | -0.511454488 | 3.49903E-05 |
|  | HIP1R | -0.613208238 | 3.52559E-05 |
|  | DTNA | -0.415533609 | 3.6556E-05 |
|  | SREBF1 | -0.460887494 | 3.72597E-05 |
|  | METTL7A | -0.483105337 | 3.78412E-05 |
|  | EEF1A1P5 | -0.438477456 | 3.92322E-05 |
|  | MYO10 | -0.441368911 | 3.96708E-05 |
|  | CRYAB | -0.740797548 | 4.06711E-05 |
|  | GDAP1 | -0.422391938 | 4.27443E-05 |
|  | KIAA0513 | -0.48667887 | 4.44368E-05 |
|  | LIMS2 | -0.583924013 | 4.63158E-05 |
|  | RASA4B | -0.484546831 | 5.02124E-05 |
|  | IL17D | -0.6091821 | 5.37329E-05 |
|  | APOE | -0.513104468 | 5.5923E-05 |
|  | SPARCL1 | -0.562442501 | 5.65231E-05 |
|  | ASRGL1 | -0.400722987 | 5.69364E-05 |
|  | FXYD6 | -0.695160679 | 5.8996E-05 |
|  | SERP2 | -0.51501174 | 6.05611E-05 |
|  | CRY2 | -0.42598544 | 6.29884E-05 |
|  | RASA4 | -0.440696522 | 6.45422E-05 |
|  | DOCK9 | -0.424480476 | 6.74686E-05 |
|  | GFAP | -0.659849108 | 6.94749E-05 |
|  | FAM171B | -0.46642366 | 7.28425E-05 |
|  | NDRG4 | -0.554271108 | 7.77207E-05 |
|  | TSPYL2 | -0.445628484 | 7.91742E-05 |
|  | UST | -0.504660935 | 8.4904E-05 |
|  | CTNNA2 | -0.446859942 | 8.62099E-05 |
|  | ROGDI | -0.410418248 | 0.000103256 |
|  | PID1 | -0.596951238 | 0.00010865 |
|  | NFIB | -0.428881714 | 0.000111442 |
|  | MAP2 | -0.552368608 | 0.000111611 |
|  | LIPE | -0.583128925 | 0.000122873 |
|  | ABCB1 | -0.402887008 | 0.000123072 |
|  | CLDND1 | -0.45424702 | 0.000132648 |
|  | PFKFB2 | -0.402735053 | 0.000133303 |
|  | KIF5A | -0.769295381 | 0.000139808 |
|  | EDNRB | -0.610821379 | 0.000140297 |
|  | TIMP3 | -0.421610148 | 0.000144861 |
|  | PCDHGC3 | -0.498654292 | 0.000156252 |
|  | PMP2 | -0.573281414 | 0.000177604 |
|  | FOLR2 | -0.573623734 | 0.000186918 |
|  | BCR | -0.432845119 | 0.000188032 |
|  | PLEKHG3 | -0.419443566 | 0.000217134 |
|  | MTSS1 | -0.425926095 | 0.000237265 |
|  | AGT | -0.583184189 | 0.000243584 |
|  | ATP1A2 | -0.806921074 | 0.000284197 |
|  | IFIT2 | -0.410434749 | 0.000305402 |
|  | EPB41L3 | -0.512791641 | 0.000307071 |
|  | SPOCK2 | -0.457780008 | 0.000339506 |
|  | FAM110B | -0.501886352 | 0.000379096 |
|  | AQP4 | -0.717352175 | 0.000395684 |
|  | SCAMP5 | -0.419438229 | 0.000436615 |
|  | CBS | -0.457974243 | 0.000471384 |
|  | RGCC | -0.436557372 | 0.000482899 |
|  | HES6 | -0.730285283 | 0.000484577 |
|  | TMEM47 | -0.489441991 | 0.00048929 |
|  | DBNDD2 | -0.56802035 | 0.000513141 |
|  | C1orf61 | -0.600255076 | 0.000561689 |
|  | ARRDC2 | -0.45745692 | 0.000576569 |
|  | LRRN1 | -0.562522053 | 0.000669167 |
|  | APLP1 | -0.508228623 | 0.00068476 |
|  | SYP | -0.621366758 | 0.000743486 |
|  | RAB40B | -0.41490002 | 0.000780392 |
|  | PKIA | -0.417723244 | 0.00078698 |
|  | MTHFD2 | -0.488065929 | 0.000799027 |
|  | SULF2 | -0.433063782 | 0.000961208 |
|  | BCAN | -0.818755454 | 0.00101321 |
|  | RGMB | -0.424977648 | 0.001038837 |
|  | KAL1 | -0.422917078 | 0.001058412 |
|  | ATP1B1 | -0.51513093 | 0.00128387 |
|  | STXBP1 | -0.462426344 | 0.001353166 |
|  | ABCA2 | -0.422202608 | 0.001413038 |
|  | S100A1 | -0.677690466 | 0.001477724 |
|  | LRP4 | -0.419197844 | 0.001522093 |
|  | CSF1R | -0.407192145 | 0.001642638 |
|  | C15orf59 | -0.533616054 | 0.001760057 |
|  | LINGO1 | -0.538166186 | 0.002058757 |
|  | LPL | -0.572066089 | 0.003143782 |
|  | TCF12 | -0.436604263 | 0.003306968 |
|  | ID4 | -0.423190101 | 0.003642335 |
|  | FGF1 | -0.456184627 | 0.003803748 |
|  | ANLN | -0.471586453 | 0.003932945 |
|  | IFIT1 | -0.4148503 | 0.004544694 |
|  | BASP1 | -0.452206272 | 0.004695404 |
|  | TTYH1 | -0.406058511 | 0.004801781 |
|  | NAPB | -0.465819955 | 0.005099017 |
|  | RN7SL2 | -0.578706719 | 0.005768491 |
|  | ANGPTL2 | -0.403972621 | 0.005901998 |
|  | MLC1 | -0.407956512 | 0.006765486 |
|  | MAP6D1 | -0.413730451 | 0.007988583 |
|  | CDK18 | -0.446685365 | 0.009067621 |
|  | C4A | -0.445047682 | 0.013850129 |
|  | APOC1 | -0.406766194 | 0.01490161 |
|  | RN7SL1 | -0.448262275 | 0.021426295 |
|  | PPP1R14A | -0.436235844 | 0.031721171 |
|  | RAB3A | -0.425985107 | 0.032892542 |
